# Synthesis and Bioactivity Assessment of Novel Quinolinone–Triazole Hybrids

**DOI:** 10.3390/biom16010029

**Published:** 2025-12-24

**Authors:** Ioanna Kostopoulou, Maria-Anna Karadendrou, Manolis Matzapetakis, Maria Zervou, Georgia-Eirini Deligiannidou, Christos Kontogiorgis, Eleni Pontiki, Dimitra Hadjipavlou-Litina, Anastasia Detsi

**Affiliations:** 1 Laboratory of Organic Chemistry, Department of Chemical Sciences, School of Chemical Engineering, National Technical University of Athens, Heroon Polytechniou 9, Zografou Campus, 15780 Athens, Greece; ioanna.th.kostopoulou@gmail.com (I.K.); mariannakaradendrou@mail.ntua.gr (M.-A.K.); 2 Institute of Chemical Biology, National Hellenic Research Foundation, 48 Vas. Constantinou Ave., 11635 Athens, Greece; matzman@eie.gr (M.M.); mzervou@eie.gr (M.Z.); 3 Laboratory of Hygiene and Environmental Protection, Department of Medicine, Democritus University of Thrace, 68100 Alexandroupolis, Greece; edeligia@med.duth.gr (G.-E.D.); ckontogi@med.duth.gr (C.K.); 4 Laboratory of Pharmaceutical Chemistry, School of Pharmacy, Faculty of Health Sciences, Aristotle University of Thessaloniki, 54124 Thessaloniki, Greece; epontiki@pharm.auth.gr (E.P.); hadjipav@pharm.auth.gr (D.H.-L.)

**Keywords:** quinolinone, triazole, click chemistry, CuAAC reaction, microwave irradiation, antioxidant activity, lipoxygenase inhibition, cytotoxicity

## Abstract

Click chemistry, and particularly the Cu-catalyzed Azide Alkyne Cycloaddition (CuAAC) reaction has gained increased attention in recent years as an invaluable tool for synthesizing pharmaceutical active organic compounds. In this study, quinolinones and triazoles, two bioactive heterocyclic moieties amenable to various substitutions, were employed to design and synthesize novel quinolinone–triazole hybrid molecules via the CuAAC click reaction under microwave irradiation. The synthesized hybrid molecules and their alkyne precursors were structurally characterized and evaluated for their antioxidant capacity, lipoxygenase (LOX) inhibitory activity, cell viability using HaCaT epithelial cells, and cytotoxicity against two cancer lines. The results indicated that, among the precursors, alkyne **4c** exhibits the best combined antioxidant and anti-inflammatory activity (100% lipid peroxidation inhibition, IC_50_ = 22.5 μM for LOX inhibition); among the hybrid molecules, compound **5a** was the most potent (98.0% lipid peroxidation inhibition, IC_50_ = 10.0 μM for LOX inhibition). Regarding the assessment of HaCaT cell viability, all studied compounds showed encouraging results, with cell viability rates between 61.5% and 100%. Moreover, based on the results of the cytotoxicity against cancer lines A549 and A375, it emerged that the tested compounds exhibited moderate–low or no cytotoxicity. These results highlight the potential of quinolinone–triazole hybrids as valuable candidates in drug discovery.

## 1. Introduction

In recent years, click chemistry has been proven to be a powerful tool for the development of innovative and effective strategies for the synthesis of lead compounds of significant interest to the pharmaceutical industry. Click chemistry was introduced for the first time by the Nobelist K. Barry Sharpless in 2001 [1] and has since been used to describe a group of reactions that are thermodynamically favored, reliable, and stereoselective, efficiently leading to the formation of complex molecules via carbon–heteroatom bond formation. [2,3,4,5]. Among these reactions, the Copper-Catalyzed Azide Alkyne Cycloaddition (CuAAC) reaction, which affords 1,2,3-triazoles, is one of the most well-known representatives of click chemistry [6,7].

The CuAAC reaction exhibits significant advantages, such as being carried out at ambient temperature or mild heating, regiospecificity (as only 1,4-di-substituted isomers of triazole are formed), resistance to water and oxygen, simplicity and ease in processing and receiving the product, as well as its low cost [8,9]. In addition to these reasons, the popularity of this reaction can also be attributed to the starting materials themselves. Both the azides and alkynes can be easily used in the reaction due to their great stability under a wide range of conditions: both can tolerate oxygen, water, a broad range of solvents and pH values, as well as biological molecules or other functional groups that are in the reaction, without interacting with them [8,10,11].

**Figure 1:**
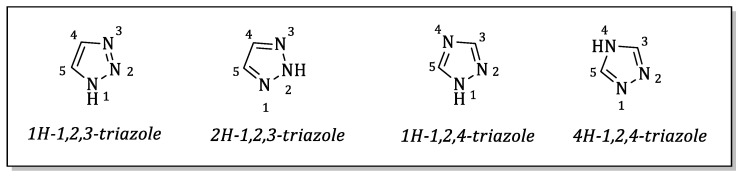
Triazole isomers.

Triazoles are heterocyclic compounds, which consist of a five-membered di-unsaturated ring of two carbon atoms and three nitrogen atoms and appear in the form of two possible isomers, 1,2,3-triazoles and 1,2,4-triazoles (Figure 1) [12].

Triazoles are notable for their strong dipole moment, electron-deficient aromatic structure, as well as for their capacity to act as hydrogen bond acceptors. These properties, combined with their robust resistance to chemical and metabolic degradation, make them versatile bioisosteres for functional groups, such as amides, esters, carboxylic acids and olefinic double bonds [13,14]. Due to these characteristics, the triazole nucleus appears in many pharmacologically active compounds, exhibiting multifaceted pharmacological properties [15,16,17], such as anti-inflammatory [18], antioxidant [19,20,21,22], anticancer [23], antiviral [24], and neuroprotective properties [25].

**Figure 2:**
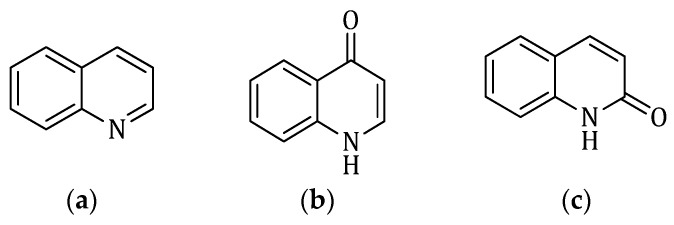
General structure of quinoline (**a**), 4-quinolone (**b**), and 2-quinolone (**c**).

Within the class of heterocyclic compounds, quinolines and quinolinones (Figure 2) are considered “privileged” structures and are widely used as building blocks in the development of new pharmaceuticals. The scaffold of these heterocyclic compounds, comprising a benzene ring fused with a pyridine ring, renders them substantially versatile. They are found in numerous bioactive compounds of synthetic as well as natural origin and their derivatives have shown efficacy as antioxidant, anti-inflammatory, antispasmodic, antimalarial, antifungal, antitubercular, antiviral, antibacterial, and analgesic agents [26,27].

Motivated by the pursuit of novel pharmaceutical agents with combined and improved properties, researchers’ interest has turned to the combination of various bioactive structural units in one hybrid molecule, aiming to derive multi-target drugs for the treatment of complex and multifactorial diseases. Recently, a variety of multi-target molecules containing the cinnamic acid moiety has been reported [28,29]. The strategic hybridization of bioactive moieties in organic synthesis often enhances pharmacokinetic properties, stability, and biological efficacy by allowing the synergy of multiple functional groups in a single compound [30].

In this context, the combination of quinoline, quinolone, or quinolinone structural units with triazoles to form hybrid molecules is often found in the literature [31]. These molecules have been proven to be therapeutically active, presenting important biological properties, such as antimalarial [32], antimicrobial [33,34,35], anticancer [36], antileishmanial [37], antitubercular [38,39], antiviral [40], and antioxidant properties [41].

Lipoxygenases (LOXs) are a family of iron containing enzymes widely distributed in nature, which play vital roles in various physiological processes, including the regulation of vascular, renal, gastrointestinal and skin functions, as well as immune responses. Inhibitors of LOX activity are attracting interest due to the role of this enzymes in the arachidonic acid cascade inducing inflammation and various other related pathological conditions [42,43].

Chronic inflammation and oxidative stress are interconnected factors that contribute in the pathogenesis of diseases like diabetes, cancer, and cardiovascular disorders [44]. Oxidative stress results from an imbalance between antioxidants and reactive oxygen species (ROS), caused by either reduced antioxidant defenses or ROS overproduction. As a result, the ability of endogenous antioxidant enzymes to combat ROS is reduced, thereby contributing to tissue damage [45,46]. Exogenous antioxidants prevent or delay the oxidation of macromolecules by inhibiting the formation of free radicals by scavenging them or preventing their propagation. Thus, designing new drugs that integrate both anti-inflammatory and antioxidant activities may offer therapeutic benefits for a wide range of diseases [47].

Moreover, persistent chronic inflammation contributes to cancer development and may predispose people to carcinogenesis. Infection-induced inflammation is involved in the pathogenesis of approximately 20% of human tumors. Furthermore, even tumors that are not epidemiologically linked to pathogens are characterized by the presence of an inflammatory component in their microenvironment [48,49]. In light of this, numerous studies have investigated the link between LOX inhibitors and carcinogenesis, identifying novel targets for cancer therapy.

In this framework and as a continuation of our research [22,50,51,52], the current project is directed towards the design, synthesis and structural characterization of novel quinolinone–triazole hybrid molecules, via the CuAAC click reaction. In addition, the synthesized quinolinone derivatives are evaluated regarding their bioactivity, in terms of their antioxidant capacity, through their ability to inhibit lipid peroxidation induced by 2,2′-Azobis(2-amidinopropane) dihydrochloride (AAPH). Inhibition of Soybean LOX was also determined, considering these results as a possible indicator of their anti-inflammatory activity. Furthermore, their cytotoxicity in two cancer lines was measured.

## 2. Materials and Methods

The chemicals used for the synthesis of the novel compounds were purchased from Sigma-Aldrich (Burlington, MA, USA), Fluka (Buchs, Switzerland), Alfa-Aesar (Lancashire, UK) and Acros (Fukuoka, Japan) and were used without further purification. The synthesized compounds were structurally characterized using 300 MHz and 600 MHz Varian NMR spectrometer using DMSO-d_6_ and CDCl_3_-d_1_ 99.9 atom % D as solvents. ^1^HNMR spectra were acquired at 298 K using a spectral width of −2–20 ppm and 32 number of scans. Coupling constants (*J*) are reported in Hertz (Hz) and chemical shifts (*δ*) are given in parts per million (ppm) units, relative to the solvent. Spectra processing was performed using MestReNova version number11.0.0 software. HR-MS spectra were recorded on a UHPLC-MSn Orbitrap Velos-Thermo mass spectrometer (Thermo Scientific, Waltham, MA, USA). FTIR spectroscopy was performed on a JASCO FT/IR-4200 spectrometer (JASCO, Easton, MD, USA) in the form of KBr pellets, in the scanning range of 400–4000 cm^−1^. Melting points were measured on a Gallenkamp MFB-595 melting point apparatus and are reported uncorrected. The used cell lines in our experiments were a kind offer from Associate Professor A. Pappa (Department of Molecular Biology & Genetics, Faculty of Health Sciences, Democritus University of Thrace, 68100 Alexandroupolis, Greece): (I) The human cancer cell lines A549 (non-small cell lung adenocarcinoma, ATCC CCL-185) were obtained from the American Type Culture Collection (Rockville, MD, USA). (ΙΙ) The A375 cells were obtained from the American Type Culture Collection (ATCC; Cat No: CRL-1619, Manassas, VA, USA). (IIΙ) The human immortalized keratinocyte (HaCaT) cell line was kindly provided by Dr Sharon Broby (Dermal Toxicology & Effects Group; Centre for Radiation, Chemical and Environmental Hazards; Public Health England, Didcot, UK).

### 2.1. Synthesis and General Procedures

#### 2.1.1. General Method (A): Synthesis of N-Substituted Isatoic Anhydrides (**2a–2g**)

The desired alkyl halide was added to a stirred solution of isatoic anhydride (**1**) and sodium hydride (NaH), in dry dimethylformamide (DMF), under cooling. The reaction mixture was then refluxed or stirred at room temperature for 24 h, under inert atmosphere and monitored by Thin-Layer Chromatography (TLC). Upon completion, the mixture was poured into a conical flask containing water and ice and then extracted three times with diethyl ether (Et_2_O). The combined organic phases were dried over anhydrous sodium sulfate (Na_2_SO_4_) and concentrated under reduced pressure. The final product (**2**) was obtained as a solid after additional rinsing with Et_2_O and used in the next step without further purification.

*1-methyl-1H-benzo[d][1,3]oxazine-2,4-dione* (**2a**)

Prepared according to the general method (A), isatoic anhydride (**1**) (5 g, 30.6 mmol) was added at room temperature to a mixture of NaH (1.1 g, 45.9 mol, 60% *w*/*w* in oil) and 125 mL dry DMF, followed by the addition of iodomethane (2.3 mL, 36.7 mmol) at 0 °C. The product (**2a**) was obtained after the appropriate treatment in the form of a beige solid. Yield: 64% (3.729 g). ^1^H NMR (600 MHz, DMSO-d_6_) *δ* (ppm) 8.00 (dd, *J* = 7.8, 1.2 Hz, 1H, Ar-H), 7.78 (td, *J* = 8.4, 1.2 Hz, 1H, Ar-H), 7.44 (d, *J* = 8.4 Hz, 1H, Ar-H), 7.34 (d, *J* = 7.2 Hz, 1H, Ar-H), 3.46 (s, 3H, N-CH_3_); ^13^C NMR (150 MHz, DMS-d_6_) *δ* (ppm) 159.0, 147.7, 142.2, 137.2, 129.3, 123.6, 114.8, 111.5, 31.7.

*1-benzyl-1H-benzo[d][1,3]oxazine-2,4-dione* (**2b**)

Prepared according to the general method (A), isatoic anhydride (**1**) (3 g, 18.4 mmol) was added at room temperature to a mixture of NaH (662.4 mg, 27.6 mol, 60% *w*/*w* in oil) and 75 mL dry DMF, followed by the addition of benzyl bromide (2.6 mL, 24.6 mmol) at 0 °C. The product (**2b**) was obtained after the appropriate treatment in the form of a beige solid. Yield: 51% (2.3842 g). ^1^H NMR (600 MHz, DMSO-d_6_) *δ* (ppm) 8.04 (dd, *J* = 7.8, 1.8 Hz, 1H, Ar-H), 7.63 (td, *J* = 8.4, 1.2 Hz, 1H, Ar-H), 7.41 (d, *J* = 7.8 Hz, 2H, Ar-H), 7.34 (t, *J* = 7.8 Hz, 2H, Ar-H), 7.31 (t, *J* = 7.8 Hz, 1H, Ar-H), 7.27 (t, *J* = 7.2 Hz, 1H, Ar-H), 7.24 (d, *J* = 8.4 Hz, 1H, Ar-H), 5.29 (s, 2H, N-CH_2_-Ar); ^13^C NMR (150 MHz, DMSO-d_6_) *δ* (ppm) 158.9, 148.3, 141.4, 137.0, 135.3, 129.5, 128.6, 127.4, 126.6, 123.7, 115.1, 112.1, 47.6.

*1-ethyl-1H-benzo[d][1,3]oxazine-2,4-dione* (**2c**)

Prepared according to the general method (A), isatoic anhydride (**1**) (2 g, 12.3 mmol) was added at room temperature to a mixture of NaH (444 mg, 18.5 mol, 60% *w*/*w* in oil) and 50 mL dry DMF, followed by the addition of iodoethane (1.2 mL, 14.8 mmol) at 0 °C. The product (**2c**) was obtained after the appropriate treatment in the form of a beige solid. Yield: 36% (840 mg). ^1^H NMR (600 MHz, DMSO-d_6_) *δ* (ppm) 8.0 (d, *J* = 7.8, 1H, Ar-H), 7.85 (t, *J* = 7.2 Hz, 1H, Ar-H), 7.50 (d, *J* = 8.4 Hz, 1H, Ar-H), 7.33 (t, *J* = 7.2 Hz, 1H, Ar-H), 4.06 (q, *J* = 5.4 Hz, 2H, N-CH_2_-), 1.23 (t, *J* = 7.2 Hz, 3H, -CH_3_); ^13^C NMR (150 MHz, DMSO-d_6_) *δ* (ppm) 159.0, 147.3, 141.1, 137.2, 129.6, 123.5, 114.6, 111.8, 11.90.

*1-(3-methylbut-2-en-1-yl)-1H-benzo[d][1,3]oxazine-2,4-dione* (**2d**)

Prepared according to the general method (A), isatoic anhydride (**1**) (4 g, 24.5 mmol) was added at room temperature to a mixture of NaH (883.2 mg, 36.8 mol, 60% *w*/*w* in oil) and 100 mL dry DMF, followed by the addition of prenyl bromide (4.3 mL, 36.8 mmol) at 0 °C. The product (**2d**) was obtained after the appropriate treatment in the form of a beige solid. Yield: 32% (680.9 mg). ^1^H NMR (600 MHz, DMSO-d_6_) *δ* (ppm) 8.01 (d, *J* = 7.8 Hz, 1H, Ar-H), 7.85 (t, *J* = 7.8 Hz, 1H, Ar-H), 7.33 (t, *J* = 7.8 Hz, 1H, Ar-H), 7.29 (d, *J* = 8.4 Hz, 1H, Ar-H), 5.19–5.17 (m, 1H, N-CH_2_-**CH**=), 4.64 (d, *J* = 6.0 Hz, 2H, N-**CH_2_**-CH=), 1.81 (s, 3H, -CH_3_), 1.70 (s, 3H, -CH_3_); ^13^C NMR (150 MHz, DMSO-d_6_) *δ* (ppm) 158.9, 147.6, 141.3, 137.14, 136.4, 129.6, 123.6, 118.2, 114.8, 111.8, 42.7, 25.3, 18.1.

*1-octyl-1H-benzo[d][1,3]oxazine-2,4-dione* (**2e**)

Prepared according to the general method (A), isatoic anhydride (**1**) (2 g, 12.3 mmol) was added at room temperature to a mixture of NaH (444 mg, 18.5 mol, 60% *w*/*w* in oil) and 100 mL dry DMF, followed by the addition of 1-bromooctane (3.2 mL, 18.5 mmol) at 0 °C. The product (**2e**) was obtained after the appropriate treatment in the form of a beige solid. Yield: 25% (813.8 mg). ^1^H NMR (600 MHz, CDCl_3_) *δ* (ppm) 8.16 (dd, *J* = 7.8, 1.8 Hz, 1H, Ar-H), 7.75 (ddd, *J* = 7.8, 7.2, 1.8 Hz, 1H, Ar-H), 7.28 (t, *J* = 7.8 Hz, 1H, Ar-H), 7.16 (d, *J* = 8.4 Hz, 1H, Ar-H), 4.04 (t, *J* = 7.8 Hz, 2H, N-CH_2_-), 1.75 (quint, *J* = 7.8 Hz, 2H, N-CH_2_-**CH_2_**-), 1.44–1.40 (m, 2H, -CH_2_-), 1.30–1.24 (m, 8H, 4×-CH_2_-), 0.87 (t, *J* = 7.2 Hz, 3H, -CH_3_). ^13^C NMR (150 MHz, CDCl_3_) *δ* (ppm) 158.7, 147.8, 141.5, 137.3, 131.1, 124.0, 114.0, 112.0, 77.2, 45.1, 31.9, 29.3, 28.9, 27.0, 27.8, 22.7, 14.2 [53].

*1-(3-phenylpropyl)-1H-benzo[d][1,3]oxazine-2,4-dione* (**2f**)

Prepared according to the general method (A), isatoic anhydride (**1**) (4 g, 24.5 mmol) was added at room temperature to a mixture of NaH (883.2 mg, 36.8 mol, 60% *w*/*w* in oil) and 100 mL dry DMF, followed by the addition of 1-bromo3-phenylpropane (5.6 mL, 36.8 mmol) at 0 °C. The product (**2f**) was obtained after the appropriate treatment in the form of a beige solid. Yield: 30% (2.1 g). ^1^H NMR (600 MHz, DMSO-d_6_) *δ* (ppm) 8.00 (d, *J* = 7.8 Hz, 1H, Ar-H), 7.82 (t, *J* = 8.4 Hz, 1H, Ar-H), 7.42 (d, *J* = 8.4 Hz, 1H, Ar-H), 7.32 (t, *J* = 7.8 Hz, 1H, Ar-H), 7.28–7.26 (m, 2H, Ar-H), 7.23 (d, *J* = 7.2 Hz, 2H, Ar-H), 7.17 (t, *J* = 7.8 Hz, 1H, Ar-H), 4.05 (t, *J* = 7.2 Hz, 2H, N-CH_2_-), 2.73 (t, *J* = 7.8 Hz, 2H, -CH_2_-Ar), 1.95 (t, *J* = 7.8 Hz, 2H, N-CH_2_-C**H_2_**-); ^13^C NMR (150 MHz, DMSO-d_6_) *δ* (ppm) 159.0, 147.5, 141.3, 141.2, 137.1, 129.6, 128.3, 128.2, 125.9, 123.5, 114.7, 111.8, 44.0, 32.0, 28.0 [54].

*(E)-1-(3-phenylprop-1-en-1-yl)-1H-benzo[d][1,3]oxazine-2,4-dione* (**2g**)

Prepared according to the general method (A), isatoic anhydride (**1**) (1 g, 6.1 mmol) was added at room temperature to a mixture of NaH (219.6 mg, 9.2 mol, 60% *w*/*w* in oil) and 25 mL dry DMF, followed by the addition of 3-bromo-1-phenyl-1-propene (3.2 mL, 9.2 mmol) at 0 °C. The product (**2g**) was obtained after the appropriate treatment in the form of a beige solid. Yield: 31% (520.0 mg). ^1^H NMR (600 MHz, DMSO-d_6_) *δ* (ppm) 8.15 (dd, *J* = 7.8, 1.2 Hz, 1H, Ar-H), 7.73 (ddd, *J* = 8.4, 7.8, 1.2 Hz, 1H, Ar-H), 7.34–7.33 (m, 2H, Ar-H), 7.30–7.27 (m, 3H, Ar-H), 7.26–7.23 (m, 2H, Ar-H), 6.66 (d, *J* = 15.6Hz, 1H, =CH-Ar), 6.24 (dt, *J* = 16.2, 5.4 Hz, 1H, N-CH_2_-C**H**=), 4.85 (dd, *J* = 6.0, 1.8 Hz, 2H, N-CH_2_-); ^13^C NMR (150 MHz, DMSO-d_6_) *δ* (ppm) 158.5, 147.9, 141.4, 137.4, 135.7, 134.2, 131.0, 128.7, 128.4, 126.6, 124.2, 121.2, 114.5, 111.9, 77.2, 46.9.

#### 2.1.2. General Method (Β): Synthesis of N-Substituted 4-Hydroxy-Quinolinones (**3a–3g**)

Sodium hydride (NaH) was suspended in dry DMF in a round-bottom flask and 1 eq. of N-substituted isatoic anhydride (**2**) and 5 eq. of dimethyl malonate were added at 0 °C. The mixture was refluxed at 80 °C under inert atmosphere for 2–2.5 h, with the reaction progress monitored by TLC. After completion of the reaction, the mixture was cooled in an ice-water bath, acidified with HCl (10% aqueous solution) and then extracted with Et_2_O; the organic layer was collected, dried over Na_2_SO_4_, and concentrated under reduced pressure. The desired quinolinone product (**3**) was obtained in a solid form and it was further purified through recrystallization from methanol/dichloromethane.

*Methyl 4-hydroxy-1-methyl-2-oxo-1,2-dihydroquinoline-3-carboxylate* (**3a**)

Prepared according to the general method (B), N-methyl-isatoic anhydride (**2a**) (444.3 mg, 2.5 mmol) was added to a mixture of NaH (120.5 mg, 5.0 mmol, 60% *w*/*w* in oil) in 20 mL of dry DMF, followed by the addition of dimethyl malonate (1.4 mL, 12.6 mmol) under cooling. After the work-up procedure, the product (**3a**) was obtained upon recrystallization from methanol as a white solid. Yield: 52%; M.p. 160–162 °C; ^1^H NMR (600 MHz, CDCl_3_): *δ* (ppm) 14.06 (s, 1H, -OH), 8.19 (d, *J* = 7.8Hz, 1H, Ar-H), 7.69 (t, *J* = 7.8Hz, 1H, Ar-H), 7.32 (d, *J* = 8.4 Hz, 1H, Ar-H), 7.26 (br, 1H, Ar-H), 4.03 (s, 3H, C=O-O-CH_3_), 3.65 (s, 3H, N-CH_3_); ^13^C NMR (75 MHz, CDCl_3_) *δ* (ppm) 173.9, 171.9, 159.5, 141.0, 134.5, 126.0, 122.0, 115.1, 114.7, 97.9, 53.2, 25.8 [52].

*Methyl 1-benzyl-4-hydroxy-2-oxo-1,2-dihydroquinoline-3-carboxylate* (**3b**)

Prepared according to the general method (B), N-benzyl-isatoic anhydride (**2b**) (2.38 g, 9.4 mmol) was added to a mixture of NaH (451.2 mg, 18.8 mmol, 60% *w*/*w* in oil) in 70 mL of dry DMF, followed by the addition of dimethyl malonate (5.4 mL, 47.0 mmol) under cooling. After the work-up procedure, the product (**3b**) was obtained upon recrystallization from methanol as a white solid. Yield: 46%; M.p. 151–152 °C; ^1^H NMR (600 MHz, CDCl_3_): *δ* (ppm) 8.20 (dd, *J* = 8.4, 1.8 Hz, 1H, Ar-H), 7.55 (td, *J* = 8.4, 1.8 Hz, 1H, Ar-H), 7.30–7.28 (m, 1H), 7.23–7.21 (m, 5H), 5.50 (s, 2H, N-CH_2_-Ar), 4.05 (s, 3H, C=O-O-CH_3_); ^13^C NMR (75 MHz, CDCl_3_) *δ* (ppm) 173.2, 171.8., 159.6, 141.2, 136.7, 134.6, 128.6, 128.6, 126.3, 126.1, 122.0, 115.1, 114.2, 97.8, 53.2, 45.1; HR-MS calcd for C_18_H_16_O_4_N (Μ + H)^+^: 310.1074, found: 310.1069 [55].

*Methyl 1-ethyl-4-hydroxy-2-oxo-1,2-dihydroquinoline-3-carboxylate* (**3c**)

Prepared according to the general method (B), N-ethyl-isatoic anhydride (**2c**) (1.5429 g, 8.1 mmol) was added to a mixture of NaH (388.8 mg, 16.2 mmol, 60% *w*/*w* in oil) in 60 mL of dry DMF, followed by the addition of dimethyl malonate (4.7 mL, 40.5 mmol) under cooling. The product (**3c**) was obtained after the work-up procedure in the form of a white solid. Yield: 40% (449.4 mg); M.p. 133–134 °C; ^1^H NMR (600 MHz, CDCl_3_): *δ* (ppm) 8.20 (dd, *J* = 8.4, 1.8 Hz, 1H, Ar-H), 7.68 (td, *J* = 8.4, 1.2 Hz, 1H, Ar-H), 7.33 (d, *J* = 8.4 Hz, 1H, Ar-H), 7.24–7.25 (m, 1H, Ar-H), 4.31 (q, *J* = 7.2 Hz, 2H, N-**CH_2_**-CH_3_), 4.04 (s, 3H, C=O-O-**CH_3_**), 1.33 (t, *J* = 7.2 Hz, 3H, N-**CH_2_**-CH_3_); ^13^C NMR (75 MHz, CDCl_3_) *δ* (ppm) 173.0, 171.9, 159.6, 141.0, 134.6, 126.0, 122.0, 115.2, 114.8, 98.0, 50.2, 25.9, 18.5; HR-MS calcd for C_13_H_14_O_4_N (Μ + H)^+^: *m*/*z*: 248.0917, found: 248.0916 [52].

*Methyl 4-hydroxy-1-(3-methylbut-2-en-1-yl)-2-oxo-1,2-dihydroquinoline-3-carboxylate* (**3d**)

Prepared according to the general method (B), N-prenyl-isatoic anhydride (**2d**) (680.9 mg, 2.9 mmol) was added to a mixture of NaH (139.2 mg, 5.8 mmol, 60% *w*/*w* in oil) in 22 mL of dry DMF, followed by the addition of dimethyl malonate (1.7 mL, 14.5 mmol) under cooling. After the work-up procedure, the product (**3d**) was obtained as a white solid. Yield: 32%; M.p. 119–121 °C; ^1^H NMR (600 MHz, CDCl_3_): *δ* (ppm) 8.19 (dd, *J* = 7.8, 1.2 Hz, 1H, Ar-H), 7.65 (td, *J* = 8.4, 1.2 Hz, 1H), 7.27–7.23 (m, 2H, Ar-H), 5.12 (t, *J* = 8.4 Hz, 1H, N-CH_2_-**CH**=), 4.87 (d, *J* = 5.4 Hz, 2H, N-**CH_2_**-CH=), 4.03 (s, 3H, C=O-O-CH_3_), 1.88 (s, 3H, -CH_3_), 1.71 (s, 3H, -CH_3_); ^13^C NMR (151 MHz, CDCl_3_): *δ* (ppm) 173.25, 171.84, 159.51, 140.95, 136.13, 134.49, 125.96, 121.96, 119.38, 115.13, 114.70, 97.88, 53.15, 40.89, 25.77, 18.46; HR-MS calcd for C_16_H_18_O_4_N (Μ + H)^+^: *m*/*z*: 288.1230, found: 288.1226.

*Methyl 4-hydroxy-1-octyl-2-oxo-1,2-dihydroquinoline-3-carboxylate* (**3e**)

Prepared according to the general method (B), N-octyl-isatoic anhydride (**2e**) (813.8 mg, 3.0 mmol) was added to a mixture of NaH (144.0 mg, 6.0 mmol, 60% *w*/*w* in oil) in 22 mL of dry DMF, followed by the addition of dimethyl malonate (1.7 mL, 15.0 mmol) under cooling. After the work-up procedure, the product (**3e**) was obtained as a white solid. Yield: 19%; M.p. 69–71 °C; ^1^H NMR (600 MHz, CDCl_3_): *δ* (ppm) 8.20 (dd, *J* = 7.8, 1.2 Hz, 1H, Ar-H), 7.68 (td, *J* = 8.4, 1.2 Hz, 1H, Ar-H), 7.30 (d, *J* = 8.4 Hz, 1H, Ar-H), 7.24 (t, *J* = 7.8 Hz, 1H, Ar-H), 4.20 (t, *J* = 7.8 Hz, 2H, N-CH_2_-), 4.03 (s, 3H, C=O-O-CH_3_), 1.71 (quint, *J* = 7.8 Hz, 2H, N-CH_2_-**CH_2_**-), 1.45 (quint, *J* = 7.8 Hz, 2H, N-CH_2_-CH_2_-**CH_2_**-), 1.36 (quint, *J* = 7.2 Hz, 2H, N-(CH_2_)_3_-**CH_2_**-), 1.33–1.25 (m, 6H, N-(CH_2_)_4_-**(CH_2_)_3_**-CH_3_), 0.88 (t, *J* = 6.6 Hz, 3H, -CH_3_); ^13^C NMR (150 MHz, CDCl3): δ (ppm) 173.25, 171.73, 159.51, 140.80, 134.54, 126.07, 121.89, 115.11, 114.33, 97.85, 53.15, 42.63, 31.94, 29.47, 29.38, 27.63, 27.21, 22.77, 14.23; HR-MS calcd for C_19_H_26_O_4_N (Μ + H)^+^: *m*/*z*: 332.1856, found: 332.1850.

*Methyl 4-hydroxy-2-oxo-1-(3-phenylpropyl)-1,2-dihydroquinoline-3-carboxylate* (**3f**)

Prepared according to the general method (B), N-phenylpropyl-isatoic anhydride (**2f**) (2.1 g, 7.5 mmol) was added to a mixture of NaH (360.0 mg, 15.0 mmol, 60% *w*/*w* in oil) in 56 mL of dry DMF, followed by the addition of dimethyl malonate (4.3 mL, 37.5 mmol) under cooling. After the work-up procedure, the product (**3f**) was obtained as a white solid. Yield: 56%; M.p. 101–104 °C; ^1^H NMR (600 MHz, CDCl_3_): *δ* (ppm) 8.18 (dd, *J* = 7.8, 1.2 Hz, 1H, Ar-H), 7.59 (td, *J* = 8.4, 1.2 Hz, 1H, Ar-H), 7.30 (t, *J* = 7.2 Hz, 2H, Ar-H), 7.24–7.20 (m, 4H, Ar-H), 7.06 (d, *J* = 8.4 Hz, 1H, Ar-H), 4.24 (t, *J* = 7.8 Hz, 2H, N-CH_2_-), 4.04 (s, 3H, C=O-O-CH_3_), 2.80 (t, *J* = 7.8 Hz, 2H, -CH_2_-Ar), 2.05 (quint, *J* = 7.8 Hz, 2H, N-CH_2_-**CH_2_**-CH_2_-Ar); ^13^C NMR (150 MHz, CDCl_3_): δ (ppm) 173.19, 171.77, 159.53, 141.19, 140.62, 134.55, 128.59, 128.52, 126.23, 126.05, 121.91, 115.08, 114.14, 97.78, 53.16, 42.00, 33.38, 28.89; HR-MS calcd for C_20_H_20_O_4_N (Μ + H)^+^: *m*/*z*: 338.1387, found: 338.1382.

*Methyl 1-cinnamyl-4-hydroxy-2-oxo-1,2-dihydroquinoline-3-carboxylate* (**3g**)

Prepared according to the general method (B), N-cinnamyl-isatoic anhydride (**2g**) (520.0 mg, 1.9 mmol) was added to a mixture of NaH (91.2 mg, 3.8 mmol, 60% *w*/*w* in oil) in 14 mL of dry DMF, followed by the addition of dimethyl malonate (1.1 mL, 9.3 mmol) under cooling. After the work-up procedure, the product (**3g**) was obtained as a white solid. Yield: 41%; M.p. 177–178 °C; ^1^H NMR (600 MHz, CDCl_3_): *δ* (ppm) 14.18 (brs, 1H, -OH) 8.22 (dd, *J* = 7.8, 1.2 Hz, 1H, Ar-H), 7.66 (td, *J* = 9, 1.8 Hz, 1H, Ar-H), 7.38 (d, *J* = 9 Hz, 1H, Ar-H), 7.32–7.31 (m, 2H, Ar-H), 7.28–7.25 (m, 3H, Ar-H), 7.20 (t, *J* = 7.8 Hz, 1H, Ar-H), 6.55 (d, *J* = 16.2 Hz, 1H, N-CH_2_-**CH**=CH-Ar), 6.29 (dt, *J* = 16.2, 6 Hz, 1H, N-CH_2_-CH=**CH**-Ar), 5.06 (d, *J* = 4.8 Hz, 2H, N-CH_2_-), 4.05 (s, 3H, C=O-O-CH_3_); ^13^C NMR (150 MHz, CDCl_3_): *δ* (ppm) 173.20, 172.11, 159.54, 140.91, 136.43, 134.67, 132.77, 128.65, 127.90, 126.53, 126.04, 123.55, 122.22, 115.19, 114.84, 97.82, 53.22, 44.31; HR-MS calcd for C_20_H_18_O_4_N (Μ + H)^+^: *m*/*z*: 336.1230, found: 336.1230.

#### 2.1.3. General Method (C): Synthesis of Carboxamides (**4a–4g**)

To a stirred solution of 1 eq of N-substituted-4-hydroxy-quinolinone (**3**), dissolved in toluene, 2 eq of propargylamine was added. The reaction mixture was refluxed at 110 °C under inert atmosphere for 2–2.5 h. The completion of the reaction was monitored by TLC. At the end of the reaction, the mixture was cooled to 0 °C (ice-water bath) and the precipitate formed was filtered and washed with Et_2_O. The product (**4**) was obtained in solid form and used without purification in the next step.

*4-hydroxy-1-methyl-2-oxo-N-(prop-2-yn-1-yl)-1,2-dihydroquinoline-3-carboxamide* (**4a**)

Prepared according to the general method (C), N-methyl-quinolinone (**3a**) (508 mg, 2.2 mmol) was dissolved in 12 mL toluene, followed by the addition of propargylamine (281 μL, 4.4 mmol). After the work-up procedure, the product (**4a**) was obtained as an orange-brown solid. Yield: 82% (463.1 mg); M.p. 195.0–198.0 °C; ^1^H NMR (600 MHz, CDCl_3_): *δ* (ppm) 16.52 (s, 1H, -OH), 10.51 (brs, 1H, -NH), 8.21 (dd, *J* = 8.4, 1.2 Hz, 1H, Ar-H), 7.70 (ddd, *J* = 8.4, 7.8, 1.2 Hz, 1H, Ar-H), 7.36 (d, *J* = 8.4 Hz, 1H, Ar-H), 7.31 (t, *J* = 7.8 Hz, 1H, Ar-H), 4.24 (dd, *J* = 5.4, 2.4 Hz, 2H, NH-C**H_2_**-), 3.68 (s, 3H, N-CH_3_), 2.27 (br, 1H, -C≡CH); ^13^C NMR (75 MHz, CDCl_3_) *δ* (ppm) 171.8, 171.0, 162.7, 140.1, 134.1, 125.7, 122.6, 116.1, 114.4, 97.0, 79.2, 71.7, 29.3, 28.8; HR-MS calcd for C_14_H_13_O_3_N_2_ (Μ + H)^+^: *m*/*z*: 257.0926, found: 257.0925.

*1-benzyl-4-hydroxy-2-oxo-N-(prop-2-yn-1-yl)-1,2-dihydroquinoline-3-carboxamide* (**4b**)

Prepared according to the general method (C), N-benzyl-quinolinone (**3b**) (521 mg, 1.7 mmol) was dissolved in 12 mL toluene, followed by the addition of propargylamine (218 μL, 3.4 mmol). After the work-up procedure, the product (**4b**) was obtained as an orange-brown solid. Yield: 71% (402.0 mg); M.p. 178–182 °C; ^1^H NMR (600 MHz, CDCl_3_): *δ* (ppm) 16.67 (s, 1H, -OH), 10.53 (brs, 1H, -NH), 8.24 (d, *J* = 7.8 Hz, 1H, Ar-H), 7.56 (ddd, *J* = 8.4, 7.8, 1.2 Hz, 1H, Ar-H), 7.32–7.30 (m, 2H, Ar-H), 7.28–7.25 (m, 3H, Ar-H), 7.19–7.18 (m, 2H, Ar-H), 5.52 (s, 2H, N-CH_2_-Ar), 4.25 (dd, *J* = 5.4, 2.4 Hz, 2H, -NH-C**H_2_**-), 2.27 (br, 1H, -C≡CH); ^13^C NMR (75 MHz, CDCl_3_): *δ* (ppm) 172.2, 171.0, 162.8, 139.7, 136.1, 134.0, 129.1, 127.6, 126.2, 125.7, 122.7, 116.4, 115.2, 96.8, 71.7, 45.9, 28.8; HR-MS calcd for C_20_H_17_O_3_N_2_ (Μ + H)^+^: *m*/*z*: 333.1239, found: 333.1234.

*1-ethyl-4-hydroxy-2-oxo-N-(prop-2-yn-1-yl)-1,2-dihydroquinoline-3-carboxamide* (**4c**)

Prepared according to the general method (C), N-ethyl-quinolinone (**3c**) (402.4 mg, 1.6 mmol) was dissolved in 10 mL toluene, followed by the addition of propargylamine (215 μL, 3.2 mmol). After the work-up procedure, the product (**4c**) was obtained as an orange-brown solid. Yield: 77% (338.7 mg); M.p. 190–195 °C; ^1^H NMR (600 MHz, CDCl_3_): *δ* (ppm) 16.48 (s, 1H, -OH), 10.59 (brs, 1H, -NH), 8.24 (dd, *J* = 7.8, 1.2 Hz, 1H, Ar-H), 7.69 (ddd, *J* = 9.0, 8.1, 1.8 Hz, 1H, Ar-H), 7.38 (d, *J* = 8.4 Hz, 1H, Ar-H), 7.30 (t, *J* = 7.8 Hz, 1H, Ar-H), 4.33 (q, *J* = 7.2 Hz, 2H, N-CH_2_), 4.24 (dd, *J* = 5.4, 2.4 Hz, 2H, NH-C**H_2_**), 2.27 (br, 1H, -C≡CH), 1.36 (t, *J* = 7.2 Hz, 3H, -CH_3_); ^13^C NMR (150 MHz, CDCl_3_): *δ* (ppm) 171.7, 171.0, 162.3, 139.1, 134.0, 125.9, 122.4, 116.4, 114.3, 96.9, 79.3, 71.7, 37.4, 28.8, 13.0; HR-MS calcd for C_15_H_15_O_3_N_2_ (Μ + H)^+^: *m*/*z*: 271.1086, found: 271.1076.

*4-hydroxy-1-(3-methylbut-2-en-1-yl)-2-oxo-N-(prop-2-yn-1-yl)-1,2-dihydroquinoline-3-carboxamide* (**4d**)

Prepared according to the general method (C), N-prenyl-quinolinone (**3d**) (228.8 mg, 0.8 mmol) was dissolved in 8 mL toluene, followed by the addition of propargylamine (102.5 μL, 1.6 mmol). After the work-up procedure, the product (**4d**) was obtained as an orange-brown solid. Yield: 55% (136.6 mg); M.p. 150.0–153.0 °C; ^1^H NMR (600 MHz, CDCl_3_): *δ* (ppm) 16.51 (s, 1H, -OH), 10.57 (brs, 1H, -NH), 8.22 (dd, *J* = 7.8, 1.2 Hz, 1H, Ar-H), 7.66 (ddd, *J* = 8.4, 7.8, 1.2 Hz, 1H, Ar-H), 7.31–7.28 (m, 2H, Ar-H), 5.10 (t, *J* = 6.0Hz, 1H, -CH=C), 4.90 (d, *J* = 4.2 Hz, 2H, N-CH_2_-), 4.23 (dd, *J* = 5.4, 2.4 Hz, 2H, NH-C**H_2_**-), 2.26 (br, 1H, -C≡CH), 1.89 (s, 3H, -CH_3_), 1.74 (s, 3H, -CH_3_); ^13^C NMR (75 MHz, CDCl_3_): *δ* (ppm) 171.8, 171.0, 162.4, 139.5, 136.4, 133.9, 125.7, 122.4, 119.3, 116.3, 114.8, 96.9, 79.3, 71.6, 40.9, 28.7, 25.7, 18.5; HR-MS calcd for C_18_H_19_O_3_N_2_ (Μ + H)^+^: *m*/*z*: 311.1390, found: 311.1391.

*4-hydroxy-1-octyl-2-oxo-N-(prop-2-yn-1-yl)-1,2-dihydroquinoline-3-carboxamide* (**4e**)

Prepared according to the general method (C), N-octyl-quinolinone (**3e**) (180.0 mg, 0.5 mmol) was dissolved in 7 mL toluene, followed by the addition of propargylamine (69.0 μL, 1.1 mmol). After the work-up procedure, the product (**4e**) was obtained as an orange-brown solid. Yield: 10% (14.3 mg); M.p. 47.0–49.0 °C; ^1^H NMR (600 MHz, CDCl_3_): *δ* (ppm) 16.49 (s, 1H, -OH), 10.59 (brs, 1H, -NH), 8.23 (dd, *J* = 7.8, 1.2 Hz, 1H, Ar-H), 7.69 (ddd, *J* = 9.0, 8.1, 1.8 Hz, 1H, Ar-H), 7.35 (d, *J* = 9.0 Hz, 1H, Ar-H), 7.30 (t, *J* = 7.8 Hz, 1H, Ar-H), 4.24–4.23 (m, 4H, N-CH_2_, NH-C**H_2_**-), 2.27 (br, 1H, -C≡CH), 1.72 (quint, *J* = 7.8 Hz, 2H, N-CH_2_-C**H_2_**-), 1.46 (quint, *J* = 7.2 Hz, 2H, N-(CH_2_)_2_-C**H_2_**-), 1.38 (quint, *J* = 7.2Hz, 2H, N-(CH_2_)_3_-C**H_2_**-), 1.29 (m, 6H, 3×-CH_2_-), 0.89 (t, *J* = 7.2 Hz, 3H, -CH_3_); ^13^C NMR (75 MHz, DMSO-d_6_): *δ* (ppm) 171.0, 170.4, 161.3, 139.0, 134.5, 124.8, 122.5, 115.3, 115.1, 95.9, 80.1, 73.8, 41.6, 34.1, 31.2, 28.7, 28.2, 27.2, 26.3, 22.1, 14.0; HR-MS calcd for C_21_H_27_O_3_N_2_ (Μ + H)^+^: *m*/*z*: 355.2022, found: 355.2014.

*4-hydroxy-2-oxo-1-(3-phenylpropyl)-N-(prop-2-yn-1-yl)-1,2-dihydroquinoline-3-carboxamide* (**4f**)

Prepared according to the general method (C), N-phenylpropyl-quinolinone (**3f**) (700.0 mg, 2.1 mmol) was dissolved in 15 mL toluene, followed by the addition of propargylamine (269.0 μL, 4.2 mmol). After the work-up procedure, the product (**4f**) was obtained as an orange-brown solid. Yield: 70% (521.8 mg); M.p. 84.0–86.0 °C; ^1^H NMR (600 MHz, CDCl_3_): *δ* (ppm) 16.49 (s, 1H, -OH), 10.57 (brs, 1H, -NH), 8.21 (dd, *J* = 7.8, 1.2 Hz, 1H, Ar-H), 7.60 (ddd, *J* = 8.4, 7.8, 1.2 Hz, 1H, Ar-H), 7.33–7.30 (m, 2H, Ar-H), 7.28–7.22 (m, 4H, Ar-H), 7.10 (d, *J* = 8.4 Hz, 1H, Ar-H), 4.28–4.23 (m, 4H, N-CH_2_, NH-C**H_2_**-), 2.80 (t, *J* = 7.2 Hz, 2H, -CH_2_-Ar), 2.28 (br, 1H, -C≡CH), 2.06 (quint, *J* = 7.8 Hz, 2H, N-CH_2_-C**H_2_**-); ^13^C NMR (75 MHz, CDCl_3_): *δ* (ppm) 171.7, 171.0, 162.4, 141.0, 139.2, 134.0, 128.6, 128.5, 126.3, 125.8, 122.4, 116.3, 114.2, 96.8, 79.2, 71.7, 41.8, 33.3, 29.0, 28.7; HR-MS calcd for C_22_H_21_O_3_N_2_ (Μ + H)^+^: *m*/*z*: 361.1552, found: 361.1546.

*1-cinnamyl-4-hydroxy-2-oxo-N-(prop-2-yn-1-yl)-1,2-dihydroquinoline-3-carboxamide* (**4g**)

Prepared according to the general method (C), N-cinnamyl-quinolinone (**3g**) (175.0 mg, 0.5 mmol) was dissolved in 7 mL toluene, followed by the addition of propargylamine (76.0 μL, 1.2 mmol). After the work-up procedure, the product (**4g**) was obtained as an orange-brown solid. Yield: 48% (90.0 mg); M.p. 93.5–97.0 °C; ^1^H NMR (600 MHz, CDCl_3_): *δ* (ppm) 16.61 (s, 1H, -OH), 10.53 (brs, 1H, -NH), 8.25 (dd, *J* = 8.4, 1.2 Hz, 1H, Ar-H), 7.67 (ddd, *J* = 8.4, 7.8, 1.2 Hz, 1H, Ar-H), 7.41 (d, *J* = 9.0Hz, 1H, Ar-H), 7.33–7.31 (m, 3H, Ar-H), 7.30–7.27 (m, 2H, Ar-H), 7.22 (t, *J* = 7.2 Hz, 1H, Ar-H), 6.49 (d, *J* = 16.2 Hz, 1H, -CH-Ar), 6.30 (dt, *J* = 16.2, 5.4 Hz, 1H, -C**H**=CH-Ar), 5.08 (d, *J* = 4.2 Hz, 2H, N-CH_2_-), 4.25 (dd, *J* = 4.8, 2.4 Hz, 2H, NH-C**H_2_**-), 2.27 (br, 1H, -C≡CH); ^13^C NMR (150 MHz, CDCl_3_): *δ* (ppm) 172.0, 170.9, 162.3, 139.4, 136.1, 133.9, 132.4, 128.5, 127.9, 126.4, 125.7, 123.0, 122.5, 116.2, 114.8, 96.7, 79.0, 77.5, 77.0, 76.6, 71.6, 44.1, 28.7. HR-MS calcd for C_22_H_19_O_3_N_2_ (Μ + H)^+^: *m*/*z*: 359.1396, found: 359.1390.

#### 2.1.4. General Method (D): Synthesis of Quinolinone–triazoles (**5a–5j**)

In a quartz μW-reaction tube, equimolar quantities of sodium azide (NaN_3_), triethylamine (Et_3_N), the appropriate alkyl bromide and carboxamide (**4**), along with the appropriate amount of copper sulfate (CuSO_4_), and sodium ascorbate as catalyst, were added to a mixture t-BuOH:H_2_O in a ratio 1:1. The reaction mixture was stirred under microwave radiation at 80 °C, 100 W for 10 min. The completion of the reaction was monitored by TLC. At the end of the reaction, the mixture was cooled to 0 ^°^C (ice-water bath) and the precipitate formed was filtered and washed with water. The precipitate underwent a silica gel column chromatography in order to obtain the product (**5**) in high purity as a white solid.

*N-((1-benzyl-1H-1,2,3-triazol-4-yl)methyl)-4-hydroxy-1-methyl-2-oxo-1,2-dihydroquinoline-3-carboxamide* (**5a**)

Prepared according to the general method (D), in a quartz vessel, 1.75 mmol (113.8 mg) NaN_3_, 1.75 mmol (244.0 μL) Et_3_N, 1.75 mmol (208.0 μL) benzyl bromide, 0.35 mmol (69.3mg) sodium ascorbate, 0.35 mmol (55.9 mg) CuSO_4_ and 1.75 mmol (449.0 mg) of carboxamide (**4a**) were dissolved in about 7mL of 1:1 t-BuOH/H_2_O solution. After the work-up procedure, the product (**5a**) was subjected to further purification via column chromatography, using PE/EtOAc 50:50 as eluent and was obtained as white solid. Yield: 40% (272.2 mg); M.p. 146.2–146.7 °C; ^1^H NMR (600 MHz, CDCl_3_): *δ* (ppm), 16.72 (s, 1H, 4-OH), 10.73 (brs, 1H,N-H), 8.19 (dd, *J* = 7.8, 1.2 Hz, 1H, Ar-H), 7.68 (ddd, *J* = 8.4, 7.8, 1.2 Hz, 1H, Ar-H), 7.52 (s, 1H, N-**CH**=C-), 7.38–7.34 (m, 4H, Ar-H), 7.31–7.26 (m, 3H, Ar-H), 5.51 (s, 2H, N-**CH_2_**-Ar), 4.74 (br, 2H, NH-**CH_2_-**), 3.65 (s, 3H, N-**CH_3_**); ^13^C NMR (75 MHz, CDCl_3_): *δ* (ppm) 171.8, 171.2, 162.6, 145.1, 140.0, 134.7, 133.9, 129.2, 128.8, 128.2, 125.5, 122.5, 122.2, 116.1, 114.3, 96.9, 54.3, 34.9, 29.2; IR 3118.33 cm^−1^ ($\tilde{\nu }$, aromatic C-H), 2935.13 cm^−1^ ($\tilde{\nu }$, aliphatic C-H), 1631.43 cm^−1^ ($\tilde{\nu }$, 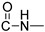


), 1590.99 cm^−1^ ($\tilde{\nu }$, N=N), 1563.99 cm^−1^ ($\tilde{\nu }$, N-H), 1340.28 cm^−1^ ($\tilde{\nu }$, C-N), 711.604 cm^−1^ ($\tilde{\delta }$, aromatic monosubstitution); HR-MS calcd for C_21_H_18_O_3_N_5_ (Μ − H)^−^: *m*/*z*: 388.1410, found: 388.1407.

*1-benzyl-N-((1-benzyl-1H-1,2,3-triazol-4-yl)methyl)-4-hydroxy-2-oxo-1,2-dihydroquinoline-3-carboxamide* (**5b**)

Prepared according to the general method (D), in a quartz vessel, 1.20 mmol (401.0 mg) NaN_3_, 1.20 mmol (167.0 μL) Et_3_N, 1.20 mmol (143.0 μL) benzyl bromide, 0.24 mmol (47.5mg) sodium ascorbate, 0.24 mmol (38.3 mg) CuSO_4_ and 1.20 mmol (401.0 mg) of carboxamide (**4b**) were dissolved in about 7mL of 1:1 t-BuOH/H_2_O solution. After the work-up procedure, the product (**5b**) was subjected to further purification via column chromatography, using PE/EtOAc 50:50 as eluent and was obtained as white solid. Yield: 18% (100.2 mg); M.p. 187.6–188.4 °C; ^1^H NMR (600 MHz, CDCl_3_): *δ* (ppm), 16.91 (s, 1H, 4-OH), 10.69 (brs, 1H, -NH), 8.22 (dd, *J* = 7.8, 0.6 Hz, 1H, Ar-H), 7.54 (ddd, *J* = 8.4, 7.8, 1.2 Hz, 1H, Ar-H), 7.50 (s, 1H, N-**CH**=C-), 7.38–7.34 (m, 3H, Ar-H), 7.31–7.27 (m, 4H, Ar-H), 7.25–7.23 (m, 3H, Ar-H), 7.16 (m, 2H, Ar-H), 5.51 (s, 4H, 2x N-**CH**-Ar), 4.73 (br, 2H, NH-**CH_2_**-); ^13^C NMR (75 MHz, CDCl_3_): *δ* (ppm) 172.3, 171.3, 162.9, 145.0, 139.7, 136.2, 134.7, 134.0, 129.2, 129.0, 128.9, 128.2, 127.5, 126.4, 125.7, 122.6, 122.2, 166.5, 115.2, 96.8, 54.3, 45.8, 35.0; IR 3126.04 cm^−1^ ($\tilde{\nu }$ aromatic C-H), 1660.77 cm^−1^ ($\tilde{\nu }$, 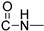


), 1589.06 cm^−1^ ($\tilde{\nu }$, N=N), 1535.06 cm^−1^ ($\tilde{\nu }$, N-H), 1328.71 cm^−1^ ($\tilde{\nu }$, C-N), 725.104 cm^−1^ ($\tilde{\delta }$, aromatic monosubstitution); HR-MS calcd for C_27_H_24_O_3_N_5_ (Μ + H)^+^: *m*/*z*: 466.1879, found: 466.1873.

*N-((1-benzyl-1H-1,2,3-triazol-4-yl)methyl)-1-ethyl-4-hydroxy-2-oxo-1,2-dihydroquinoline-3-carboxamide* (**5c**)

Prepared according to the general method (D), in a quartz vessel, 0.60 mmol (39.1 mg) NaN_3_, 0.60 mmol (84.0 μL) Et_3_N, 0.60 mmol (71.0 μL) benzyl bromide, 0.12 mmol (23.8 mg) sodium ascorbate, 0.12 mmol (19.2 mg) CuSO_4_ and 0.60 mmol (162.0 mg) of carboxamide (**4c**) were dissolved in about 7mL of 1:1 t-BuOH/H_2_O solution. After the work-up procedure, the product (**5c**) was subjected to further purification via column chromatography, using PE/EtOAc 50:50 as eluent and was obtained as white solid. Yield: 32% (77.5 mg); M.p. 144.0–144.5 °C; ^1^H NMR (600 MHz, CDCl_3_): *δ* (ppm) 16.67 (s, 1H, 4-OH), 10.76 (brs, 1H, -NH), 8.21 (dd, *J* = 7.8, 1.2 Hz, 1H, Ar-H), 7.68 (ddd, *J* = 7.2, 1.8 Hz, 1H, Ar-H), 7.52 (s, 1H, N-**CH**=C-), 7.38–7.34 (m, 4H, Ar-H), 7.30–7.26 (m, 3H, Ar-H), 5.51 (s, 2H, N-**CH_2_**-Ar), 4.73 (br, 2H, NH-**CH_2_-**), 4.29 (q, *J* = 7.2 Hz, 2H, -**CH_2_**-CH_3_), 1.32 (t, *J* = 6.6 Hz, 3H, -CH_2_-**CH_3_**); ^13^C NMR (75 MHz, CDCl_3_): *δ* (ppm) 171.7, 171.3, 162.2, 145.1, 139.1, 134.7, 133.9, 129.2, 128.8, 128.2, 125.8, 122.3, 122.2, 116.4, 114.2, 97.0, 54.3, 37.3, 34.9, 13.0; IR 3442.31 cm^−1^ ($\tilde{\nu }$, R-OH), 3168.47 cm^−1^ ($\tilde{\nu }$, aromatic C-H), 2980.45 cm^−1^ ($\tilde{\nu }$, aliphatic C-H), 1644.02 cm^−1^ ($\tilde{\nu }$, 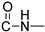


), 1580.88 cm^−1^ ($\tilde{\nu }$, N=N), 1536.99 cm^−1^ ($\tilde{\nu }$, N-H), 1327.75 cm^−1^ ($\tilde{\nu }$, C-N), 767.53 cm^−1^ ($\tilde{\delta }$, aromatic monosubstitution); HR-MS calcd for C_22_H_22_O_3_N_5_ (Μ + H)^+^: *m*/*z*: 404.1723, found: 404.1716.

*1-ethyl-4-hydroxy-2-oxo-N-((1-(3-phenylpropyl)-1H-1,2,3-triazol-4-yl)methyl)-1,2-dihydroquinoline-3-carboxamide* (**5d**)

Prepared according to the general method (D), in a quartz vessel, 1.23 mmol (80.0 mg) NaN_3_, 1.23 mmol (171.6 μL) Et_3_N, 1.23 mmol (187.2 μL) 1-bromo-3-phenylpropane, 0.25 mmol (49.5 mg) sodium ascorbate, 0.25 mmol (39.9 mg) CuSO_4_ and 1.23 mmol (333.0 mg) of carboxamide (**4c**) were dissolved in about 7mL of 1:1 t-BuOH/H_2_O solution. After the work-up procedure, the product (**5d**) was subjected to further purification via column chromatography, using PE/EtOAc 60:40 as eluent and was obtained as white solid. Yield: 48% (254.7 mg); M.p. 84.0–86.0 °C; ^1^H NMR (600 MHz, CDCl_3_): *δ* (ppm), 16.75 (s, 1H, 4-OH), 10.78 (brs, 1H, -NH), 8.23 (d, *J* = 7.8 Hz, 1H, Ar-H), 7.68 (t, *J* = 8.4 Hz, 1H, Ar-H), 7.58 (brs, 1H, N-**CH**=C-), 7.37 (d, *J* = 8.4 Hz, 1H, Ar-H), 7.30–7.28 (m, 3H, Ar-H), 7.21–7.17 (m, 3H, Ar-H), 4.75 (s, 2H, NH-**CH_2_**-), 4.32 (m, 4H, 2x N-**CH_2_**-), 2.66 (t, *J =* 6.6 Hz, 2H, N-CH_2_-CH_2_-**CH_2_**-Ar), 2.26 (t, *J* = 5.4 Hz, 2H, N-CH_2_-**CH_2_**-CH_2_-Ar), 1.33 (t, *J* = 6.6 Hz, N-CH_2_-**CH_3_**); ^13^C NMR (75 MHz, CDCl_3_): *δ* (ppm) 171.7, 171.3, 162.2, 140.2, 139.1, 133.9, 128.7, 128.5, 126.4, 125.8, 122.3, 116.4, 114.2, 96.9, 49.6, 37.3, 34.9, 32.6, 31.7, 12.9; IR 3145.33 cm^−1^ ($\tilde{\nu }$, aromatic C-H), 2935.13 cm^−1^ ($\tilde{\nu }$, aliphatic C-H), 1637.27 cm^−1^ ($\tilde{\nu }$, 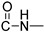


), 1587.13 cm^−1^ ($\tilde{\nu }$, N=N), 1554.34 cm^−1^ ($\tilde{\nu }$, N-H), 1334.50 cm^−1^ ($\tilde{\nu }$, C-N), 748.245 cm^−1^ ($\tilde{\delta }$, aromatic monosubstitution); HR-MS calcd for C_24_H_26_O_3_N_5_ (Μ + H)^+^: *m*/*z*: 432.2036, found: 432.2025.

*(E)-N-((1-cinnamyl-1H-1,2,3-triazol-4-yl)methyl)-1-ethyl-4-hydroxy-2-oxo-1,2-dihydroquinoline-3-carboxamide* (**5e**)

Prepared according to the general method (D), in a quartz vessel, 0.30 mmol (19.5 mg) NaN_3_, 0.30 mmol (41.9 μL) Et_3_N, 0.30 mmol (59.1 mg) cinnamyl bromide, 0.06 mmol (11.9 mg) sodium ascorbate, 0.06 mmol (9.6 mg) CuSO_4_ and 0.30 mmol (67.5 mg) of carboxamide (**4c**) were dissolved in about 7mL of 1:1 t-BuOH/H_2_O solution. After the work-up procedure, the product (**5e**) was subjected to further purification via column chromatography, using PE/EtOAc 50:50 as eluent and was obtained as white solid. Yield: 52% (70.0 mg); M.p. 130.0–136.0 °C; ^1^H NMR (600 MHz, CDCl_3_): *δ* (ppm), 16.69 (s, 1H, 4-OH), 10.79 (brs, 1H, -NH), 8.22 (dd, *J* = 7.8, 1.2 Hz, 1H, Ar-H), 7.68 (ddd, *J* = 8.4, 1.8 Hz, 1H, Ar-H), 7.65 (s, 1H, N-**CH**=C-), 7.39–7.35 (m, 3H, Ar-H), 7.34–7.31 (m, 2H, Ar-H), 7.30–7.28 (m, 2H, Ar-H), 6.67 (d, *J* = 15.6 Hz, 1H, N-CH_2_-CH=**CH**-Ar), 6.34 (dt, *J* = 15.6, 6.6 Hz, 1H, N-CH_2_-**CH**=CH-Ar), 5.12 (br, 2H, NH-**CH_2_-**), 4.78 (d, *J* = 5.4 Hz, 2H, N-**CH_2_**-CH=CH-Ar), 4.30 (q, *J* = 7.2 Hz, 2H, N-**CH_2_**-CH_3_), 1.32 (t, *J* = 7.2 Hz, 3H, N-CH_2_-**CH_3_**); ^13^C NMR (75 MHz, CDCl_3_): *δ* (ppm) 171.5, 171.4, 162.0, 144.4, 139.0, 136.1, 135.2, 133.9, 128.7, 128.6, 126.8, 125.7, 122.8, 122.2, 121.1, 116.2, 96.8, 53.0, 37.2, 34.3, 12.8; IR 3083.62 cm^−1^ ($\tilde{\nu }$, aromatic C-H), 2983.34 cm^−1^ ($\tilde{\nu }$, aliphatic C-H), 1648.91 cm^−1^ ($\tilde{\nu }$, 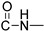


), 1581.34 cm^−1^ ($\tilde{\nu }$, N=N), 1536.99 cm^−1^ ($\tilde{\nu }$, N-H), 1326.79 cm^−1^ ($\tilde{\nu }$, C-N), 763.673 cm^−1^ ($\tilde{\delta }$, aromatic monosubstitution); HR-MS calcd for C_24_H_24_O_3_N_5_ (Μ + H)^+^: *m*/*z*: 430.1879, found: 430.1871.

*N-((1-benzyl-1H-1,2,3-triazol-4-yl)methyl)-4-hydroxy-2-oxo-1-(3-phenylpropyl)-1,2-dihydroquinoline-3-carboxamide* (**5f**)

Prepared according to the general method (D), in a quartz vessel, 0.55 mmol (35.8 mg) NaN_3_, 0.55 mmol (76.8 μL) Et_3_N, 0.55 mmol (65.3.0 μL) benzyl bromide, 0.11 mmol (21.8 mg) sodium ascorbate, 0.11 mmol (17.6 mg) CuSO_4_ and 0.55 mmol (200.0 mg) of carboxamide (**4f**) were dissolved in about 7mL of 1:1 t-BuOH/H_2_O solution. After the work-up procedure, the product (**5f**) was subjected to further purification via column chromatography, using PE/EtOAc 60:40 as eluent and was obtained as white solid. Yield: 45% (122.2 mg); M.p. 101.0–104.5 °C; ^1^HNMR (600 MHz, CDCl_3_): *δ* (ppm), 16.71 (s, 1H, 4-OH), 10.74 (brs, 1H, -NH), 8.19 (d, *J* = 7.8 Hz, 1H, Ar-H), 7.58 (t, *J* = 8.4 Hz, 1H, Ar-H), 7.51 (brs, 1H, N-**CH**=C-), 7.38–7.33 (m, 3H, Ar-H), 7.32–7.27 (m, 4H, Ar-H), 7.24–7.21 (m, 4H, Ar-H), 7.08 (d, *J* = 8.4 Hz, 1H, Ar-H), 5.51 (s, 2H, N-**CH_2_**-Ar), 4.73 (s, 2H, NH-**CH_2_**), 4.23 (t, *J* = 6 Hz**,** 2H, N-**CH_2_**-), 2.66 (t, *J* = 6.6 Hz, 2H, N-CH_2_-CH_2_-**CH_2_**-Ar), 2.78 (t, *J* = 7.2 Hz, 2H, -**CH_2_**-Ar), 2.03 (m, 2H, N-CH_2_-**CH_2_**-CH_2_-Ar); ^13^C NMR (75 MHz, CDCl_3_): *δ* (ppm) 171.7, 171.4, 141.0, 139.2, 134.3, 133.9, 129.3, 129.0, 128.6, 128.5, 128.3, 126.3, 125.7, 122.3, 116.3, 114.2, 96.8, 54.7, 41.8, 34.6, 33.2, 29.8, 28.9; IR 3122.19 cm^−1^ ($\tilde{\nu }$, aromatic C-H), 2973.7 cm^−1^ ($\tilde{\nu }$, aliphatic C-H), 1619.91 cm^−1^ ($\tilde{\nu }$, 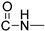


), 1563.99 cm^−1^ ($\tilde{\nu }$, N-H), 1328.71 cm^−1^ ($\tilde{\nu }$, C-N), 759.816 cm^−1^ ($\tilde{\delta }$, aromatic monosubstitution); HR-MS calcd for C_29_H_28_O_3_N_5_ (Μ + H)^+^: *m*/*z*: 494.2192, found: 494.2184.

*N-((1-benzyl-1H-1,2,3-triazol-4-yl)methyl)-4-hydroxy-1-(3-methylbut-2-en-1-yl)-2-oxo-1,2-dihydroquinoline-3-carboxamide* (**5g**)

Prepared according to the general method (D), in a quartz vessel, 0.44 mmol (28.6 mg) NaN_3_, 0.44 mmol (61.3 μL) Et_3_N, 0.4 mmol (52.3 μL) benzyl bromide, 0.09 mmol (17.8 mg) sodium ascorbate, 0.11 mmol (14.4 mg) CuSO_4_ and 0.44 mmol (136.6 mg) of carboxamide (**4d**) were dissolved in about 7mL of 1:1 t-BuOH/H_2_O solution. After the work-up procedure, the product (**5g**) was subjected to further purification via column chromatography, using PE/EtOAc 60:40 as eluent and was obtained as white solid. Yield: 52% (101.5 mg); M.p. 109–114 °C; ^1^HNMR (600 MHz, CDCl_3_): *δ* (ppm) 16.72 (s, 1H, C-OH), 10.74 (brs, 1H, -NH), 8.20 (d, *J* = 7.8 Hz, 1H, Ar-H), 7.65 (t, *J* = 7.8 Hz, 1H, Ar-H), 7.50 (s, 1H, N-**CH**=C-), 7.38–7.34 (m, 3H, Ar-H), 7.29–7.27 (m, 4H, Ar-H), 5.50 (s, 2H, N-**CH_2_**-Ar), 5.08 (t, *J* = 5.4 Hz, 1H, N-CH_2_-**CH**=), 4.86 (d, *J* = 4.8 Hz, 2H, N-**CH_2_**-CH=), 4.72 (br, 2H, NH-**CH_2_-**), 1.87 (s, 3H, -CH_3_), 1.72 (s, 3H, -CH_3_); ^13^C NMR (75 MHz, CDCl_3_): *δ* (ppm) 171.8, 171.3, 162.4, 145.2, 139.5, 136.3, 134.7, 133.8, 129.2, 128.8, 128.2, 125.6, 122.3, 119.3, 116.4, 114.8, 96.9, 54.3, 40.8, 34.9, 25.7, 18.5; IR > 3000 cm^−1^ ($\tilde{\nu }$, aromatic C-H), 2973.7 cm^−1^ ($\tilde{\nu }$, aliphatic C-H), 1646.91 cm^−1^ ($\tilde{\nu }$, 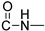


), 1563.99 cm^−1^ ($\tilde{\nu }$, N-H), 1319.07 cm^−1^ ($\tilde{\nu }$, C-N), 757.888 cm^−1^ ($\tilde{\delta }$, aromatic monosubstitution); HR-MS calcd for C_25_H_26_O_3_N_5_ (Μ + H)^+^: *m*/*z*: 444.2036, found: 444.2026.

*1-cinnamyl-4-hydroxy-N-((1-(3-methylbut-2-en-1-yl)-1H-1,2,3-triazol-4-yl)methyl)-2-oxo-1,2-dihydroquinoline-3-carboxamide* (**5h**)

Prepared according to the general method (D), in a quartz vessel, 0.25 mmol (24.7 mg) NaN_3_, 0.25 mmol (34.9 μL) Et_3_N, 0.25 mmol (44.2 μL) prenyl bromide, 0.05 mmol (9.9 mg) sodium ascorbate, 0.05 mmol (8.0 mg) CuSO_4_ and 0.25 mmol (90.0 mg) of carboxamide (**4g**) were dissolved in about 7mL of 1:1 t-BuOH/H_2_O solution. After the work-up procedure, the product (**5h**) was subjected to further purification via column chromatography, using PE/EtOAc 60:40 as eluent and was obtained as white solid. Yield: 33% (38.7 mg); M.p. 92.0–95.5 °C; ^1^H NMR (600 MHz, CDCl_3_): *δ* (ppm) 16.89 (s, 1H, -OH), 10.70 (brs, 1H, -NH), 8.24 (d, *J* = 7.8 Hz, 1H, Ar-H), 7.66 (t, *J* = 8.4 Hz, 1H, Ar-H), 7.55 (s, 1H, N-**CH**=C-), 7.40 (d, *J* = 9.0 Hz, 1H, Ar-H), 7.32–7.31 (m, 3H, Ar-H), 7.29–7.27 (m, 2H, Ar-H), 7.22–7.20 (m, 1H, Ar-H), 6.48 (d, *J* = 15.6 Hz, 1H, N-CH_2_-CH=**CH**-Ar), 6.28 (dt, *J* = 16.2, 5.4 Hz, 1H, N-CH_2_-**CH**=CH-Ar), 5.42 (t, *J* = 7.2 Hz, 1H, N-CH_2_-**CH**=C-), 5.06 (d, *J* = 4.2 Hz, 2H, N-**CH_2_**-CH=CH-Ar), 4.94 (br, 2H, NH-**CH_2_**), 4.76 (d, *J* = 6.0 Hz, 2H, N-**CH_2_**-CH=C-), 1.79 (br, 6H, 2x -CH_3_); ^13^C NMR (150 MHz, CDCl_3_): *δ* (ppm) 172.2, 171.3, 162.5, 144.6, 139.9, 139.6, 136.2, 134.0, 132.6, 128.7, 128.0, 126.5, 125.8, 123.2, 122.6, 121.7, 117.4, 116.5, 115.0, 96.9, 48.2, 44.1, 35.0, 25.9, 18.2; HR-MS calcd for C_27_H_28_O_3_N_5_ (Μ − H)^−^: *m*/*z*: 468.2036, found: 468.2033.

*1-ethyl-4-hydroxy-N-((1-(3-methylbut-2-en-1-yl)-1H-1,2,3-triazol-4-yl)methyl)-2-oxo-1,2-dihydroquinoline-3-carboxamide* (**5i**)

Prepared according to the general method (D), in a quartz vessel, 0.80 mmol (52.0 mg) NaN_3_, 0.80 mmol (111.7 μL) Et_3_N, 0.80 mmol (87.5 μL) prenyl bromide, 0.08 mmol (15.8 mg) sodium ascorbate, 0.08 mmol (12.8 mg) CuSO_4_ and 0.80 mmol (200.0 mg) of carboxamide (**4c**) were dissolved in about 7mL of 1:1 t-BuOH/H_2_O solution. After the work-up procedure, the product (**5i**) was subjected to further purification via column chromatography, using PE/EtOAc 60:40 as eluent and was obtained as white solid. Yield: 30% (91.5 mg); M.p. 100–104 °C; ^1^H NMR (600 MHz, CDCl_3_): *δ* (ppm) 16.74 (s, 1H, -OH), 10.76 (brs, 1H, -NH), 8.24 (dd, *J* = 7.8, 1.2 Hz, 1H, Ar-H), 7.69 (ddd, *J* = 8.1, 7.4, 1.8 Hz, 1H, Ar-H), 7.55 (s, 1H, H-5′), 7.37 (d, *J* = 9.0 Hz, 1H, Ar-H), 7.30 (t, *J* = 7.8 Hz, 1H, Ar-H), 5.43 (t, *J* = 7.2 Hz, 1H, N-CH_2_-**CH**=), 4.93 (br, 2H, NH-**CH_2_**-), 4.74 (d, *J* = 6.0 Hz, 2H, N-**CH_2_**-CH=), 4.31 (q, *J* = 7.2 Hz, 2H, N-**CH_2_**-CH_3_), 1.79 (br, 6H, H-15, CH=C-(**CH_3_**)_2_), 1.33 (t, *J* = 7.2 Hz, 3H, N-CH_2_-**CH_3_**); ^13^C NMR (75 MHz, CDCl_3_): *δ* (ppm) 171.8, 171.3, 162.3, 144.7, 139.8, 139.2, 133.9, 125.8, 122.3, 121.7, 117.4, 116.5, 114.2, 97.0, 48.2, 37.3, 35.0, 25.8, 18.2, 13.0; HR-MS calcd for C_20_H_23_N_5_O_3_ (Μ + H)^+^: *m*/*z*: 382.1879, found: 382.1872.

*1-benzyl-4-hydroxy-N-((1-(3-methylbut-2-en-1-yl)-1H-1,2,3-triazol-4-yl)methyl)-2-oxo-1,2-dihydroquinoline-3-carboxamide* (**5j**)

Prepared according to the general method (D), in a quartz vessel, 0.60 mmol (39.0 mg) NaN_3_, 0.60 mmol (83.8 μL) Et_3_N, 0.60 mmol (65.6 μL) prenyl bromide, 0.06 mmol (11.9 mg) sodium ascorbate, 0.06 mmol (9.6 mg) CuSO_4_ and 0.60 mmol (200.0 mg) of carboxamide (**4b**) were dissolved in about 7mL of 1:1 t-BuOH/H_2_O solution. After the work-up procedure, the product (**5j**) was subjected to further purification via column chromatography, using PE/EtOAc 60:40 as eluent and was obtained as white solid. Yield: 22% (58.5 mg); M.p. 120.0–122.0 °C; ^1^H NMR (600 MHz, CDCl_3_): *δ* (ppm) 16.93 (s, 1H, -OH), 10.69 (brs, 1H, -NH), 8.21 (dd, *J* = 8.4, 1.2 Hz, 1H, Ar-H), 7.56 (s, 1H, H-5′), 7.54 (td, *J* = 8.4, 1.21 Hz, 1H, Ar-H), 7.30–7.25 (m, 3H, Ar-H), 7.24–7.22 (m, 2H, Ar-H), 7.16 (d, *J* = 7.2 Hz, 2H, Ar-H), 5.48 (br, 2H, N-**CH_2_**-Ar), 5.42 (t, *J* = 6.6 Hz, 1H, N-CH_2_-**CH**=), 4.93 (d, *J* = 7.8 Hz, 2H, N-**CH_2_**-CH=), 4.75 (d, *J* = 6 Hz, 2H, NH-**CH_2_**-), 1.79 (s, 3H, -CH_3_), 1.78 (s, 3H, -CH_3_); ^13^C NMR (150 MHz, CDCl_3_): *δ* (ppm) 172.3, 171.2, 162.8, 144.5, 139.8, 139.6, 136.2, 134.0, 129.0, 127.5, 1263, 125.7, 122.6, 121.7, 117.3, 116.5, 115.2, 96.8, 48.1, 45.8, 35.0, 25.8, 18.2; HR-MS calcd for C_20_H_23_N_5_O_3_ (Μ − H)^−^: *m*/*z*: 442.1879, found: 442.1878.

### 2.2. Biological In Vitro Assays

The in vitro assays were performed at a concentration of 100 µM (a 10 mM stock solution in DMSO was used, from which several dilutions were made for the determination of IC_50_ values), at least in triplicate, and the standard deviation of absorbance was less than 10% of the mean. The compounds were diluted in 0.1% DMSO under sonification in an appropriate buffer in several dilutions. Statistical comparisons were made using Student’s t-test. A statistically significant difference was defined as *p* < 0.05. The results were compared to the corresponding reference compounds.

#### 2.2.1. Inhibition of Linoleic Acid Peroxidation Induced by AAPH Radical

The determination of the ability of the synthesized compounds to inhibit linoleic acid peroxidation induced by the radical initiator 2,2-Azobis(2-amidinopropane) dihydrochloride (AAPH) was performed according to Hadjipavlou-Litina et al. [56]. Ten microliters of the 16 mM sodium linoleate solution were added to the UV cuvette containing 0.93 mL of a 0.05 M phosphate buffer, pH 7.4, pre-thermostated at 37 °C. The oxidation reaction was initiated at 37 °C under air by the addition of 50 µL of a 40 mM AAPH solution, which was used as a free radical initiator. Oxidation was carried out in the presence of aliquots (10 µL) in the assay without antioxidants and monitored at 234 nm. Lipid oxidation was recorded in the presence of the same level of DMSO served as a negative control. results are compared to the appropriate standard compound Trolox.

#### 2.2.2. Soybean LOX Inhibition Study In Vitro

The determination of the inhibition of soybean LOX for the synthesized compounds was performed according to Hadjipavlou-Litina et al. [56]. The tested compounds were incubated at room temperature with sodium linoleate (0.1 mM) and 0.2 mL of enzyme solution (1/9 × 10^−4^ *w*/*v* in saline). The method was based on the conversion of sodium linoleate to 13-hydroperoxylinoleic acid at 234 nm. In order to determine the IC5_0_ values, different concentrations were used. The results are compared to the appropriate standard compound Nordihydroguaiaretic acid (NDGA) (positive control).

#### 2.2.3. Cell Viability and Cytotoxicity Assay

The determination of the cell viability of HaCaT cells and the cell toxicity of A549 and A375 cell lines was performed according to Myriagkou et al. [57], via the MTT (3-(4,5-dimethylthiazol-2-yl)-2,5-diphenyltetrazolium bromide) assay. In a 96-well plate, cells were seeded at specific concentrations (30 × 10^4^ cells/mL for HaCaT cells and 20 × 10^4^ cells/mL for A549 and A375 cells) and incubated (37 °C, 5% CO_2_) for 24 h. Following this first incubation period, solutions of the tested compounds at various concentrations were added to the cells, and the plates were incubated for an additional 24 h. Upon the second incubation period, the appropriate volume of MTT solution (1 mg/mL in PBS, filtrated through a 0.22-μm filter) was added to each well, and the plate was incubated for 4 h, during which formazan crystals were formed. The crystals were dissolved by addition of 100 μL od DMSO in each cell and after 30 min of stirring, the absorbance was recorded at 450 nm using a microplate reader. All tests were undertaken on three replicates and compared to the control (untreated cells), the blank (medium), and the reference control (cells treated with 100 μM silibinin).

## 3. Results and Discussion

### 3.1. Chemistry

**Scheme 1:**
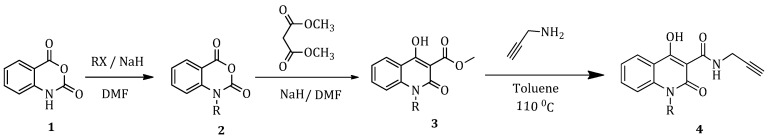
Synthetic procedure of carboxamides **4a–4g**.

Isatoic anhydride (**1**) underwent a N-alkylation reaction with the corresponding alkyl halide in dimethylformamide (DMF), utilizing NaH as the base. The resulting N-substituted isatoic anhydride (**2**) was used as an acylating agent of dimethyl malonate giving the corresponding quinolinone (**3**). In the next step of the synthetic process, the synthesis of the amino carboxamide (**4**) took place via a nucleophilic acyl-substitution reaction between quinolinone and propargylamine (Scheme 1).

**Scheme 2:**
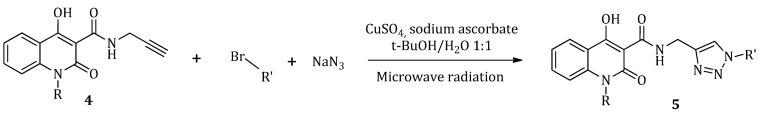
Synthetic procedure of **5a–5j**.

The synthesis of the quinolinone–triazole hybrid molecules (**5**) was realized via a copper catalyzed 1,3-cycloaddition reaction between the carboxamide (**4**), sodium azide, and the appropriate bromide, using CuSO_4_ and sodium ascorbate as catalyst and triethylamine as a base in H_2_O:t-BuOH in a 1:1 ratio (Scheme 2). The reaction was performed under microwave irradiation, allowing small reaction time (10 min) and satisfactory yields (18–53%).

The synthesized compounds, not previously described in the literature (**4a–4g**, **5a–5j**), were structurally characterized via ^1^H and ^13^C NMR, FT-IR, and HRMS spectroscopy (see the Supplementary Materials).

The ^1^H-NMR spectra of the quinolinone–triazole hybrid molecules were characterized by a signal at around 7.5 ppm, which was attributed to the vinylic proton of the triazole ring, confirming the synthesis of the compounds. Moreover, two more characteristic peaks appear at these spectra, including a downfield signal (16.7–16.9 ppm) corresponding to the proton of the 4-OH group, deshielded as a consequence of strong intramolecular hydrogen bonding with the adjacent carbonyl group, as well as a signal at ≈10.7 ppm, which was attributed to the proton of the amide bond (-NH).

**Scheme 3:**
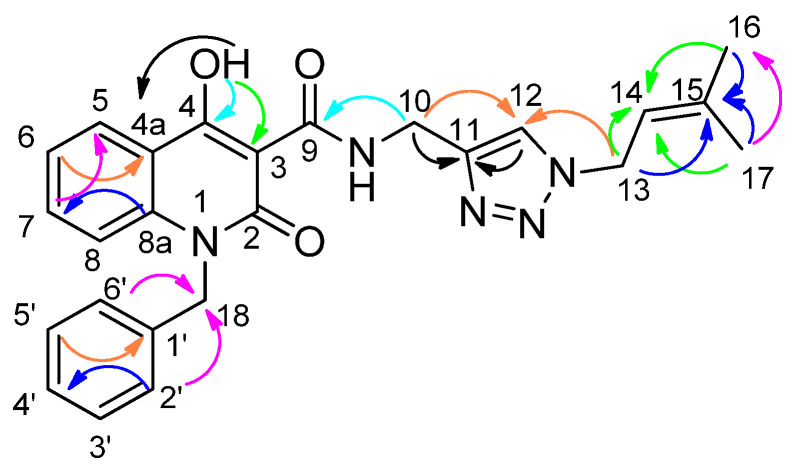
Chemical structure and main HMBC correlations of compound **5j**.

A detailed assignment of the ^13^C NMR spectrum (Table 1) was performed for the novel compound **5j**, which was selected as a representative compound, using ^13^C, 2D heteronuclear HSQC and HMBC NMR spectra (Supplementary Materials). The key interactions observed in the HMBC spectrum are illustrated in Scheme 3, while the connectivity between each proton and its corresponding carbon was determined through the HSQC experiment.

### 3.2. Bioactivity Assessment

The synthesized quinolinone derivatives (Table 2) were examined for their antioxidant activity, through the inhibition of lipid peroxidation. The inhibitory activity of the compounds against soybean lipoxygenase (LOX) was evaluated as an indicator of their anti-inflammatory potential. Finally, all quinolinone analogs **4a–4g** and the hybrids **5a–5i** were evaluated for cell viability via the MTT method, using HaCaT epithelial cells, as well as for their cytotoxicity against A549 and A375 cancer lines.

#### 3.2.1. Evaluation of Antioxidant Activity

Free radicals are reactive chemical species produced during normal biochemical processes in aerobic organisms, capable of attacking and damaging biological targets such as lipids, proteins, and DNA, thereby contributing to the development of numerous diseases. Normally, the organisms exploit enzymes or small molecules, called endogenous antioxidants, to scavenge free radicals. However, due to factors, such as stress and radiation, the production of free radicals is greater than what the organism can cope with. In such cases, exogenous antioxidants are required to counteract the damage induced by oxidative stress. Antioxidants are generally described as molecules that, even in small amounts, can significantly delay or inhibit the oxidation of readily oxidizable substrates following different chemical mechanisms: (i) neutralizing free radicals by donating an electron to the free radical, which stabilizes it; (ii) interrupting chain reactions; (iii) transferring a hydrogen atom or a single electron. It should be noted that the efficacy of compounds displaying strong antioxidant activity in vitro is still considered controversial, with several studies questioning their translation from in vitro to in vivo activity [58].

In an attempt to assess the in vitro antioxidant potential of the synthesized compounds, their inhibitory activity against lipid peroxidation of linoleic acid sodium salt was examined, induced by AAPH radical under atmospheric oxygen at 37 °C. The anti-lipid peroxidation was recorded with UV spectrophotometrically. The assay is a quick and widely used method for the assessment of antioxidant activity.

In Table 3, the results of the antioxidant activity studies of carboxamides **4a–4c** and **4e–4g** and quinolinone–triazole hybrid molecules **5a–5j** are given, in addition to their ability to inhibit the activity of the LOX enzyme. Additionally, the clogP values of the compounds are presented.

Regarding the inhibitory ability of the tested compounds against AAPH-induced lipid peroxidation, the results indicate that among the carboxamides, **4c** and **4f** analogues, possessing an ethyl and a 3-phenyl-propyl substitution, respectively, at the nitrogen of the heterocyclic ring, demonstrating the highest activity (100% and 81.1% at 100 μM concentration, respectively). As for the quinolinone–triazole hybrid molecules examined, it is evident that the majority of them present very high inhibition of lipid peroxidation, between 87.0% and 98%. It is noteworthy that the presence of the ethyl- and 3-phenyl-propyl substituent on the nitrogen of the quinolinone ring, which increased the antioxidant activity of the corresponding carboxamides, also leads to hybrid molecules that are potent inhibitors of lipid peroxidation (N-ethyl hybrids **5c**, **5d**, **5e**, **5i**: 91.0–96.0%, and N-3-phenyl-propyl hybrid **5f**: 98.0%). It is therefore concluded that the structural characteristic that plays the most important role in this assay for the hybrid molecules is the nitrogen substituent of the quinolinone and not the nitrogen substituent of the triazole ring. This is also confirmed in the case of carboxamide **4g** and its derivative **5h**, which have a cinnamoyl substituent on the quinolinone nitrogen, and both show very weak antioxidant activity (27.6% and 18.7%, respectively).

#### 3.2.2. Evaluation of Soybean LOX Inhibitory Activity

As evidence of anti-inflammatory activity, the quinolinone derivatives were examined for their ability to inhibit soybean lipoxygenase (LOX). LOX catalyzes the cleavage of arachidonic acid to leukotrienes that are found to play a crucial role in the pathogenesis of some inflammatory diseases. For this study, soybean LOX was employed, as it is a plant enzyme exhibiting sufficient homology to human 5-LOX.

Regarding the ability of the molecules to inhibit the activity of the LOX enzyme, it is observed that among the carboxamides that were evaluated, **4c** analogue, which possess an N-ethyl substituent, is the most powerful inhibitor, with an IC_50_ value of 22.5 μM. The presence of a N-benzyl substituent (carboxamide **4b**) or an increase in the aliphatic chain from two carbons to eight (carboxamide **4e**) leads to weaker inhibitors (IC_50_ = 43.2 μM and 48.3 μM, respectively). Furthermore, upon comparison of amides **4f** and **4g**, the results indicate that the presence of a double bond at the substituent on the heterocyclic nitrogen, converts an inactive molecule (**4f**: 49.3% at 0.1 mM) to a satisfactory LOX inhibitor (**4g**: IC_50_ = 51.2 μM). In general, it appears that the less bulky the quinolinone nitrogen substituent, the stronger the molecule’s ability to inhibit enzyme activity. In the case of the examined quinolinone–triazole hybrid molecules, the strongest inhibitor is hybrid **5a** (IC_50_ = 10.0 μM), with a methyl substituent on the quinolinone nitrogen and a benzyl substituent on the triazole nitrogen. Maintaining the benzyl substituent on the triazole nitrogen and adding a benzyl-(compound **5b**), ethyl-(compound **5c**), 3-phenyl-propyl-(compound **5f**), or prenyl-(compound **5g**) substituent on the quinolinone nitrogen leads to weaker LOX inhibitors (IC_50_ = 46.0 μM, 51.5 μM, 50.0 μM and 75.0 μM, respectively). Τhis observation is in accordance with the results obtained for the corresponding carboxamides, confirming that a bulky substituent attached to the quinoline nitrogen negatively affects the anti-inflammatory effect of the molecules.

Lipophilicity is a physicochemical property which can be related to LOX inhibition and usually a more lipophilic molecule is expected to interact better with the hydrophobic active site of the enzyme. However, the results obtained in this study do not show a clear trend between LOX inhibition and increased lipophilicity. On the contrary, the less lipophilic compounds 5a (ClogP 2.163) and 4c (ClogP 2.228) show the best LOX inhibitory activity among the tested compounds, whereas compounds with ClogP 4–5.4 show significantly lower LOX inhibition.

#### 3.2.3. Evaluation of Cell Viability in HaCat Cells

The cytotoxicity of the novel compounds was also measured via the mehtylthiazol tetrazolium (MTT) assay. MTT is a water-soluble tetrazolium salt that when diluted to media that does not contain phenol red, it provides a yellow solution. However, when mitochondrial dehydrogenase enzymes of living cells affect MTT, cleavage of the tetrazolium ring occurs, converting the yellow MTT solution to a water-insoluble purple formazan. This purple formazan can be diluted to dimethylsulfoxide (DMSO), and its absorbance can be measured spectrophotometrically. The MTT assay determines the number of living cells via bioproduction of MTT into a purple formazan product that can be detected by absorbance at 570 nm.

Table 4 presents the results of the bioactivity study of carboxamides **4a–4c** and **4e–4g** and quinolinone–triazole hybrid molecules **5a–5h** and **5j** in terms of cell viability in Human Epidermal Ceratinocyte (HaCaT) cells and in terms of their cytotoxicity in two cancer cell lines, adenocarcinomic human alveolar basal epithelial cells (A549 cell line) and human melanoma cells (A375 cell line).

Regarding cell viability, the percentage of HaCaT epithelial cells that survived after exposure to the tested molecules was determined. The evaluation of the results of the experiment is a first indication of the cytotoxicity of the compounds.

The carboxamides synthesized and studied in this work showed encouraging results, with cell viability rates between 61.5% and 100%. More specifically, compound **4g**, which possesses a cinnamyl substituent attached to the nitrogen of the quinolinone heterocyclic ring, is shown to be the safest compound, with 100% cell survival; this is followed by carboxamide **4c**, bearing an N-ethyl substituent, with 90.5% cell survival. Compound **4a,** bearing a N-methyl substituent, yielded the lowest survival rate (61.5%) among all the tested compounds.

The majority of the quinolinone–triazole hybrid molecules evaluated in this study showed very good HaCaT cell survival results (>86.5%), with hybrids **5a**, **5c**, **5f**, **5g**, and **5j** emerging as the safest, conferring 100% viability. The only molecule among those examined in this series that showed very low cell viability (27.6%) is triazole **5e**, which possesses an ethyl substituent at the nitrogen of the quinolinone heterocyclic ring and a cinnamyl substituent at the nitrogen of the triazole ring. The presence of a cinnamyl substituent on the nitrogen of the quinolinone ring, which led to the safest carboxamide (**4g**), appears to maintain high cell viability rates also in the case of hybrid molecules, when it is combined with a prenyl substituent on the triazole ring (compound **5h**).

#### 3.2.4. Evaluation of Cytotoxicity Against A549 and A375 Cancer Lines

The cytotoxicity of the quinolinone derivatives was evaluated against two cancer lines: the A549 line (carcinoma epithelial adenocarcinoma cells) and the A375 line (human melanoma cells), and the results are presented on Table 4.

All the carboxamides tested showed moderate–low or no cytotoxicity against the A549 series. More specifically, the most active is the N-benzyl derivative **4b** with 45.7% toxicity at a concentration of 100 μM, followed by the N-cinnamyl derivative **4g** and the N-octyl derivative **4e**, with 35.2% and 33.7% cytotoxicity, respectively. The latter showed the strongest activity against the A375 cell line (22.6% at a concentration of 100 μM), while the rest of the derivatives showed very low activity (from 0 to 11.7% at a concentration of 100μM).

In the case of the tested triazoles, the majority of them did not show significant cytotoxicity in the tested cell lines. Derivative **5b** showed cytotoxicity against the A549 line (49.9% at a concentration of 100μM), while triazoles **5e** and **5g** inhibited the growth of A375 cells by 35.2% and 46.8%, respectively, at a concentration of 100 μM.

## 4. Conclusions

The present study describes the design, synthesis, and evaluation of the bioactivity of seven new carboxamides (**4a–4g**) and ten new quinolinone–triazole hybrid molecules (**5a–5j**) through the copper-catalyzed 1,3-dipolar cycloaddition reaction between alkynes and azides.

The new quinolinone derivatives were evaluated for their antioxidant capacity, for their ability to inhibit the activity of soybean lipoxygenase (LOX), for cell viability through the method MTT, using HaCaT epithelial cells and for their cytotoxicity against A549 and A375 cancer lines. The influence of the different substituents ((i) in the nitrogen of the quinolinone part and (ii) in the triazole nitrogen) on the bioactivity of the new molecules was studied.

According to the results, it seems that the carboxamide **4c** presents the best combination of antioxidant and anti-inflammatory activity (100% anti-lipid peroxidation and IC_50_ = 22.5 μM for the inhibition of LOX), while among the quinolinone–triazole hybrids, molecule **5a** was revealed as the best agent (98.0% for the inhibition of lipid peroxidation and IC_50_ = 10.0 μM for LOX inhibition). As a general observation, the presence of less bulky substituents on the nitrogen of the quinolinone scaffold leads to stronger LOX inhibitory activity.

Regarding the assessment of cell viability in HaCaT epithelial cells, the carboxamides synthesized and studied showed encouraging results, with cell viability rates between 61.5% and 100%. In addition, the majority of the quinolinone–triazole molecules evaluated showed very good survival results of HaCaT cells, with rates higher than 86.5%. Furthermore, based on the results of the cytotoxicity against the two cancer lines A549 and A375, it emerged that the carboxamides exhibited moderate–low or even no cytotoxicity, with the N-benzyl derivative **4b** being the most active (45.7% toxicity against the A549 series). The majority of triazoles tested did not show appreciable cytotoxicity. Derivative **5b** was shown to be the most active against the A549 series (49.9%) and derivative **5g** was shown to be the most active against the A375 series (46.8%).

To conclude, the biological evaluation of the molecules carried out in this research revealed the quinolinone–triazole hybrid molecule **5a** as a potent LOX and lipid peroxidation inhibitor which can be regarded as a lead compound for the development of more active agents with dual activity.

## Figures and Tables

**Table 1:** ^1^H and ^13^C NMR data of compound **5j** in CDCl_3_.

Position	δ_H_, Multi. (*J*)	δ_c_, Multi. (*J*)	Position	δ_H_, Multi. (*J*)	δ_c_, Multi. (*J*)
1	-	-	12	7.56, s	122.0
2	-	162.82	13	4.93, d (*J* = 7.8 Hz)	48.0
3	-	96.8	14	5.42, t (*J* = 7.8 Hz)	119.8
4	-	172.3	15	-	140
4a	-	119.0	16	1.79, s	28.3
5	8.21, dd (*J* = 8.4, 1.2Hz)	125.7	17	1.78, s	20.8
6	7.24–7.22, m	115.0	18	5.48, br	45.9
7	7.54, td (*J* = 8.4, 1.2 Hz)	133.9	1′	-	136.0
8	7.30–7.25, m	122.6	2′/6′	7.16, d (*J* = 7.2 Hz)	126.4
8a	-	139.6	3′/5′	7.30–7.25, m	129.0
9	-	171.3	4′	7.24–7.22, m	127.5
10	4.75, d (*J* = 6 Hz)	35.0	-OH	16.93, s	-
11	-	144.0	-NH	10.69, s	-

**Table 2:** Structure of synthesized quinolinone derivatives and refence compounds.

Code	Structure	Code	Structure	Code	Structure
**4a**	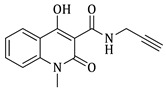	**4g**	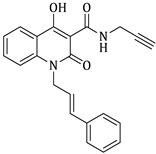	**5f**	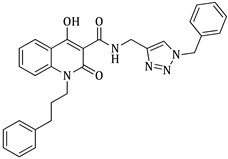
**4b**	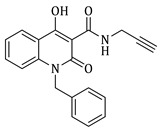	**5a**	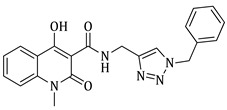	**5g**	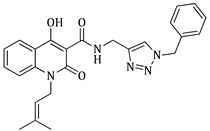
**4c**	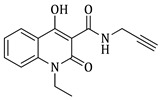	**5b**	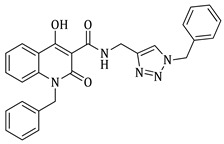	**5h**	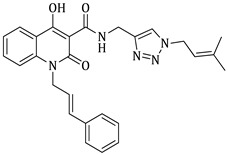
**4d**	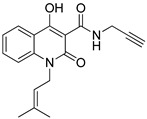	**5c**	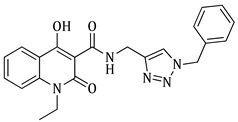	**5i**	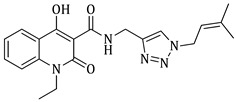
**4e**	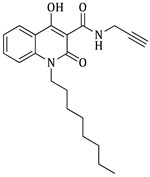	**5d**	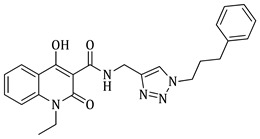	**5j**	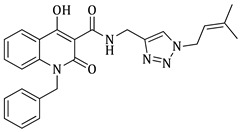
**4f**	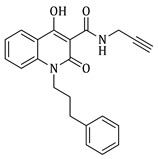	**5e**	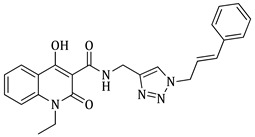		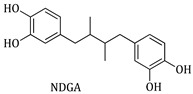
					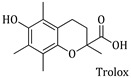

**Table 3:** Antioxidant activity and inhibitory activity against LOX and clogP of carboxamides **4a–4c** and **4e**–**4g** and hybrid molecules **5a–5j**.

Compound	Inhibition of Linoleic Acid Peroxidation (% at 100 μΜ)	Inhibition of LOX IC_50_ (μΜ) or (% at 100 μΜ)	ClogP
**4a**	42.7	nt *	1.699
**4b**	14.7	43.2 μM	3.467
**4c**	100	22.5 μM	2.228
**4e**	34.9	48.3 μM	5.402
**4f**	81.1	(49.3% at 100 μM)	4.175
**4g**	27.6	51.2 μM	4.072
**5a**	98.0	10.0 μM	2.163
**5b**	89.0	46.0 μM	3.931
**5c**	94.0	51.5 μM	2.691
**5d**	92.0	47.5 μM	3.400
**5e**	91.0	45.0 μM	3.296
**5f**	98.0	50.0 μM	4.639
**5g**	83.0	75.0 μM	3.865
**5h**	18.7	(44.5% at 100 μM)	4.469
**5i**	96.0	70.0 μM	2.626
**5j**	92.0	41.0 μM	3.865
**NDGA**	Nt	0.5 μM	-
**Trolox**	88.0	nt	-

* nt: not tested.

**Table 4:** Evaluation of carboxamides **4a–4c** and **4e–4g** and hybrid molecules **5a–5h** and **5j** for cell viability in HaCaT cells and for cytotoxicity against two cancer lines.

Compound	HaCaT Viability (% at 100 μΜ)	% Cytotoxicity A549 (100 μΜ)	% Cytotoxicity A375 (100 μΜ)
**4a**	61.5	0	7.0
**4b**	88.7	45.7	7.6
**4c**	90.5	16.4	0.2
**4e**	76.2	33.7	22.6
**4f**	76.9	0	0
**4g**	100	35.2	11.7
**5a**	100	24.2	0
**5b**	86.5	49.9	0
**5c**	100	0	0
**5d**	99.6	28.9	10.5
**5e**	27.6	7.3	35.2
**5f**	100	12.5	23.7
**5g**	100	13.4	46.8
**5h**	98.9	0	0
**5j**	100	18.8	0
**Negative control Silibinin**	47.2	57.4	12.6

## Data Availability

The original contributions presented in this study are included in the article/Supplementary Material. Further inquiries can be directed to the corresponding author.

## Supplementary Materials

The following supporting information can be downloaded at: https://www.mdpi.com/article/10.3390/biom16010029/s1, Figures S1–S64: Nuclear Magnetic Resonance data; Figures S65–S87: HR-MS data; Figures S88–S95: FT-IR data.

## Abbreviations

The following abbreviations are used in this manuscript:

AAPH	2,2′-Azobis(2-amidinopropane) dihydrochloride
CuAAC	Cu-catalyzed Azide Alkyne Cycloaddition
Cu_2_SO_4_	Copper Sulfate
DMF	Dimethylformamide
DMSO	dimethylsulfoxide
Et_2_O	Diethyl ether
Et_3_N	Triethylamine
HaCaT	Human Epidermal Ceratinocyte
LOX	Lipoxygenase
MTT	Mehtylthiazol tetrazolium
Na_2_SO_4_	Sodium sulfate
NaH	Sodium hydride
NaN_3_	Sodium azide
ROS	Reactive Oxygen Species
TLC	Thin-Layer Chromatography

## References

[1] Kolb H.C., Finn M.G., Sharpless K.B. (2001). Click Chemistry: Diverse Chemical Function from a Few Good Reactions. Angew. Chemie-Int. Ed..

[2] Kaur J., Saxena M., Rishi N. (2021). An Overview of Recent Advances in Biomedical Applications of Click Chemistry. Bioconjugate Chem..

[3] Jiang X., Hao X., Jing L., Wu G., Kang D., Liu X., Zhan P. (2019). Recent Applications of Click Chemistry in Drug Discovery. Expert Opin. Drug Discov..

[4] Chaturvedi P., Chaturvedi N., Gupta S., Mishra A., Singh M., Siddhartha T. (2011). Click Chemistry: A New Approach for Drug Discovery. Int. J. Pharm. Sci. Rev. Res..

[5] Thirumurugan P., Matosiuk D., Jozwiak K. (2013). Click Chemistry for Drug Development and Diverse Chemical-Biology Applications. Chem. Rev..

[6] Mandoli A. (2016). Recent Advances in Recoverable Systems for the Copper-Catalyzed Azide-Alkyne Cycloaddition Reaction (CuAAC). Molecules.

[7] Wei F., Wang W., Ma Y., Tung C.H., Xu Z. (2016). Regioselective Synthesis of Multisubstituted 1,2,3-Triazoles: Moving beyond the Copper-Catalyzed Azide-Alkyne Cycloaddition. Chem. Commun..

[8] Bock V.D., Hiemstra H., van Maarseveen J.H. (2006). Cu^I^-Catalyzed Alkyne–Azide “Click” Cycloadditions from a Mechanistic and Synthetic Perspective. Eur. J. Org. Chem..

[9] Rodionov V.O., Fokin V.V., Finn M.G. (2005). Mechanism of the Ligand-Free CuI-Catalyzed Azide-Alkyne Cycloaddition Reaction. Angew. Chemie-Int. Ed..

[10] Mykhalichko B.M., Temkin O.N., Mys’kiv M.G. (2000). Polynuclear Complexes of Copper(I) Halides: Coordination Chemistry and Catalytic Transformations of Alkynes. Usp. Khim..

[11] Bräse S., Gil C., Knepper K., Zimmermann V. (2005). Organic Azides: An Exploding Diversity of a Unique Class of Compounds. Angew. Chemie-Int. Ed..

[12] Dixit D., Verma P.K., Marwaha R.K. (2021). A Review on ‘Triazoles’: Their Chemistry, Synthesis and Pharmacological Potentials. J. Iran. Chem. Soc..

[13] Bachl J., Mayr J., Sayago F.J., Cativiela C., Díaz Díaz D. (2015). Amide-Triazole Isosteric Substitution for Tuning Self-Assembly and Incorporating New Functions into Soft Supramolecular Materials. Chem. Commun..

[14] Malik M.S., Ahmed S.A., Althagafi I.I., Ansari M.A., Kamal A. (2020). Application of Triazoles as Bioisosteres and Linkers in the Development of Microtubule Targeting Agents. RSC Med. Chem..

[15] Chen X.Q., Li Z.H., Liu L.L., Wang H., Yang S.H., Zhang J.S., Zhang Y. (2021). Green Extraction Using Deep Eutectic Solvents and Antioxidant Activities of Flavonoids from Two Fruits of Rubia Species. Lwt.

[16] Sudeep P., Vagish C.B., Kumar A.D., Kumar K.A. (2020). 1,2,3-Triazoles: A Review on Current Trends in Synthetic and Biological Applications. IOSR J. Appl. Chem..

[17] Rani A., Singh G., Singh A., Maqbool U., Kaur G., Jandeep S. (2020). CuAAC-Ensembled 1,2,3-Triazole-Linked Isosteres as Pharmacophores in Drug Discovery: Review. RCS Adv..

[18] Ankali K.N., Rangaswamy J., Shalavadi M., Naik N. (2021). Synthesis and Molecular Docking of Novel 1, 3-Thiazole Derived 1,2,3-Triazoles and In Vivo Biological Evaluation for Their Anti Anxiety and Anti Inflammatory Activity. J. Mol. Struct..

[19] Mady M.F., Awad G.E.A., Jørgensen K.B. (2014). European Journal of Medicinal Chemistry Sulfone Moieties by the CuAAC Reaction, and Biological Evaluation of Them as Antioxidant and Antimicrobial Agents. Eur. J. Med. Chem..

[20] Hadjipavlou-litina D., Głowacka I.E., Marco-Contelles J., Piotrowska D.G. (2023). Synthesis and Antioxidant Properties of Novel 1,2,3-Triazole-Containing Nitrones. Antioxidants.

[21] Das A., Kumar A., Singh D. (2021). Design, Synthesis, Anticancer and Antioxidant Activities of Amide Linked 1,4-Disubstituted 1,2,3-Triazoles. J. Mol. Struct..

[22] Detsi A., Bouloumbasi D., Prousis K.C., Koufaki M., Athanasellis G., Melagraki G., Afantitis A., Igglessi-Markopoulou O., Kontogiorgis C., Hadjipavlou-Litina D.J. (2007). Design and Synthesis of Novel Quinolinone-3-Aminoamides and Their α-Lipoic Acid Adducts as Antioxidant and Anti-Inflammatory Agents. J. Med. Chem..

[23] Reddy M.S., Thirukovela N.S., Narsimha S., Ravinder M., Nukala S.K. (2022). Synthesis of Fused 1,2,3-Triazoles of Clioquinol via Sequential CuAAC and C–H Arylation; in Vitro Anticancer Activity, in Silico DNA Topoisomerase II Inhibitory Activity and ADMET. J. Mol. Struct..

[24] Afonso P.P., Ferreira V.F., de Souza M.C.B.V., Jorda A.K., Almeida M.C.B., Beltrame C.O., Paiva D.P., Wardell S.M.S.V., Wardell J.L., Tiekink E.R.T. (2009). Antiviral Evaluation of N-Amino-1,2,3-Triazoles against Cantagalo Virus Replication in Cell Culture. Eur. J. Med. Chem..

[25] Najafi Z., Mahdavi M., Saeedi M., Karimpour-razkenari E. (2019). Bioorganic Chemistry Novel Tacrine-Coumarin Hybrids Linked to 1,2,3-Triazole as Anti-Alzheimer’ s Compounds: In Vitro and in Vivo Biological Evaluation and Docking Study. Bioorg. Chem..

[26] Chu X., Wang C., Liu W., Liang L., Gong K., Zhao C., Sun K. (2019). Quinoline and Quinolone Dimers and Their Biological Activities: An Overview. Eur. J. Med. Chem..

[27] Singh S., Kaur G., Mangla V., Gupta M.K. (2015). Quinoline and Quinolones: Promising Scaffolds for Future Antimycobacterial Agents. J. Enzym. Inhib. Med. Chem..

[28] Pontiki E., Hadjipavlou-Litina D. (2019). Multi-Target Cinnamic Acids for Oxidative Stress and Inflammation: Design, Synthesis, Biological Evaluation and Modeling Studies. Molecules.

[29] Pontiki E., Hadjipavlou-Litina D., Litinas K., Geromichalos G. (2014). Novel Cinnamic Acid Derivatives as Antioxidant and Anticancer Agents: Design, Synthesis and Modeling Studies. Molecules.

[30] Alkhzem A.H., Woodman T.J., Blagbrough I.S. (2022). Design and Synthesis of Hybrid Compounds as Novel Drugs and Medicines. RSC Adv..

[31] Xu J.H., Fan Y.L., Zhou J. (2018). Quinolone–Triazole Hybrids and Their Biological Activities. J. Heterocycl. Chem..

[32] Joshi M.C., Wicht K.J., Taylor D., Hunter R., Smith P.J., Egan T.J. (2013). In Vitro Antimalarial Activity, β-Haematin Inhibition and Structure-Activity Relationships in a Series of Quinoline Triazoles. Eur. J. Med. Chem..

[33] Savanur H.M., Naik K.N., Ganapathi S.M., Kim K.M., Kalkhambkar R.G. (2018). Click Chemistry Inspired Design, Synthesis and Molecular Docking Studies of Coumarin, Quinolinone Linked 1,2,3-Triazoles as Promising Anti-Microbial Agents. ChemistrySelect.

[34] D’Souza V.T., Nayak J., D’Mello D.E., Dayananda P. (2021). Synthesis and Characterization of Biologically Important Quinoline Incorporated Triazole Derivatives. J. Mol. Struct..

[35] Awolade P., Cele N., Kerru N., Singh P. (2021). Synthesis, Antimicrobial Evaluation, and in Silico Studies of Quinoline—1H-1,2,3-Triazole Molecular Hybrids. Mol. Divers..

[36] Marciniec K., Latocha M., Kurczab R., Boryczka S. (2017). Synthesis and Anticancer Activity Evaluation of a Quinoline-Based 1,2,3-Triazoles. Med. Chem. Res..

[37] Upadhyay A., Kushwaha P., Gupta S., Dodda R.P., Ramalingam K., Kant R., Goyal N., Sashidhara K.V. (2018). Synthesis and Evaluation of Novel Triazolyl Quinoline Derivatives as Potential Antileishmanial Agents. Eur. J. Med. Chem..

[38] Nesaragi A.R., Kamble R.R., Bayannavar P.K., Shaikh S.K.J., Hoolageri S.R., Kodasi B., Joshi S.D., Kumbar V.M. (2021). Microwave Assisted Regioselective Synthesis of Quinoline Appended Triazoles as Potent Anti-Tubercular and Antifungal Agents via Copper (I) Catalyzed Cycloaddition. Bioorganic Med. Chem. Lett..

[39] Ramprasad J., Kumar Sthalam V., Linga Murthy Thampunuri R., Bhukya S., Ummanni R., Balasubramanian S., Pabbaraja S. (2019). Synthesis and Evaluation of a Novel Quinoline-Triazole Analogs for Antitubercular Properties via Molecular Hybridization Approach. Bioorganic Med. Chem. Lett..

[40] Seliem I.A., Panda S.S., Girgis A.S., Moatasim Y., Kandeil A., Mostafa A., Ali M.A., Nossier E.S., Rasslan F., Srour A.M. (2021). New Quinoline-Triazole Conjugates: Synthesis, and Antiviral Properties against SARS-CoV-2. Bioorg. Chem..

[41] Hebbar N.U., Patil A.R., Gudimani P., Shastri S.L., Shastri L.A., Joshi S.D., Vootla S.K., Khanapure S., Shettar A.K., Sungar V.A. (2022). Click Approach for Synthesis of 3,4-Dihydro-2(1H) Quinolinone, Coumarin Moored 1,2,3-Triazoles as Inhibitor of Mycobacteria Tuberculosis H37RV, Their Antioxidant, Cytotoxicity and in-Silico Studies. J. Mol. Struct..

[42] Haeggström J.Z., Funk C.D. (2011). Lipoxygenase and Leukotriene Pathways: Biochemistry, Biology, and Roles in Disease. Chem. Rev..

[43] Kuhn H., Banthiya S., Van Leyen K. (2015). Mammalian Lipoxygenases and Their Biological Relevance. Biochim. Biophys. Acta-Mol. Cell Biol. Lipids.

[44] Bartsch H., Nair J. (2006). Chronic Inflammation and Oxidative Stress in the Genesis and Perpetuation of Cancer: Role of Lipid Peroxidation, DNA Damage, and Repair. Langenbeck’s Arch. Surg..

[45] Azad M.B., Chen Y., Gibson S.B. (2009). Regulation of Autophagy by Reactive Oxygen Species (ROS): Implications for Cancer Progression and Treatment. Antioxid. Redox Signal..

[46] Khansari N., Shakiba Y., Mahmoudi M. (2009). Chronic Inflammation and Oxidative Stress as a Major Cause of Age- Related Diseases and Cancer. Recent Pat. Inflamm. Allergy Drug Discov..

[47] Mathew B.B., Tiwari A., Jatawa S.K. (2011). Free Radicals and Antioxidants: A Review. J. Pharm. Res..

[48] Murata M. (2018). Inflammation and Cancer. Environ. Health Prev. Med..

[49] Korniluk A., Koper O., Kemona H., Dymicka-Piekarska V. (2017). From Inflammation to Cancer. Ir. J. Med. Sci..

[50] Tzani A., Vaitsis C., Kritsi E., Smiljkovic M., Sokovic M., Zoumpoulakis P., Detsi A. (2020). Green Synthesis of Bis-(β-Dicarbonyl)-Methane Derivatives and Biological Evaluation as Putative Anticandidial Agents. J. Mol. Struct..

[51] Kostopoulou I., Diassakou A., Kavetsou E., Kritsi E., Zoumpoulakis P., Pontiki E., Hadjipavlou-Litina D., Detsi A. (2021). Novel Quinolinone–Pyrazoline Hybrids: Synthesis and Evaluation of Antioxidant and Lipoxygenase Inhibitory Activity. Mol. Divers..

[52] Kostopoulou I., Tzani A., Chronaki K., Prousis K.C., Pontiki E., Hadjiplavlou-Litina D., Detsi A. (2023). Novel Multi-Target Agents Based on the Privileged Structure of 4-Hydroxy-2-Quinolinone. Molecules.

[53] Yang G., Cheng C., Xu G.B., Tang L., Chua K.L., Yang Y.Y. (2020). Synthesis and Antibiofilm Evaluation of 3-Hydroxy-2,3-Dihydroquinazolin-4(1H)-One Derivatives against Opportunistic Pathogen Acinetobacter Baumannii. Bioorganic Med. Chem..

[54] Hagedorn C.H., Van Beers E.H., De Staercke C. (2000). Hepatitis C Virus RNA-Dependent RNA Polymerase (NS5B Polymerase). Curr. Top. Microbiol. Immunol..

[55] Beutner G.L., Kuethe J.T., June R.V. (2007). A Practical Method for Preparation of 4-Hydroxyquinolinone Esters. J. Org. Chem..

[56] Hadjipavlou-Litina D., Garnelis T., Athanassopoulos C.M., Papaioannou D. (2009). Kukoamine A Analogs with Lipoxygenase Inhibitory Activity. J. Enzym. Inhib. Med. Chem..

[57] Myriagkou M., Papakonstantinou E., Deligiannidou G.E., Patsilinakos A., Kontogiorgis C., Pontiki E. (2023). Novel Pyrimidine Derivatives as Antioxidant and Anticancer Agents: Design, Synthesis and Molecular Modeling Studies. Molecules.

[58] Tyuryaeva I., Lyublinskaya O. (2023). Expected and Unexpected Effects of Pharmacological Antioxidants. Int. J. Mol. Sci..

