# Supplementary material for: Synthesis and Bioactivity Assessment of Novel Quinolinone–Triazole Hybrids
Source: Biomolecules. 2025 Dec 24;16(1):29. doi: 10.3390/biom16010029 (PMC12839112; doi:10.3390/biom16010029)
Supplement: Supplementary file 1 [file biomolecules-16-00029-s001.zip › biomolecules-3964908-supplementary.pdf]

Supplementary Information

Synthesis and Bioactivity Assessment of Novel Quinolinone-Triazole Hybrids

Ioanna Kostopoulou[1], Maria-Anna Karadendrou[1], Manolis Matzapetakis[2], Maria Zervou[2], Georgia-Eirini Deligiannidou[3], Christos Kontogiorgis[3], Eleni Pontiki[4], Dimitra Hadjiplavlou-Litina[4], Anastasia Detsi[1,*]

1 Laboratory of Organic Chemistry, Department of Chemical Sciences, School of Chemical Engineering, National Technical University of Athens, Heroon Polytechniou 9, Zografou Campus, 15780 Athens, Greece; ioanna.th.kostopoulou@gmail.com (I.K.); mariannakaradendrou@mail.ntua.gr (M.-A.K.)

2 Institute of Chemical Biology, National Hellenic Research Foundation, 48 Vas. Constantinou Ave., 11635 Athens, Greece; matzman@eie.gr (M.M.); mzervou@eie.gr (M.Z.)

3 Laboratory of Hygiene and Environmental Protection, Department of Medicine, Democritus University of Thrace, 68100 Alexandroupolis, Greece; edeligia@med.duth.gr (G.-E.D.); ckontogi@med.duth.gr (C.K.)

4 Laboratory of Pharmaceutical Chemistry, School of Pharmacy, Faculty of Health Sciences, Aristotle University of Thessaloniki, 54124 Thessaloniki, Greece; epontiki@pharm.auth.gr (E.P.); hadjipav@pharm.auth.gr (D.H.-L.)

* Correspondence: adetsi@chemeng.ntua.gr; Tel.: +30-210-772-4126

Table of contents

Figure S1. ^{1}H NMR spectrum of **2a** 5
Figure S2. ^{13}C NMR spectrum of **2a** 6
Figure S3. ^{1}H NMR spectrum of **2b** 6
Figure S4. ^{13}C NMR spectrum of **2b** 7
Figure S5. ^{1}H NMR spectrum of **2c** 7
Figure S6. ^{13}C NMR spectrum of **2c** 8
Figure S7. ^{1}H NMR spectrum of **2d** 8
Figure S8. ^{13}C NMR spectrum of **2d** 9
Figure S9. ^{1}H NMR spectrum of **2e** 9
Figure S10. ^{13}C NMR spectrum of **2e** 10
Figure S11. ^{1}H NMR spectrum of **2f** 10
Figure S12. ^{13}C NMR spectrum of **2f** 11
Figure S13. ^{1}H NMR spectrum of **2g** 11
Figure S14. ^{13}C NMR spectrum of **2g** 12
Figure S15. ^{1}H NMR spectrum of **3a** 12
Figure S16. ^{13}C NMR spectrum of **3a** 13
Figure S17. ^{1}H NMR spectrum of **3b** 13
Figure S18. ^{13}C NMR spectrum of **3b** 14
Figure S19. ^{1}H NMR spectrum of **3c** 14
Figure S20. ^{13}C NMR spectrum of **3c** 15
Figure S21. ^{1}H NMR spectrum of **3d** 15
Figure S22. ^{13}C NMR spectrum of **3d** 16
Figure S23. ^{1}H NMR spectrum of **3e** 16
Figure S24. ^{13}C NMR spectrum of **3e** 17
Figure S25. ^{1}H NMR spectrum of **3f** 17
Figure S26. ^{13}C NMR spectrum of **3f** 18
Figure S27. ^{1}H NMR spectrum of **3g** 18
Figure S28. ^{13}C NMR spectrum of **3g** 19
Figure S29. ^{1}H NMR spectrum of **4a** 19
Figure S30. ^{13}C NMR spectrum of **4a** 20
Figure S31. ^{1}H NMR spectrum of **4b** 20
Figure S32. ^{13}C NMR of compound **4b** 21
Figure S33. ^{1}H NMR of compound **4c** 21
Figure S34. ^{13}C NMR of compound **4c** 22
Figure S35. ^{1}H NMR of compound **4d** 22
Figure S36. ^{13}C NMR of compound **4d** 23
Figure S37. ^{1}H NMR of compound **4e** 23
Figure S38. ^{13}C NMR of compound **4e** 24
Figure S39. ^{1}H NMR of compound **4f** 24
Figure S40. ^{13}C NMR of compound **4f** 25
Figure S41. ^{1}H NMR of compound **4g** 25

Figure S42. ^{13}C NMR of compound **4g** 26
Figure S43. ^{1}H NMR of compound **5a** 26
Figure S44. ^{13}C NMR of compound **5a** 27
Figure S45. ^{1}H NMR of compound **5b** 27
Figure S46. ^{13}C NMR of compound **5b** 28
Figure S47. ^{1}H NMR of compound **5c** 28
Figure S48. ^{13}C NMR of compound **5c** 29
Figure S49. ^{1}H NMR of compound **5d** 29
Figure S50. ^{13}C NMR of compound **5d** 30
Figure S51. ^{1}H NMR of compound **5e** 30
Figure S52. ^{13}C NMR of compound **5e** 31
Figure S53. ^{1}H NMR of compound **5f** 31
Figure S54. ^{13}C NMR of compound **5f** 32
Figure S55. ^{1}H NMR of compound **5g** 32
Figure S56. ^{13}C NMR of compound **5g** 33
Figure S57. ^{1}H NMR of compound **5h** 33
Figure S58. ^{13}C NMR of compound **5h** 34
Figure S59. ^{1}H NMR of compound **5i** 34
Figure S60. ^{13}C NMR of compound **5i** 35
Figure S61. ^{1}H NMR of compound **5j** 35
Figure S62. ^{13}C NMR of compound **5j** 36
Figure S63. ^{1}H-^{13}C HMBC NMR spectrum of **5j** 36
Figure S64. ^{1}H-^{13}C HSQC NMR spectrum of **5j** 37
Figure S65. HR-MS spectrum of **3b** 37
Figure S66. HR-MS spectrum of **3c**. 38
Figure S67. HR-MS spectrum of **3d** 38
Figure S68. HR-MS spectrum of **3e** 39
Figure S69. HR-MS spectrum of **3f** 39
Figure S70. HR-MS spectrum of **3g** 40
Figure S71. HR-MS spectrum of **4a** 40
Figure S72. HR-MS spectrum of **4b**. 41
Figure S73. HR-MS spectrum of **4c** 41
Figure S74. HR-MS spectrum of **4d** 42
Figure S75. HR-MS spectrum of **4e** 42
Figure S76. HR-MS spectrum of **4f** 43
Figure S77. HR-MS spectrum of **4g** 43
Figure S78. HR-MS spectrum of **5a** 44
Figure S79. HR-MS spectrum of **5b** 44
Figure S80. HR-MS spectrum of **5c** 45
Figure S81. HR-MS spectrum of **5d** 45
Figure S82. HR-MS spectrum of **5e** 46
Figure S83. HR-MS spectrum of **5f** 46
Figure S84. HR-MS spectrum of **5g** 47

Figure S85. HR-MS spectrum of **5h** 47
Figure S86. HR-MS spectrum of **5i** 48
Figure S87. HR-MS spectrum of **5j** 48
Figure S88. FT-IR spectrum of compound **5a**... 49
Figure S89. FT- IR spectrum of compound **5b** 49
Figure S90. FT- IR spectrum of compound **5c**.. 50
Figure S91. FT- IR spectrum of compound **5d** 50
Figure S92. FT- IR spectrum of compound **5e**.. 51
Figure S93. FT- IR spectrum of compound **5f** 51
Figure S94. FT- IR spectrum of compound **5g** 52
Figure S95. FT-IR spectrum of compound **5j** 52

All newly synthesized compounds were fully characterized by NMR (^{1}H and ^{13}C) spectroscopy and HR-MS spectroscopy, while FT-IR spectra were obtained for the synthesized hybrid molecules (**5a**-**5j**). The spectra included below represent the complete analytical dataset obtained in this study and confirm the structures and purity of the compounds.

<u>Nuclear Magnetic Resonance data</u>

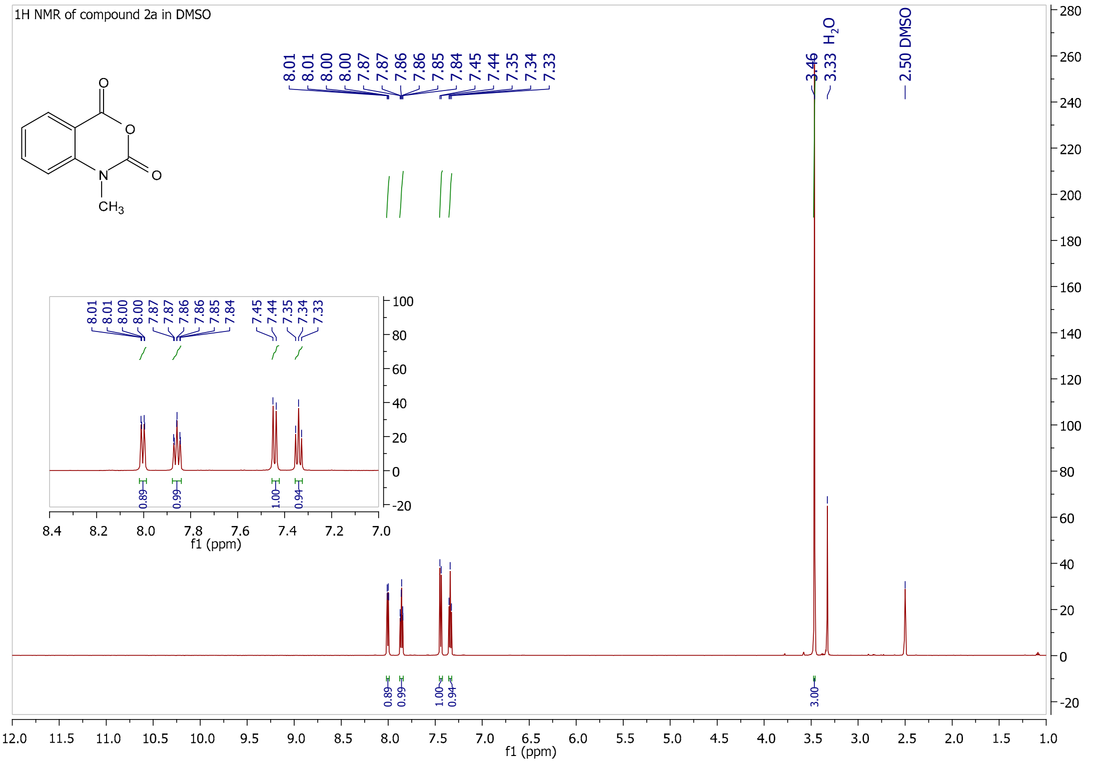

Figure S1. ^{1}H NMR spectrum of **2a**

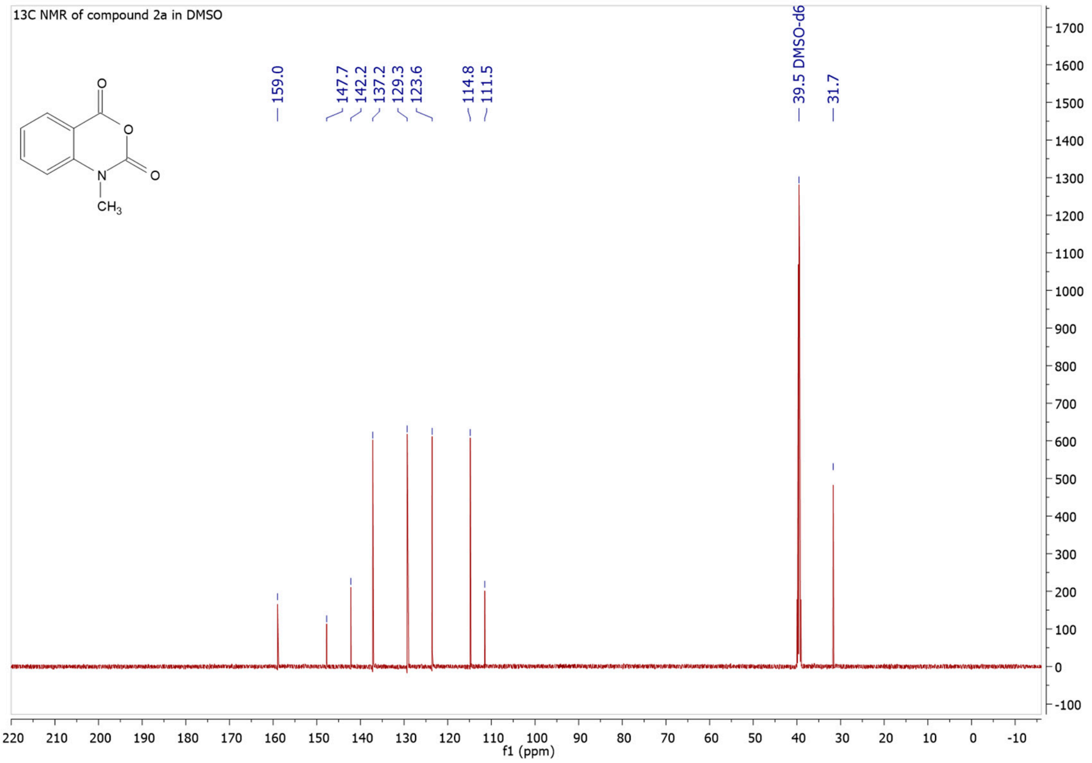

Figure S2. ^{13}C NMR spectrum of **2a**

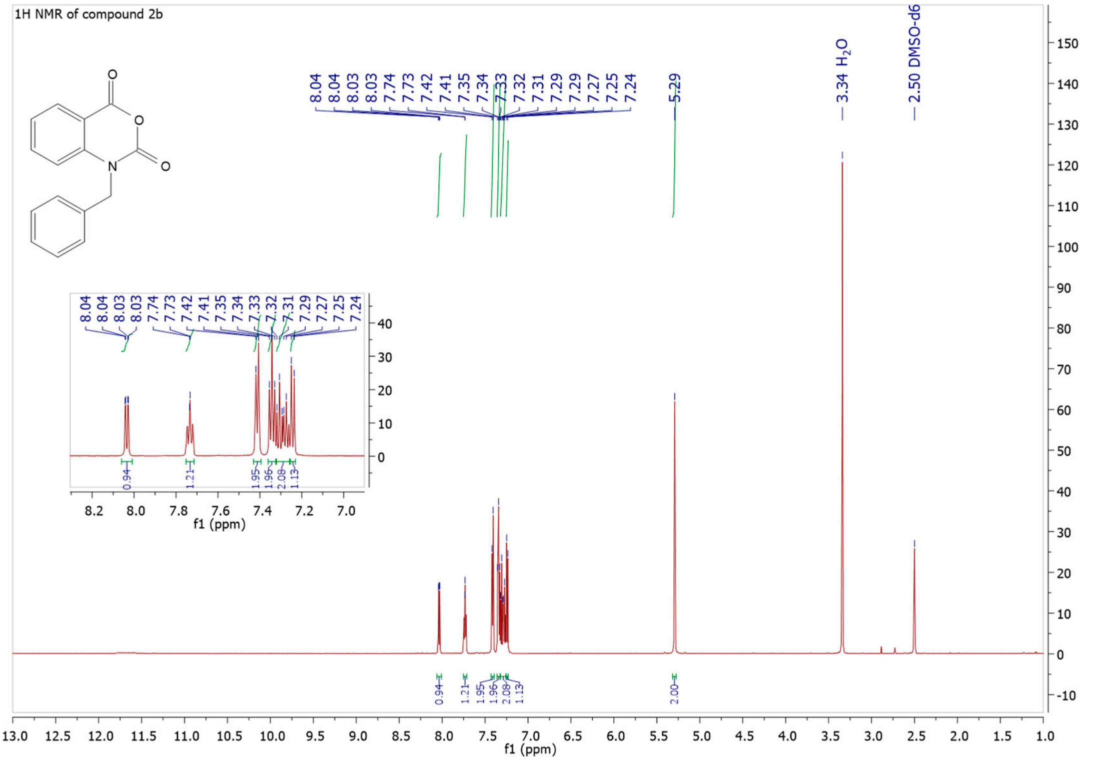

Figure S3. ^{1}H NMR spectrum of **2b**

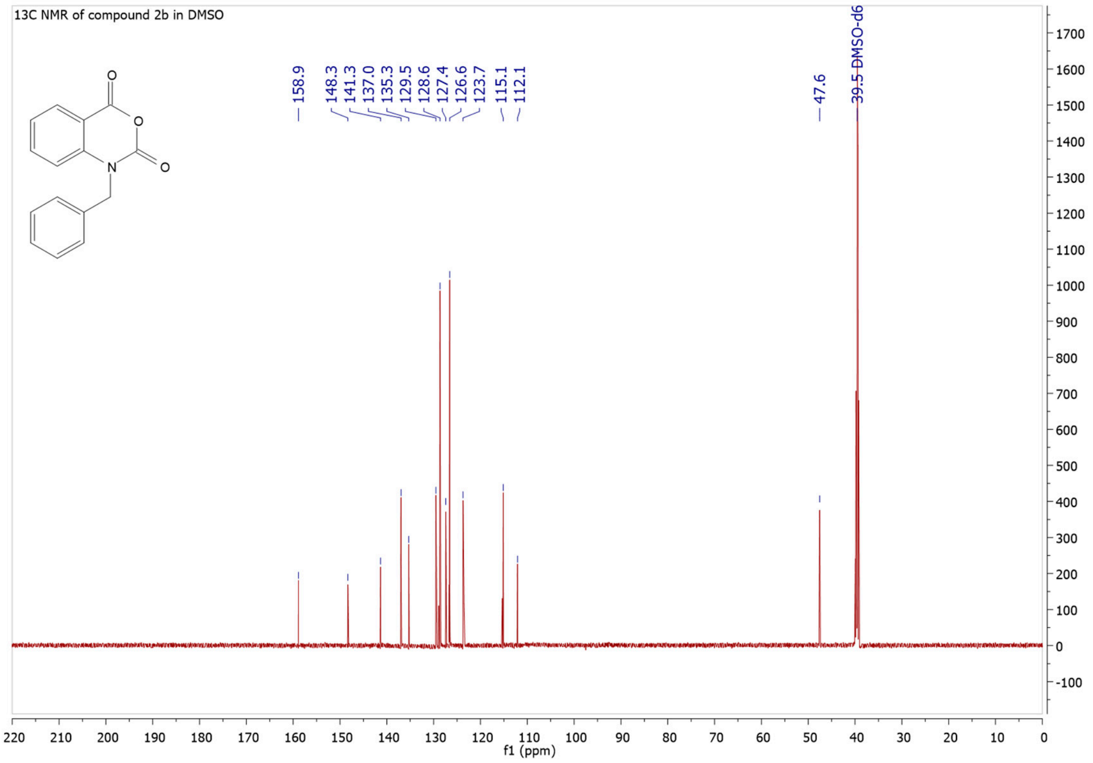

Figure S4. ^{13}C NMR spectrum of **2b**

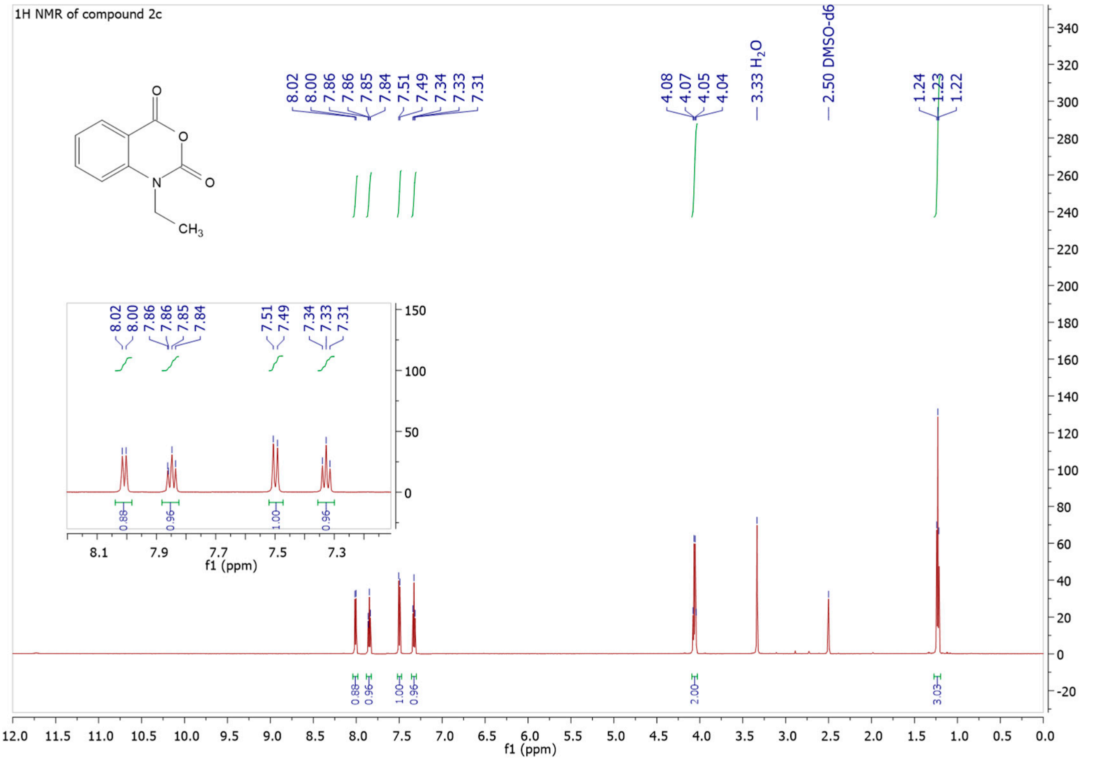

Figure S5. ^{1}H NMR spectrum of **2c**

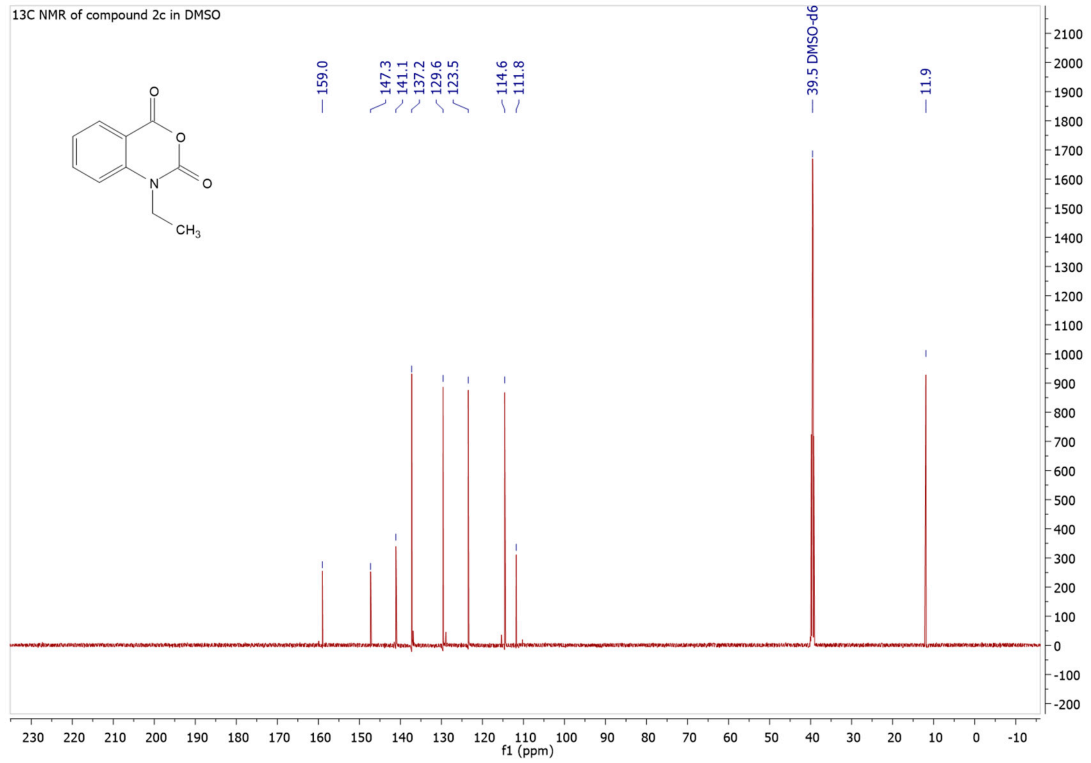

Figure S6. ^{13}C NMR spectrum of **2c**

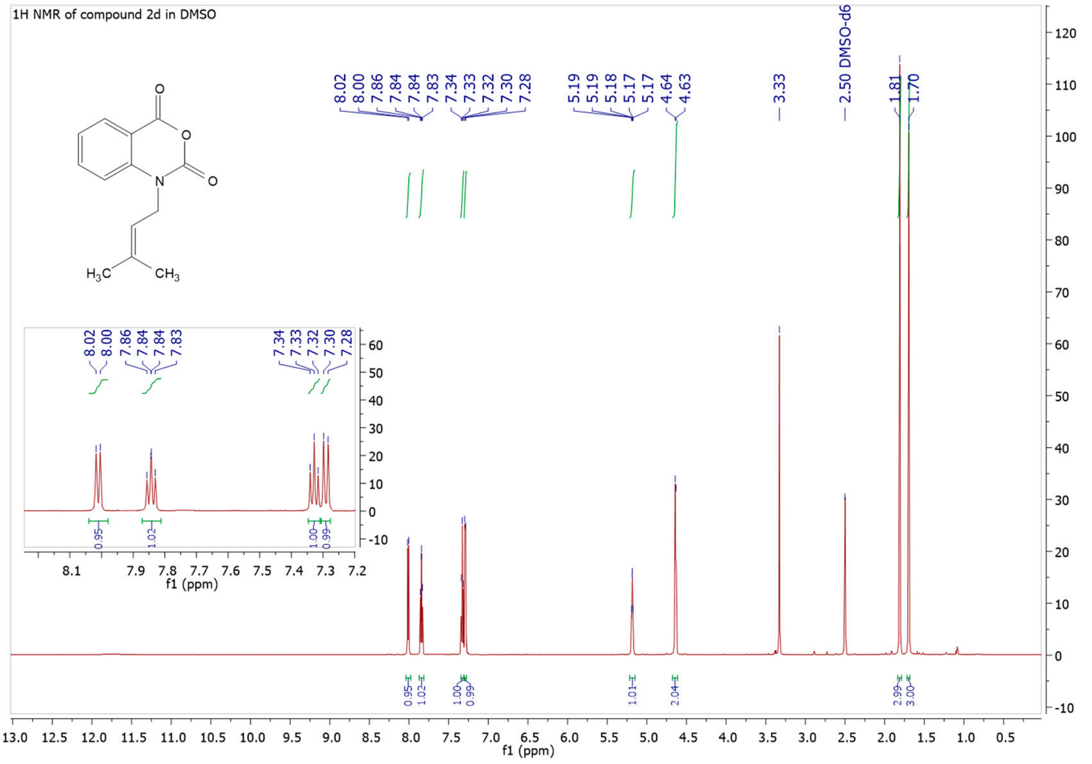

Figure S7. ^{1}H NMR spectrum of **2d**

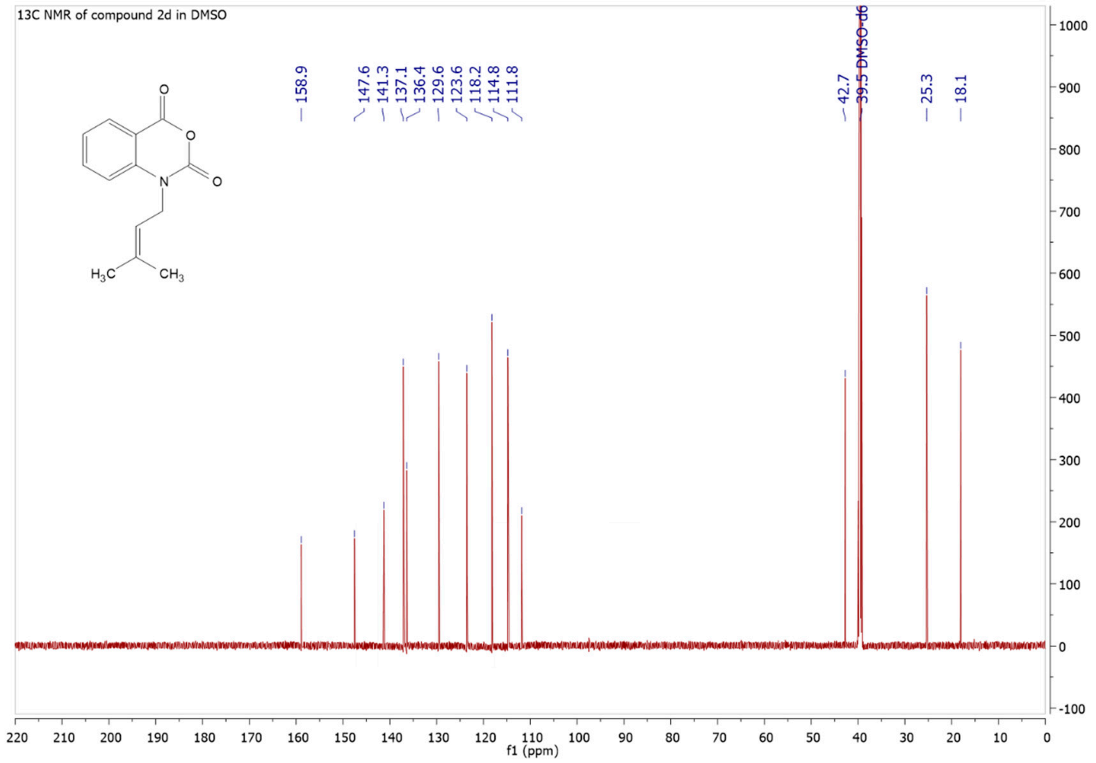

Figure S8. ^{13}C NMR spectrum of **2d**

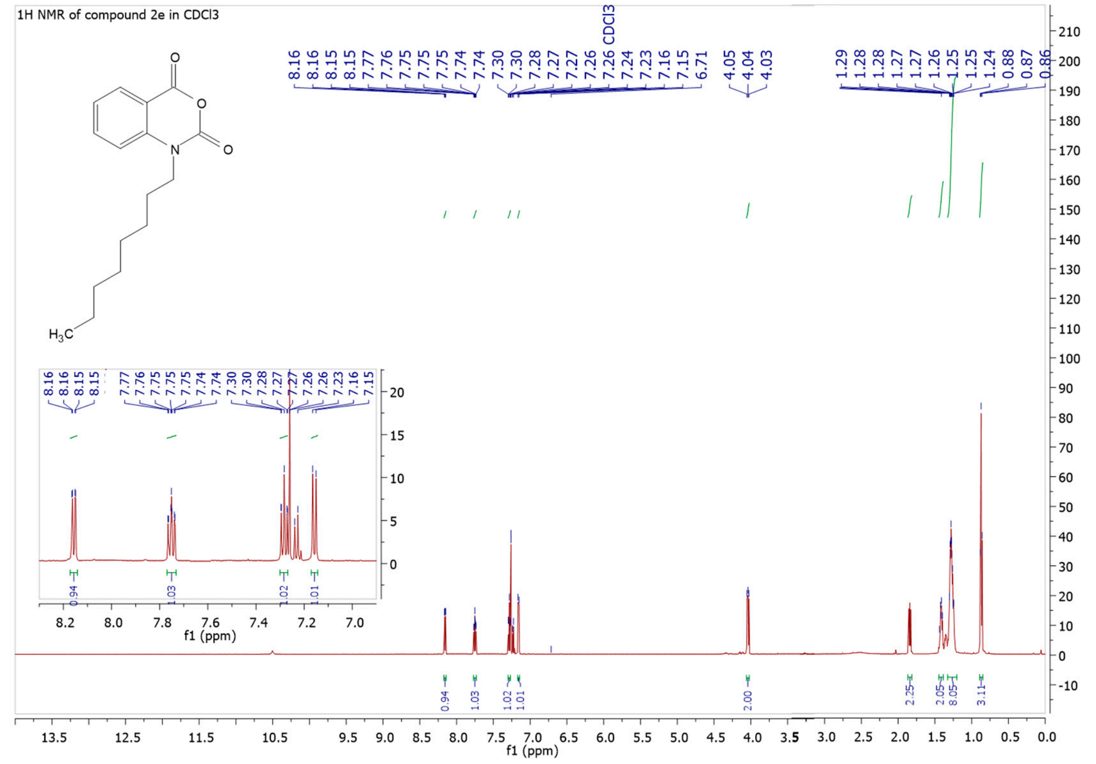

Figure S9. ^{1}H NMR spectrum of **2e**

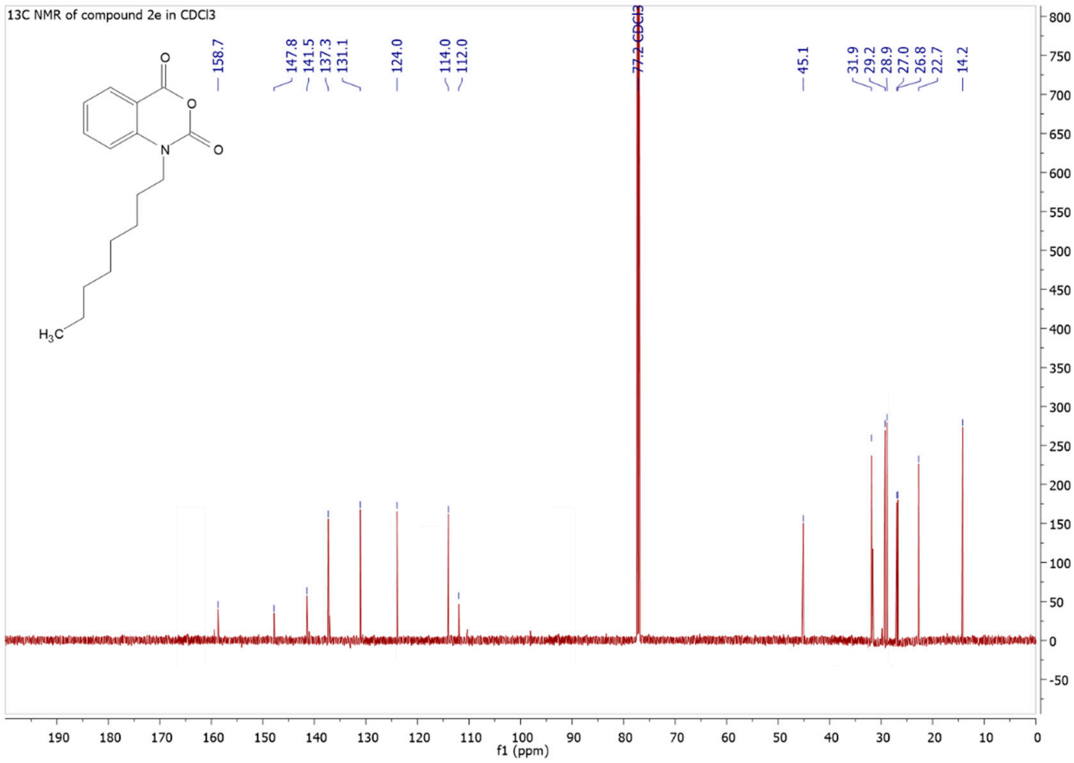

Figure S10. ^{13}C NMR spectrum of **2e**

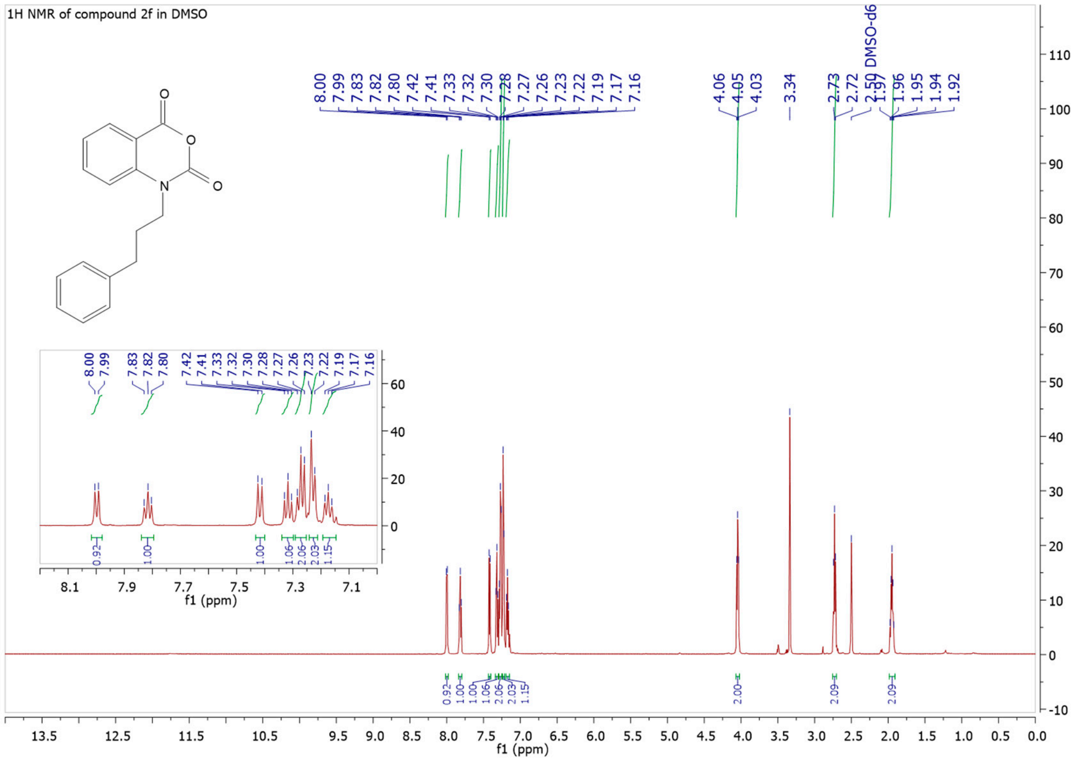

Figure S11. ^{1}H NMR spectrum of **2f**

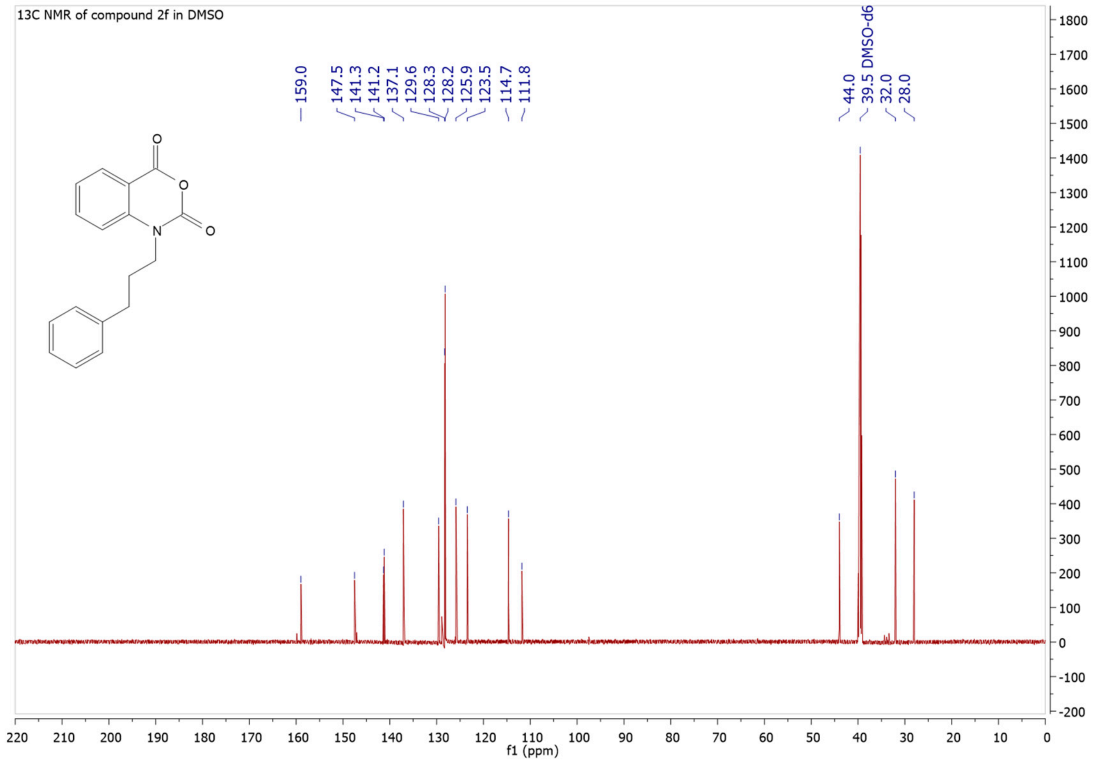

Figure S12. ^{13}C NMR spectrum of **2f**

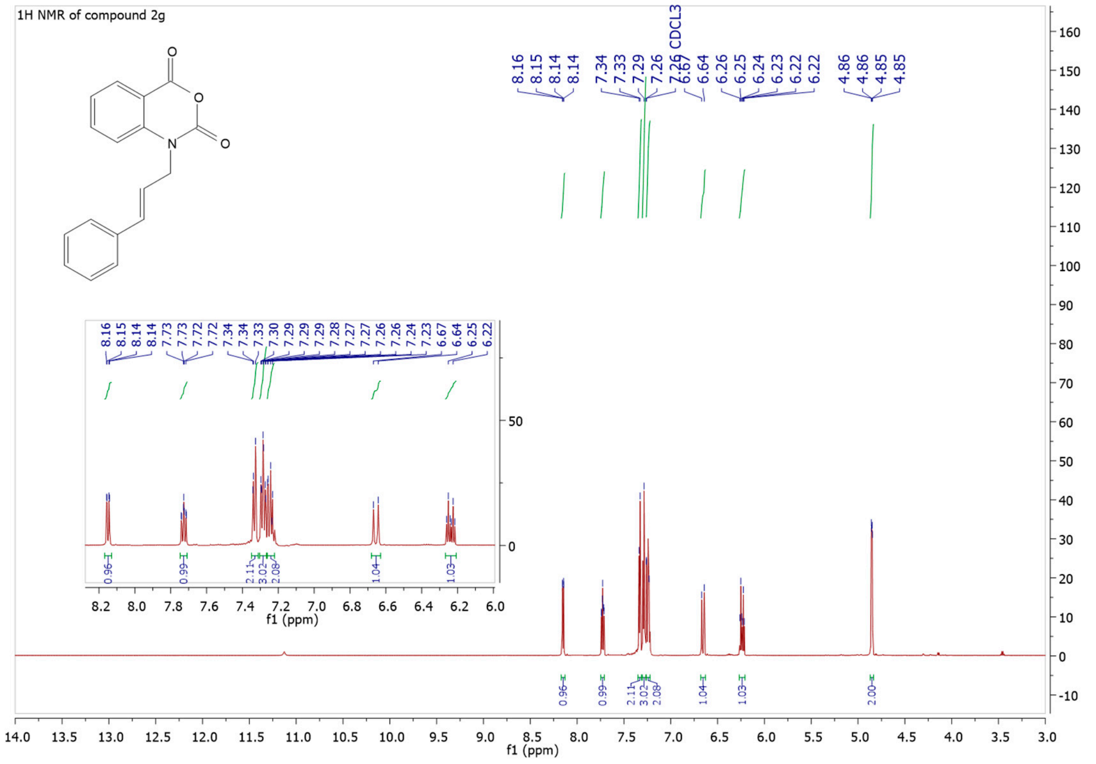

Figure S13. ^{1}H NMR spectrum of **2g**

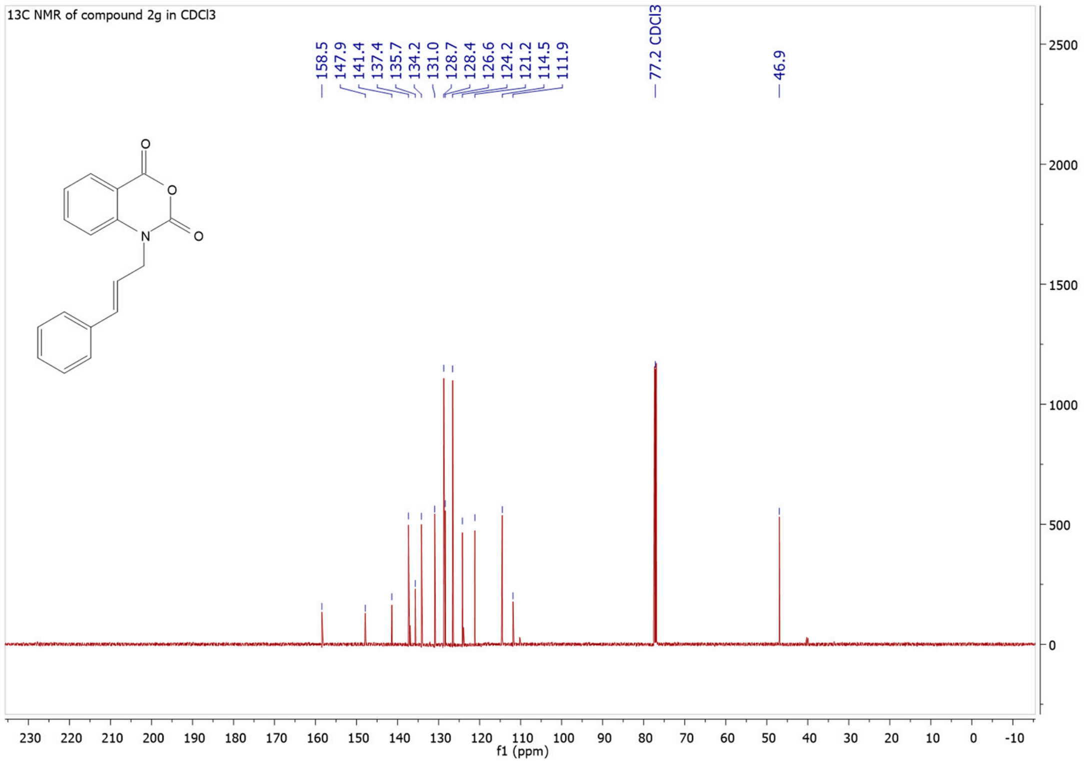

Figure S14. ^{13}C NMR spectrum of **2g**

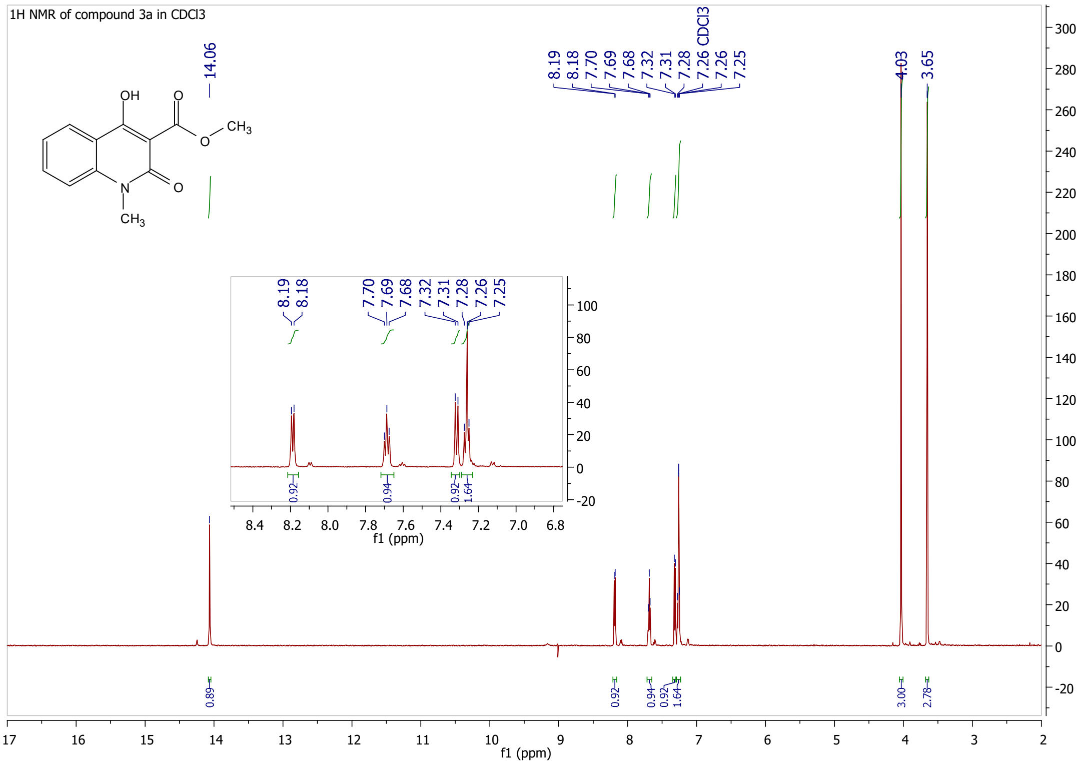

Figure S15. ^{1}H NMR spectrum of **3a**

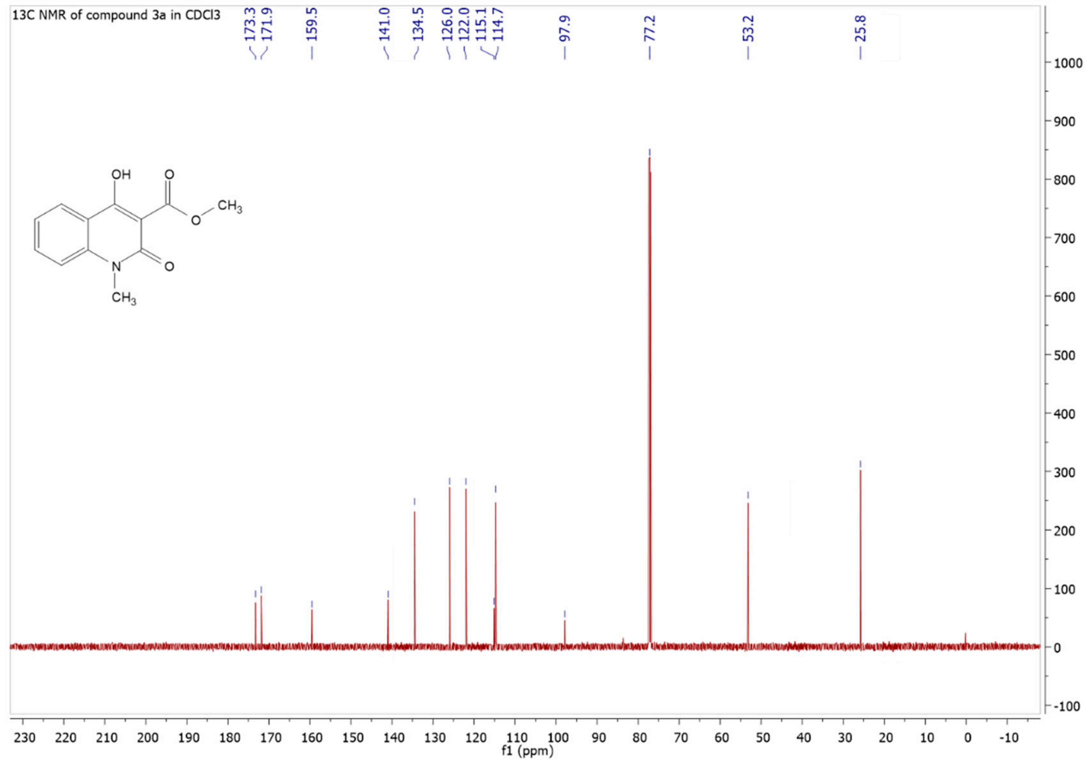

Figure S16. ^{13}C NMR spectrum of **3a**

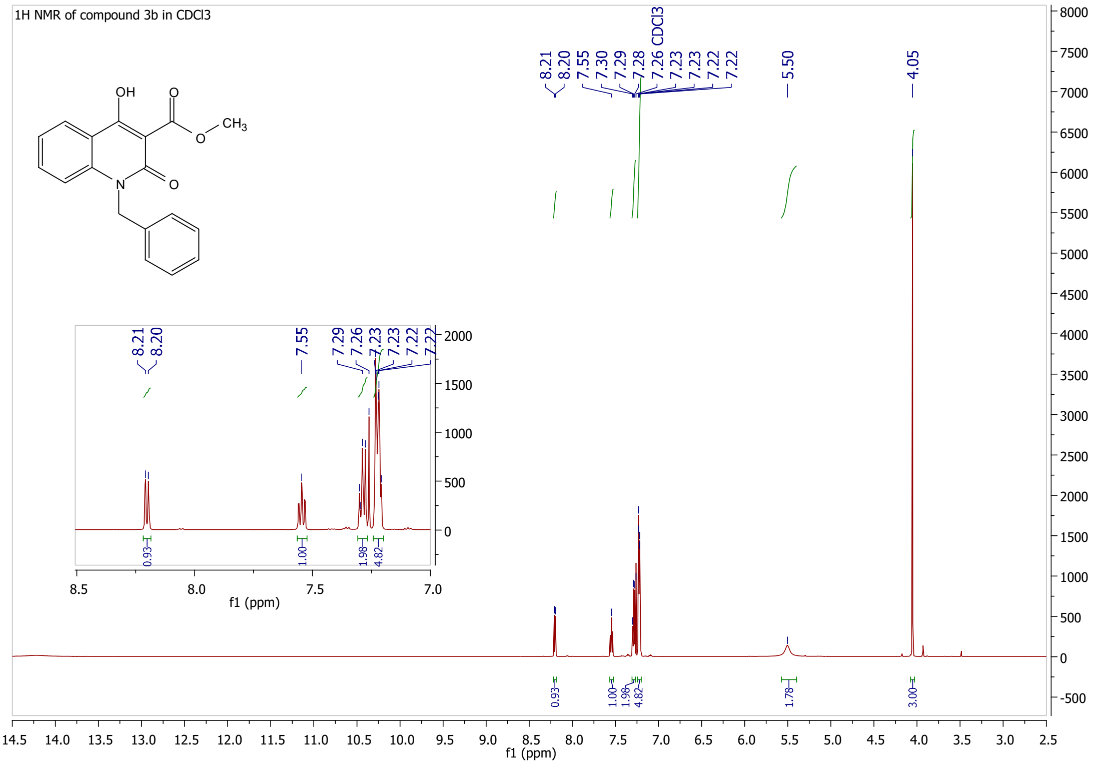

Figure S17. ^{1}H NMR spectrum of **3b**

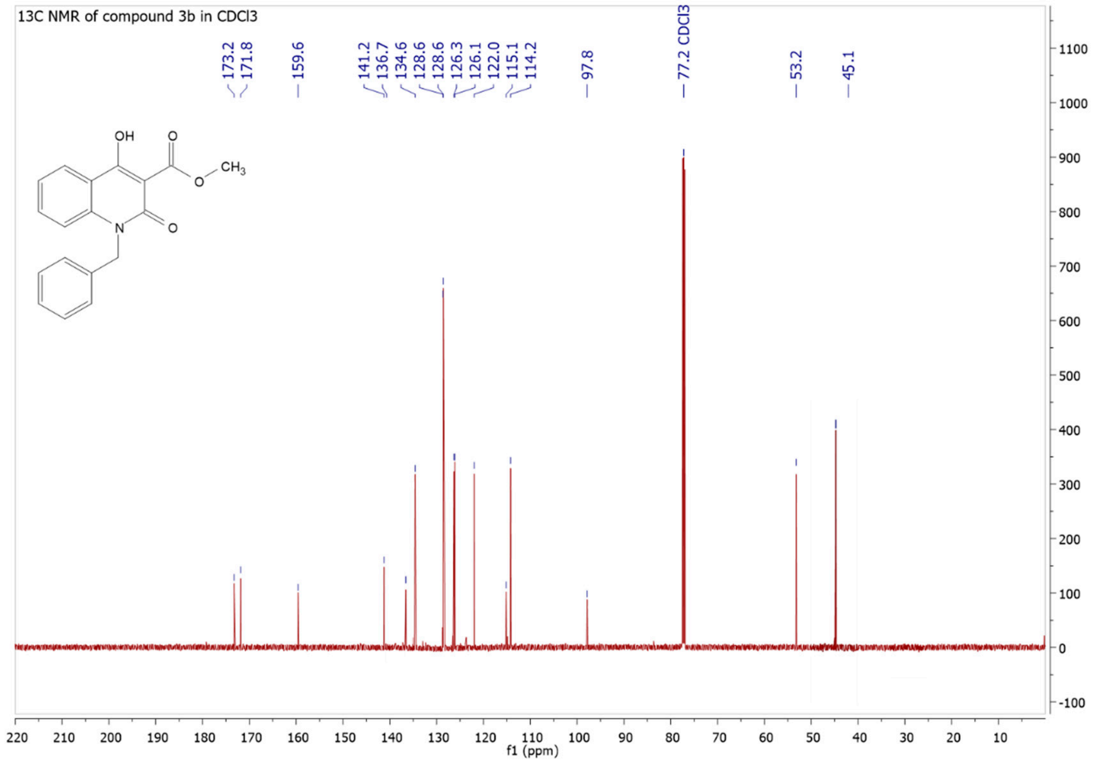

Figure S18. ^{13}C NMR spectrum of **3b**

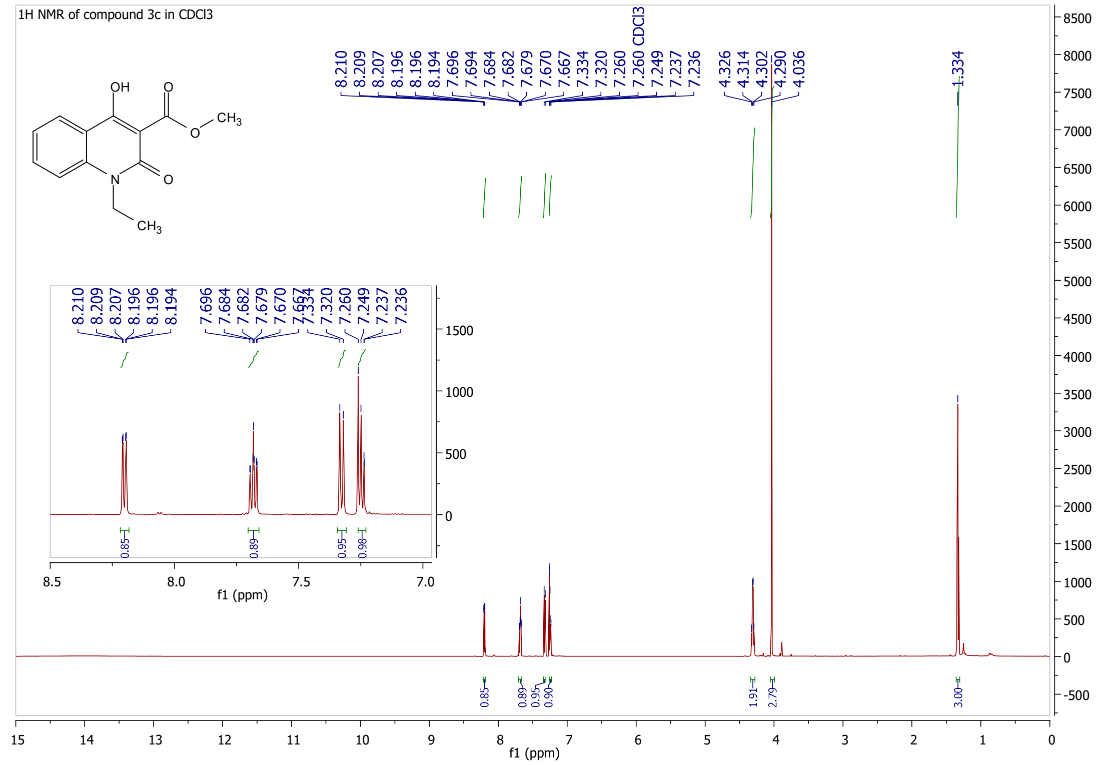

Figure S19. ^{1}H NMR spectrum of **3c**

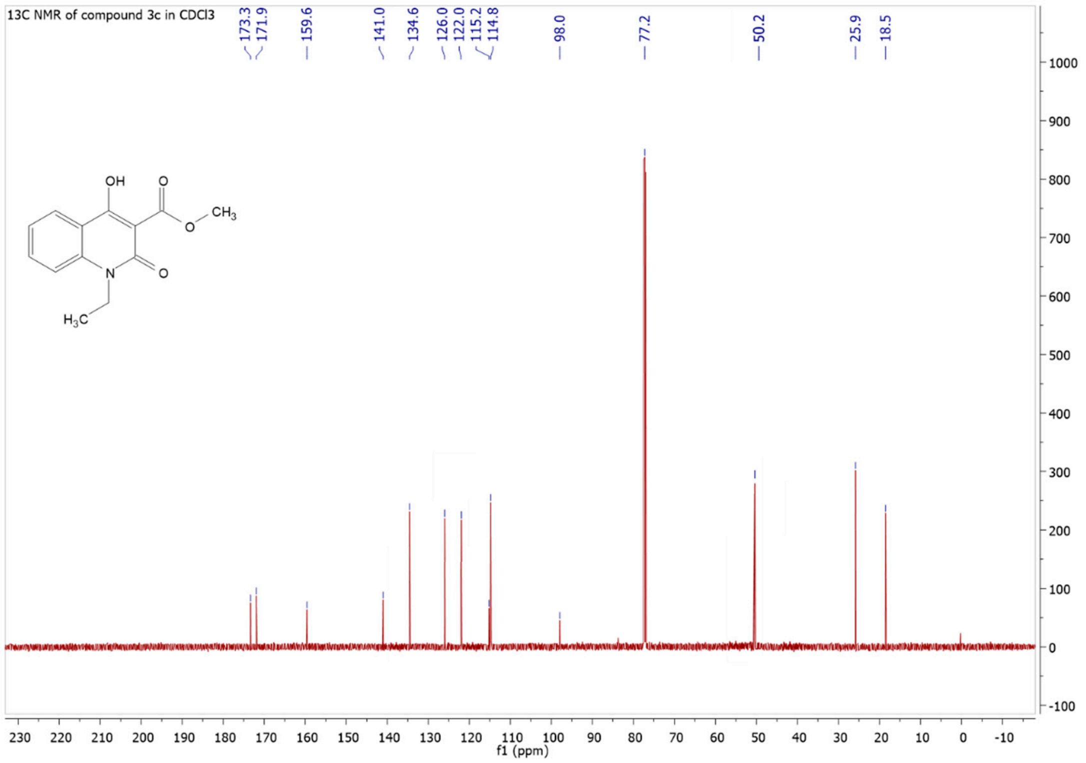

Figure S20. ^{13}C NMR spectrum of **3c**

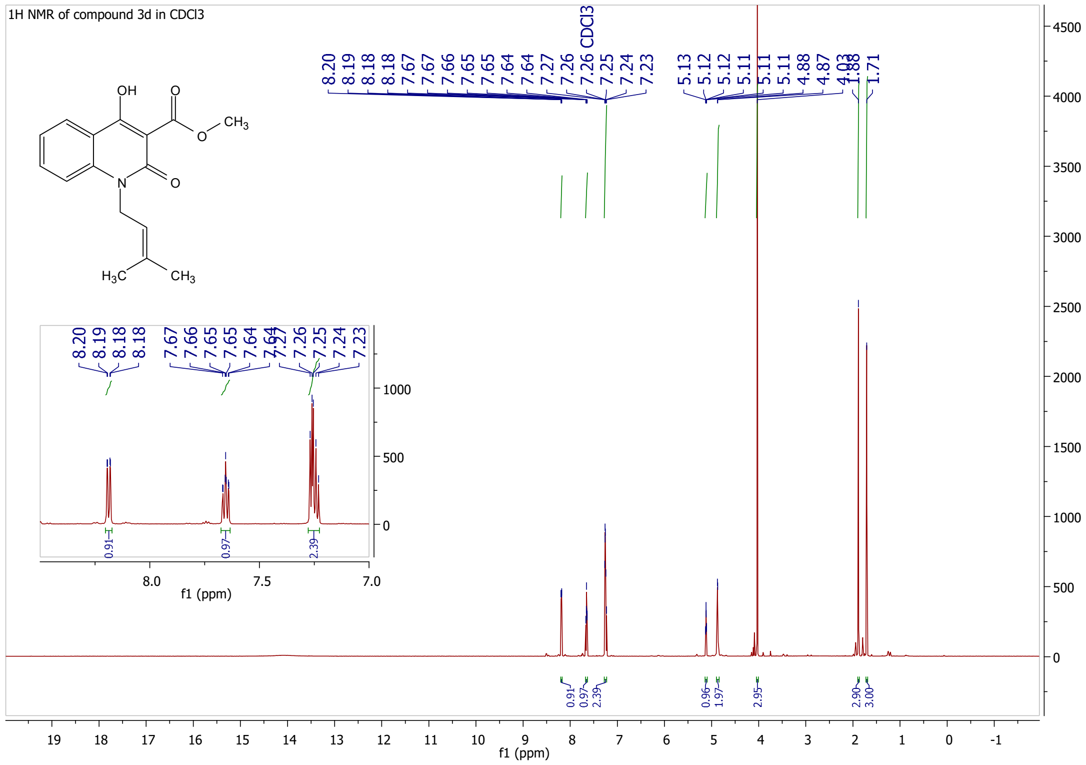

Figure S21. ^{1}H NMR spectrum of **3d**

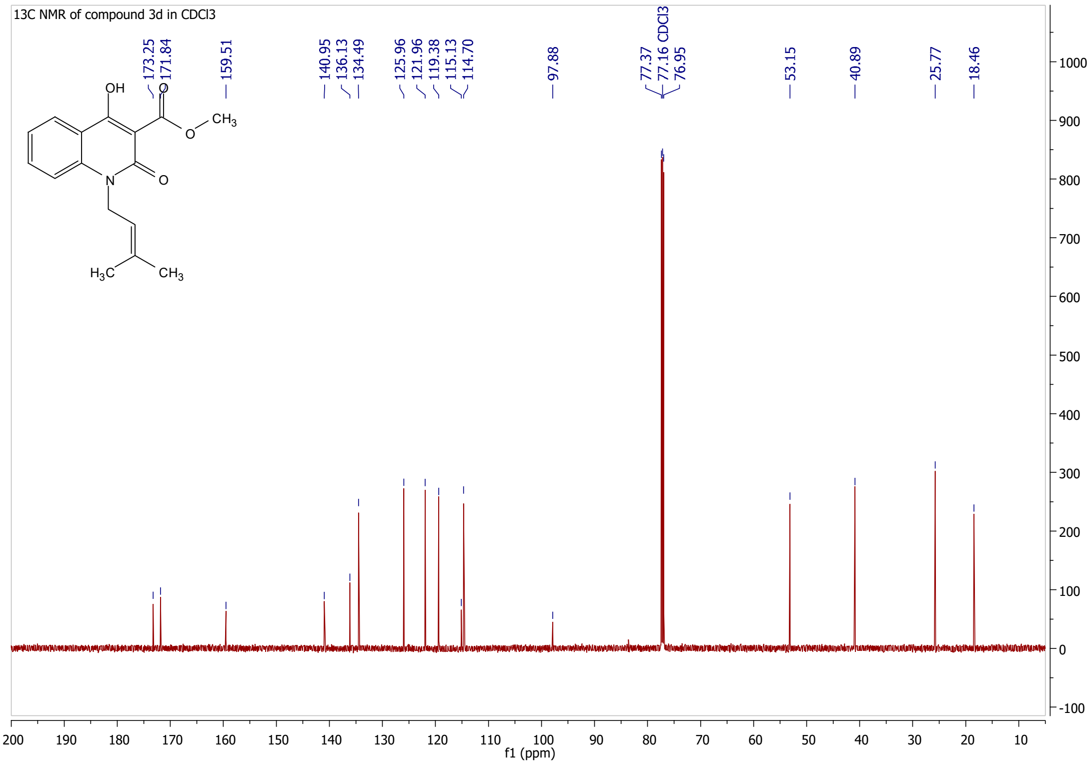

Figure S22. ^{13}C NMR spectrum of **3d**

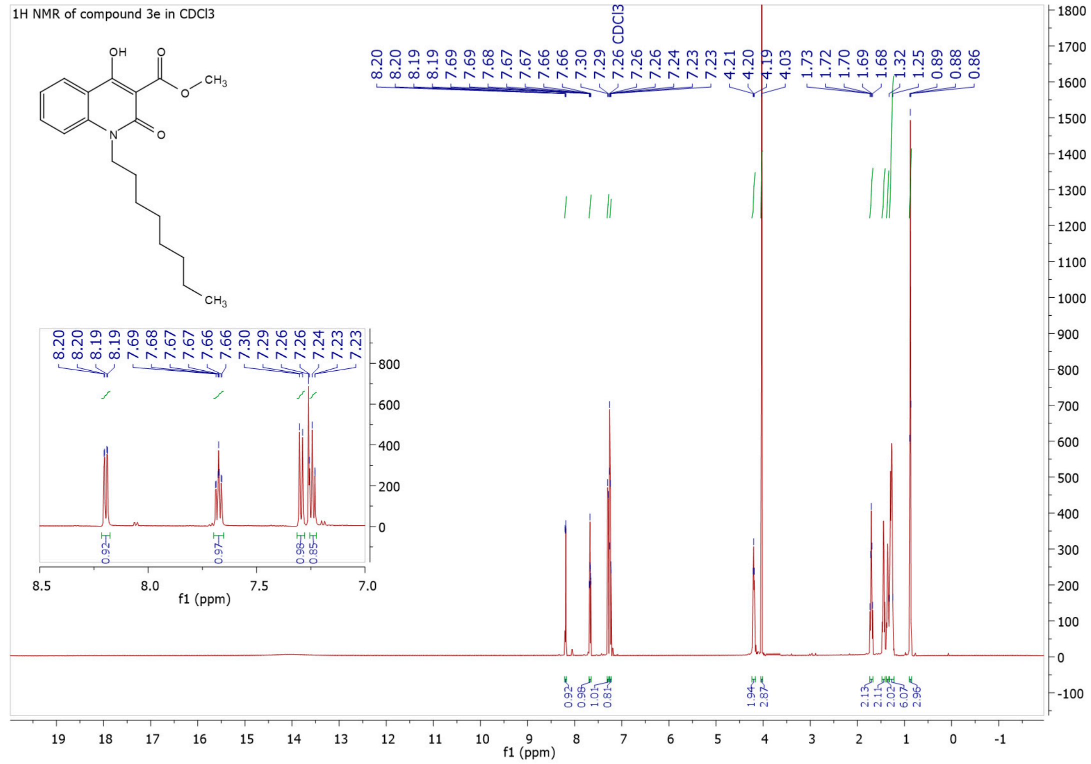

Figure S23. ^{1}H NMR spectrum of **3e**

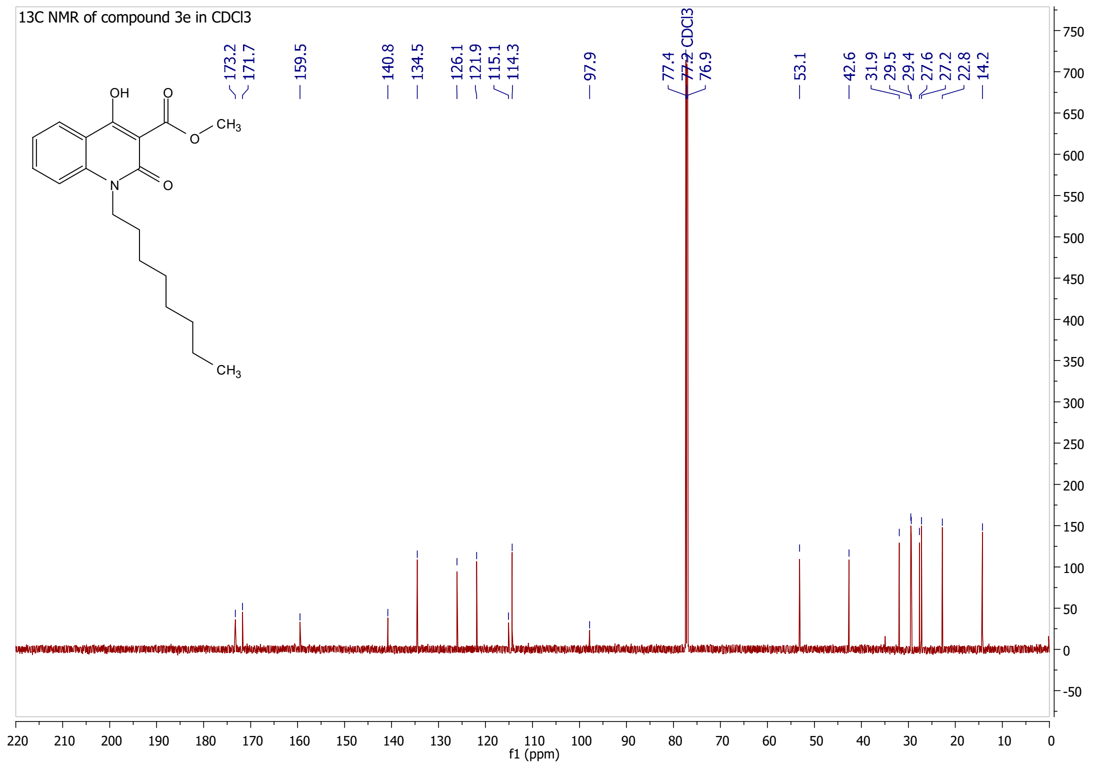

Figure S24. ^{13}C NMR spectrum of **3e**

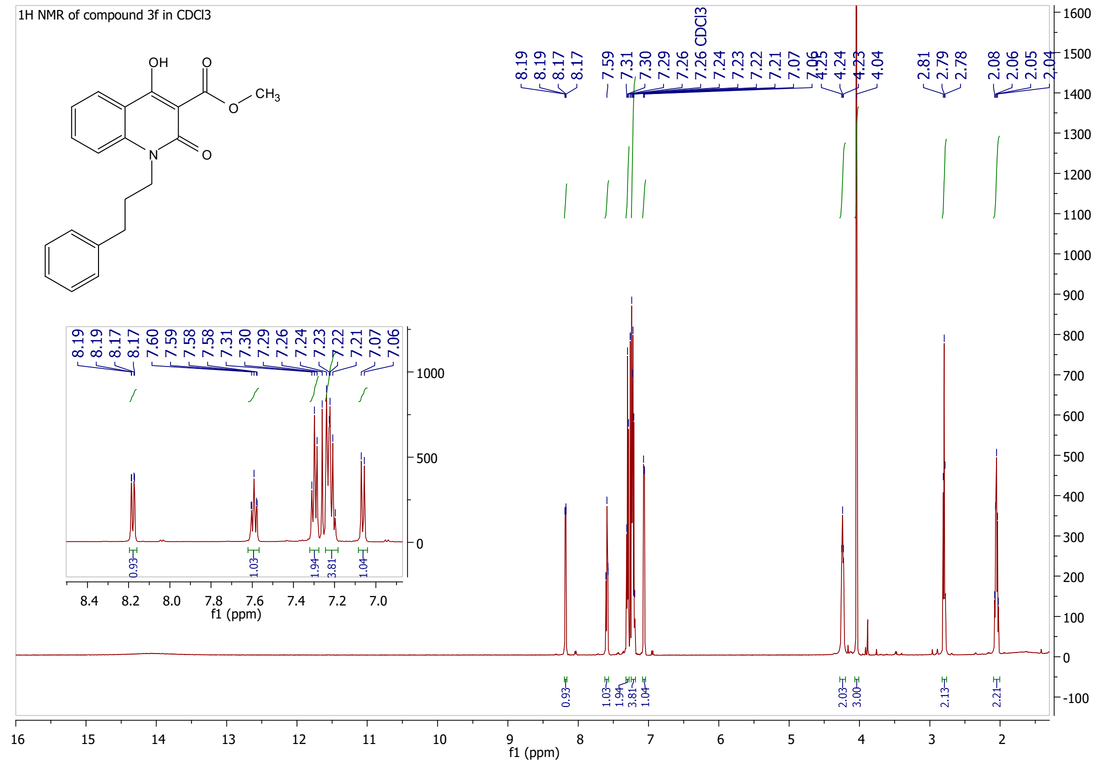

Figure S25. ^{1}H NMR spectrum of **3f**

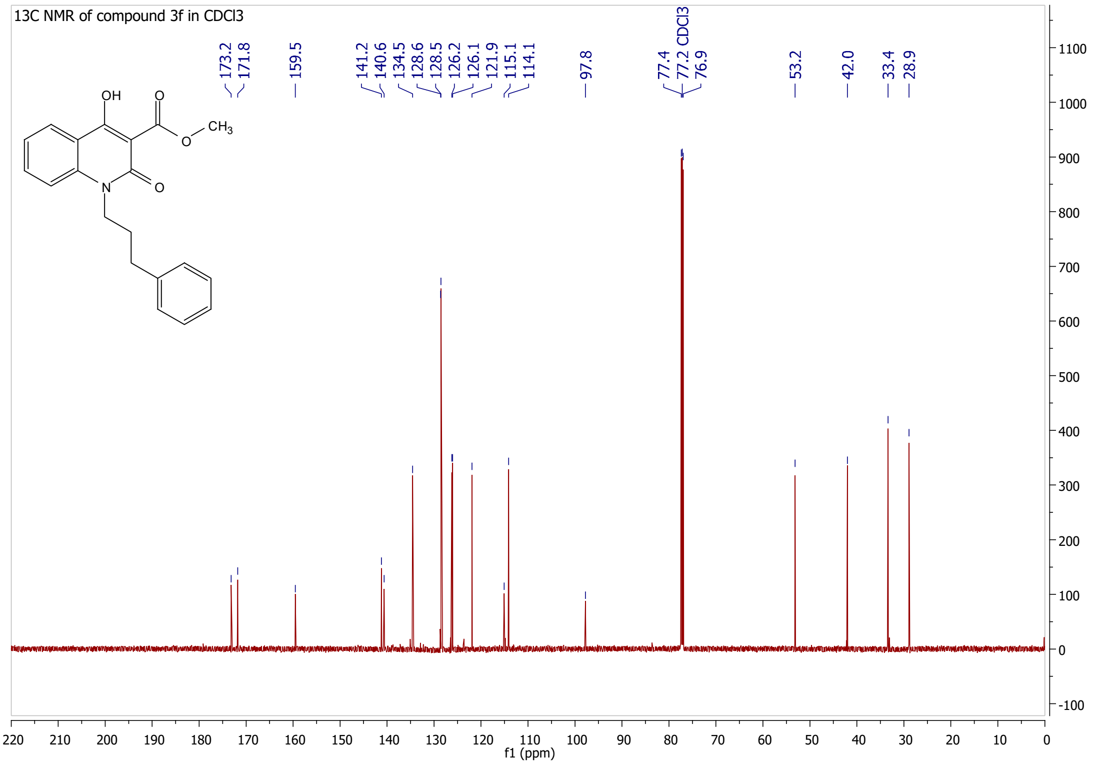

Figure S26. ^{13}C NMR spectrum of **3f**

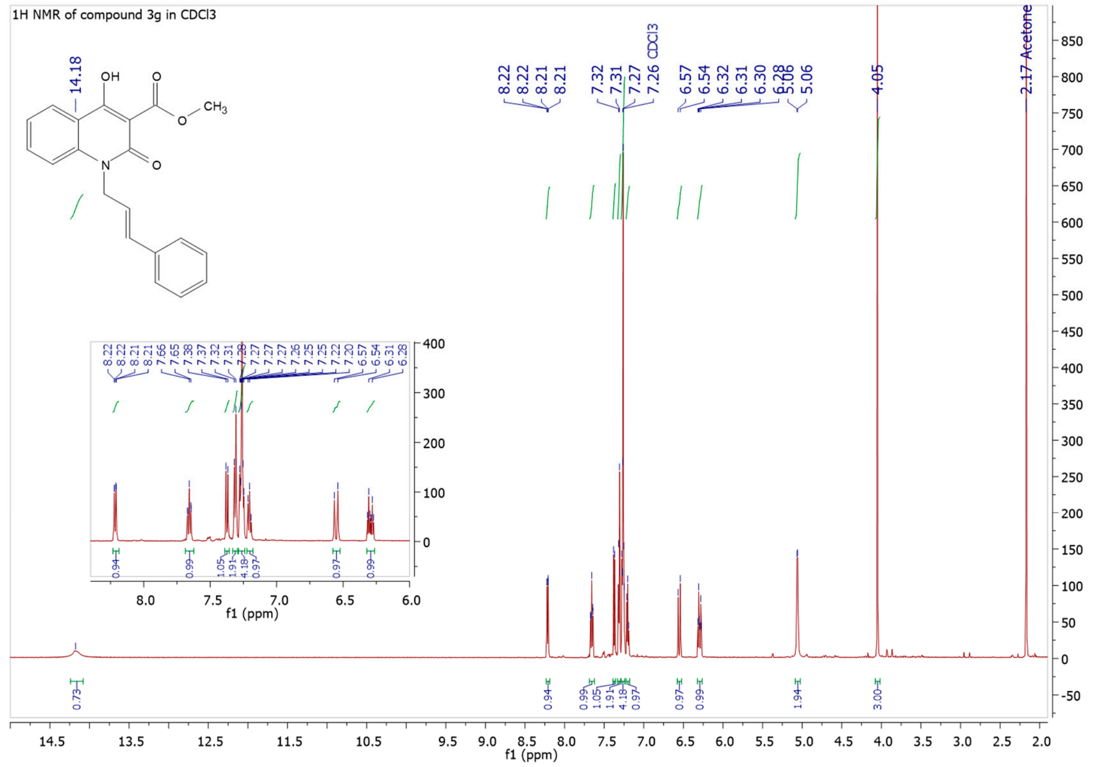

Figure S27. ^{1}H NMR spectrum of **3g**

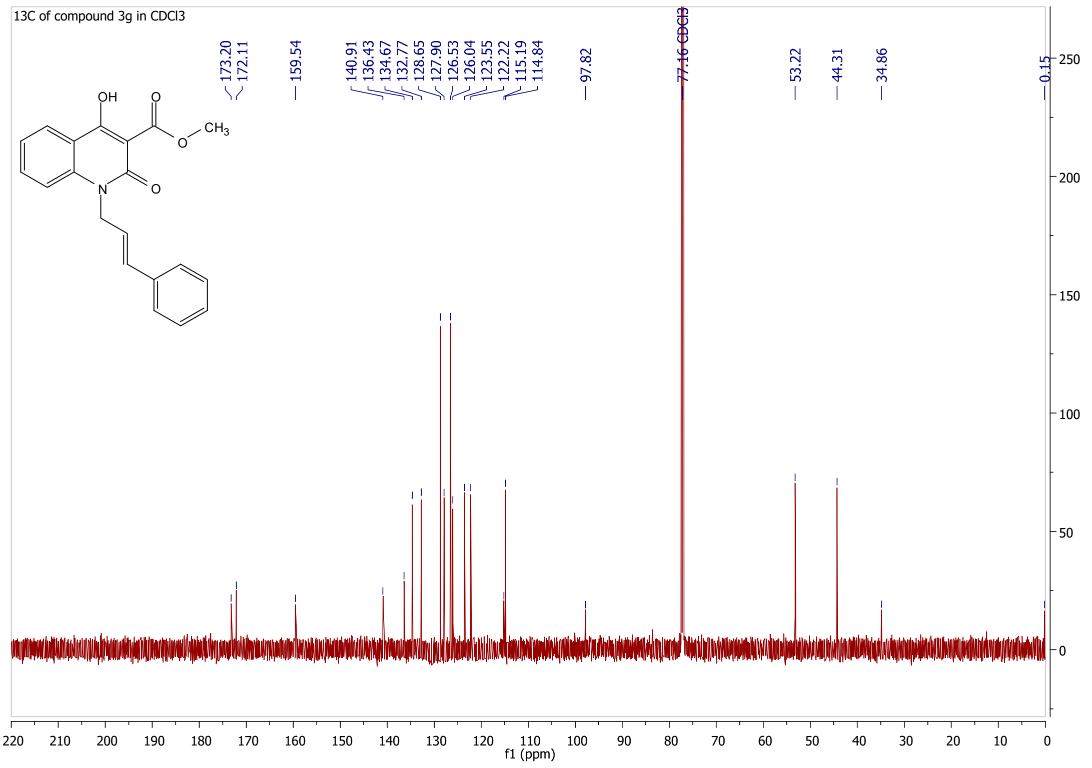

Figure S28. ^{13}C NMR spectrum of **3g**

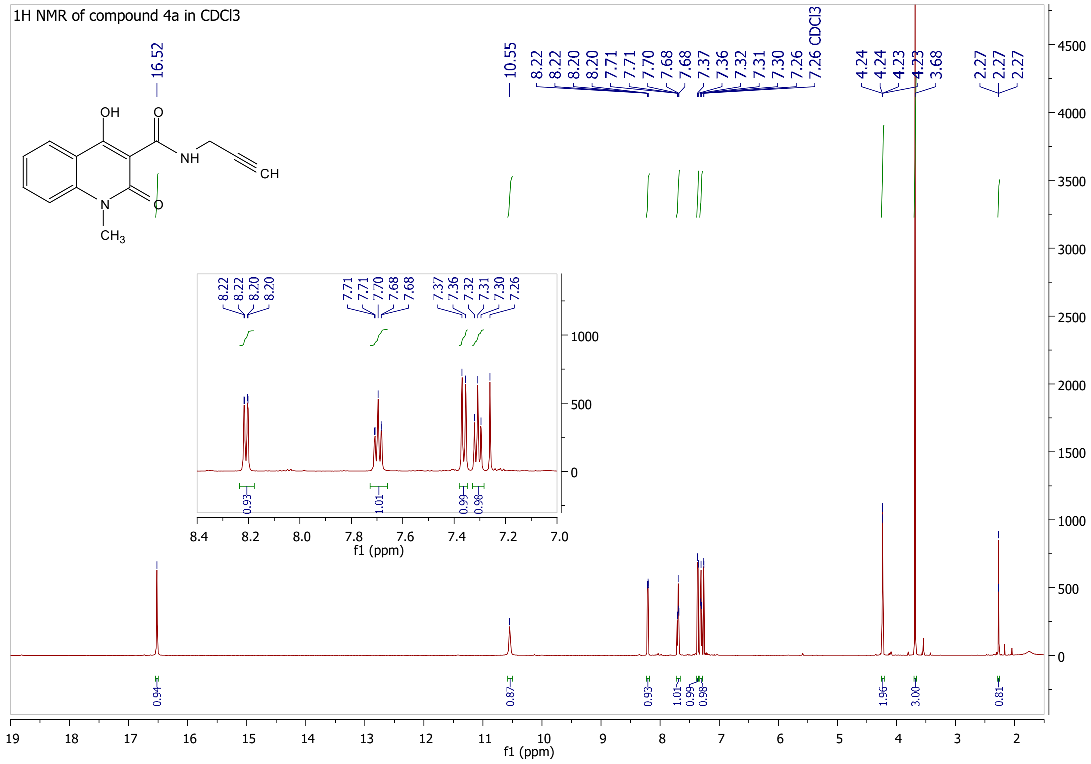

Figure S29. ^{1}H NMR spectrum of **4a**

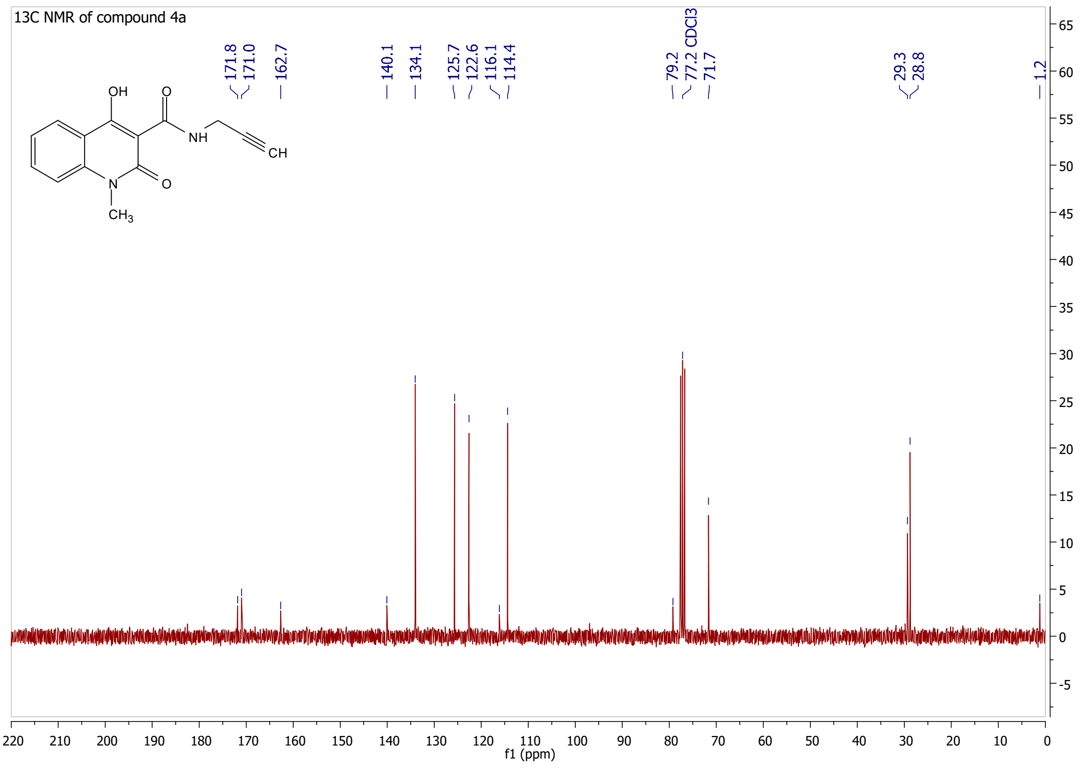

Figure S30. ^{13}C NMR spectrum of **4a**

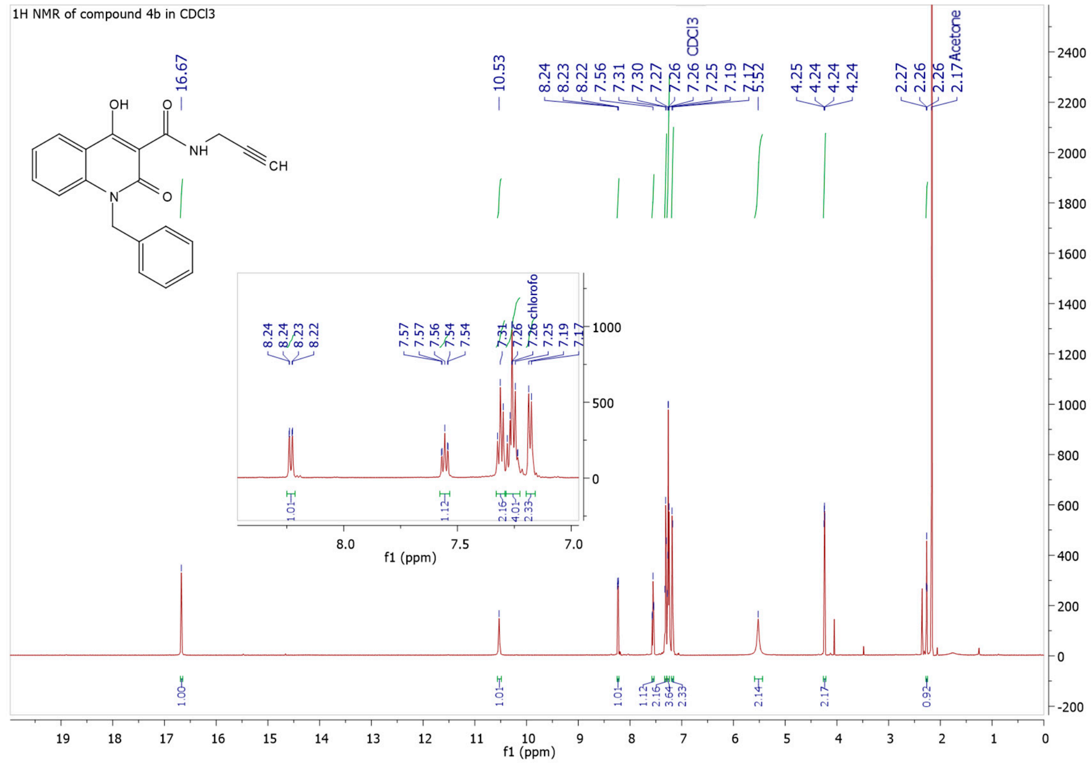

Figure S31. ^{1}H NMR spectrum of **4b**

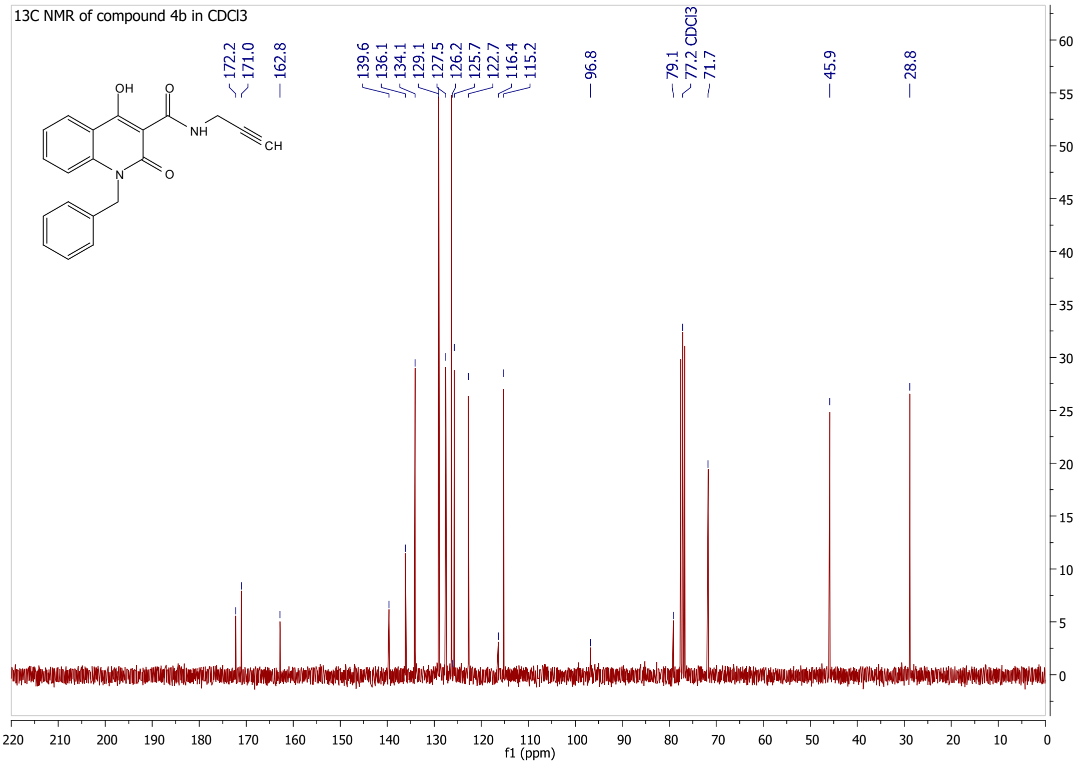

Figure S32. ${}^{13}C$ NMR of compound **4b**

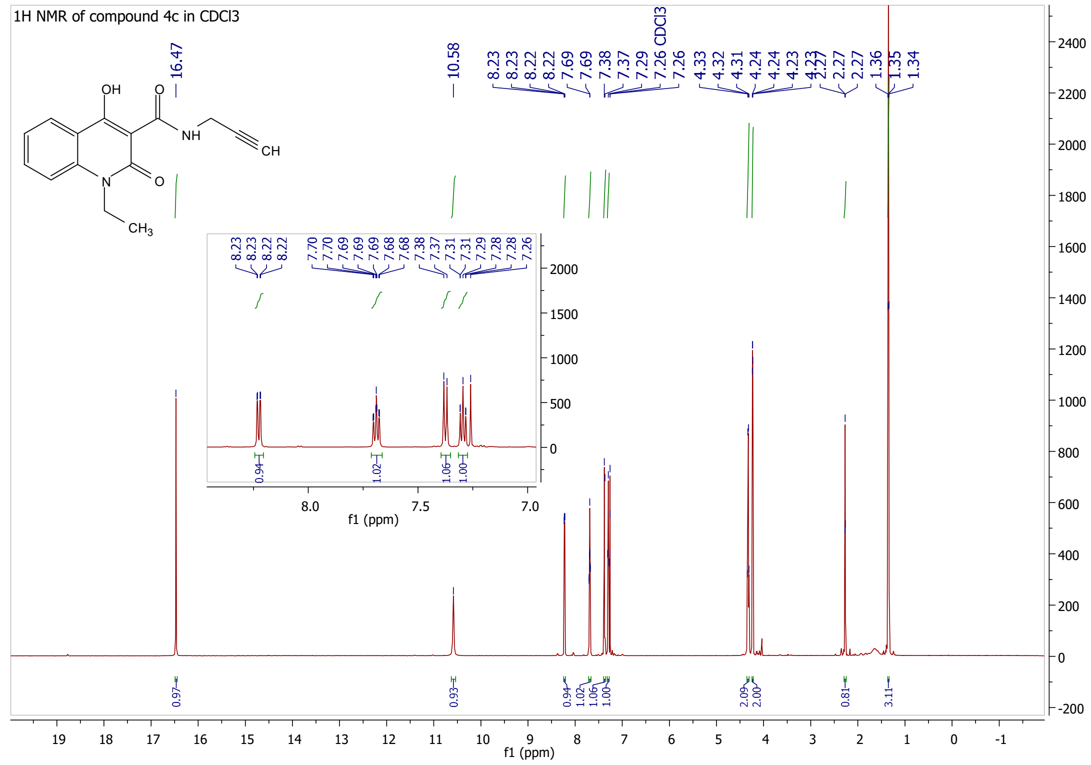

Figure S33. ${}^{1}H$ NMR of compound **4c**

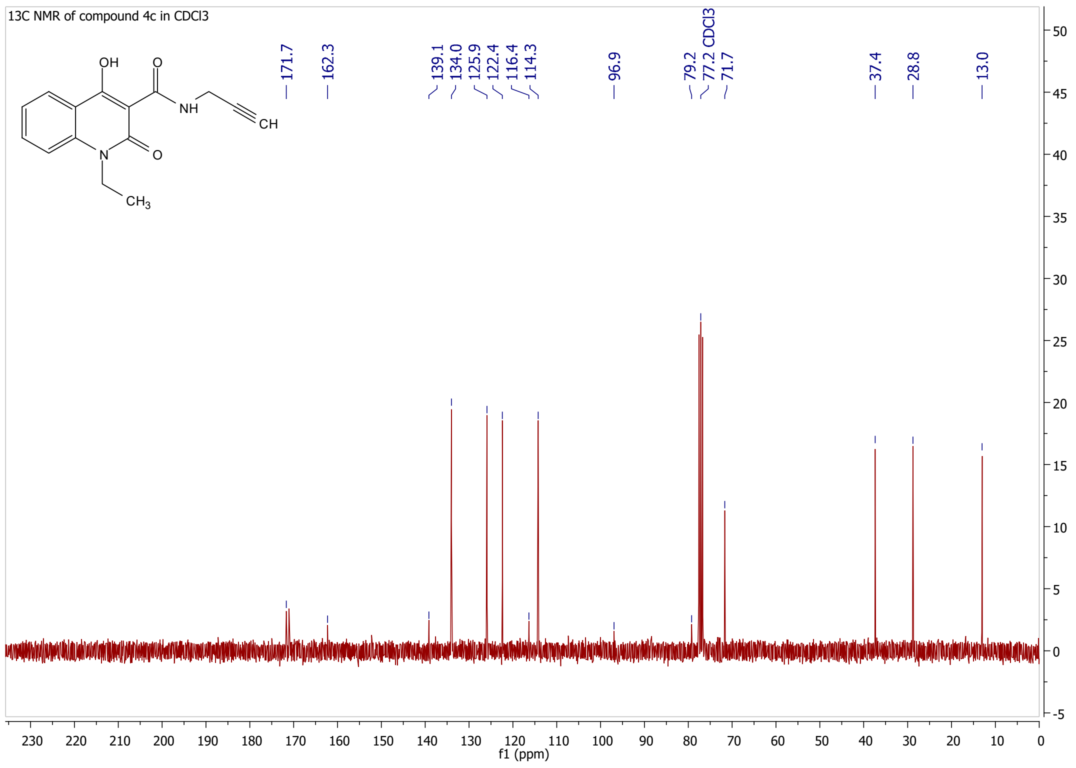

Figure S34. ^{13}C NMR of compound **4c**

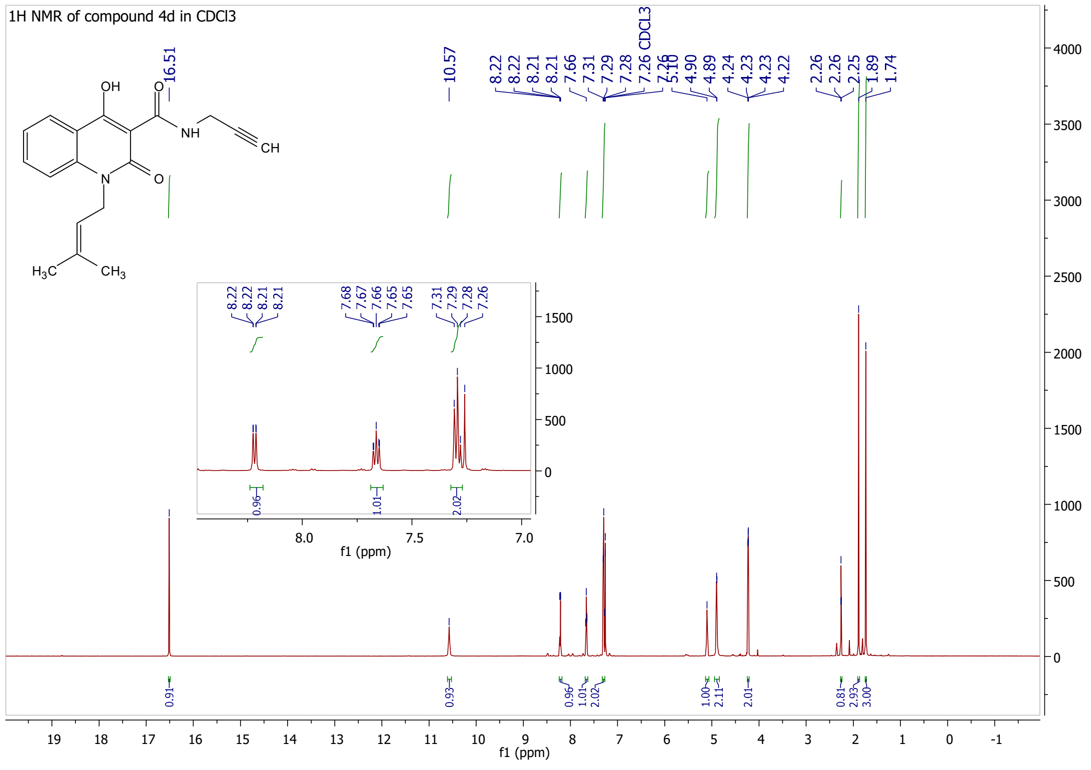

Figure S35. ^{1}H NMR of compound **4d**

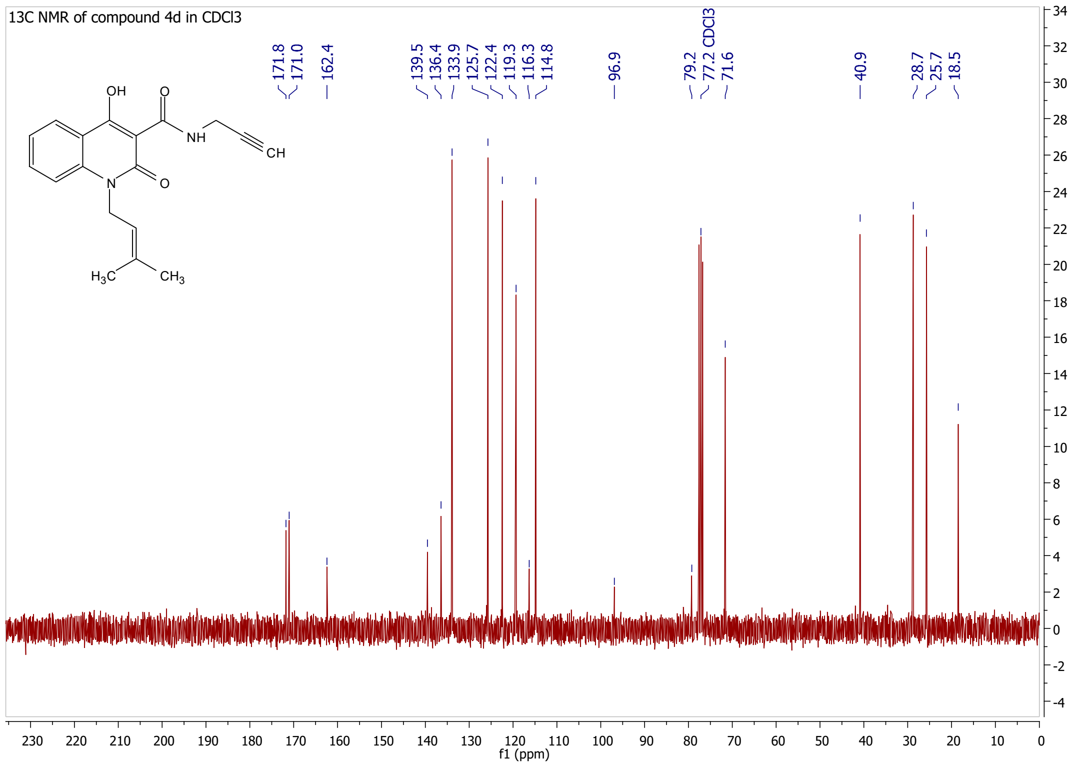

Figure S36. ^{13}C NMR of compound **4d**

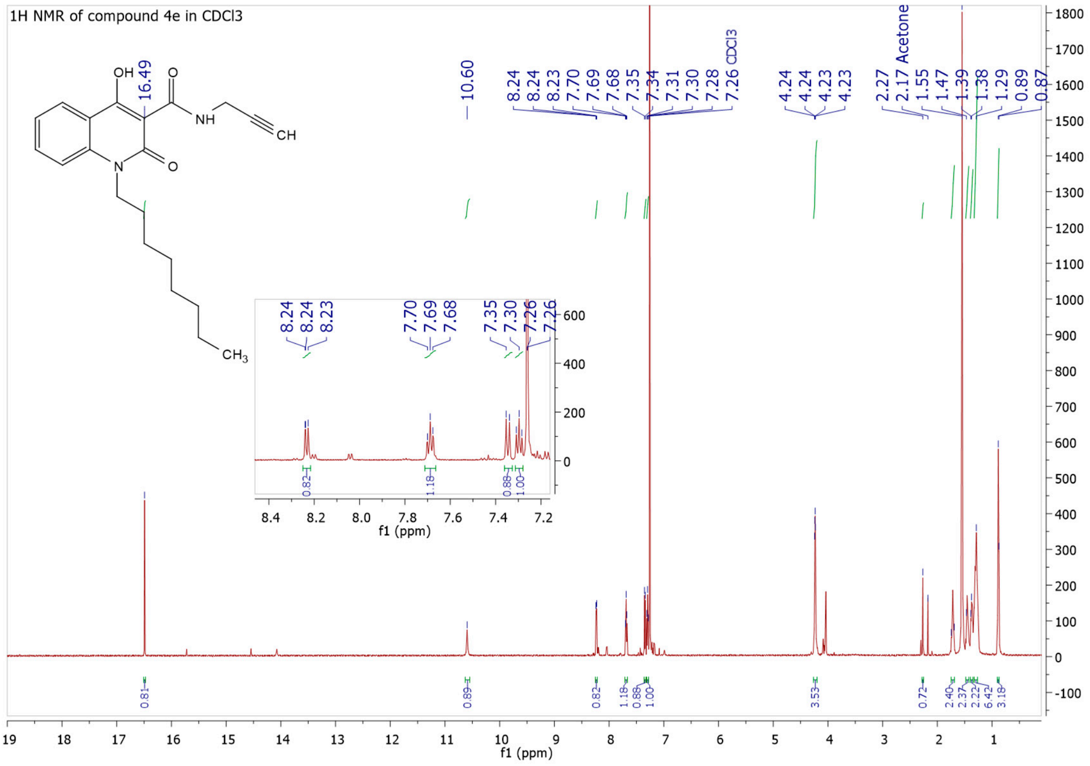

Figure S37. ^{1}H NMR of compound **4e**

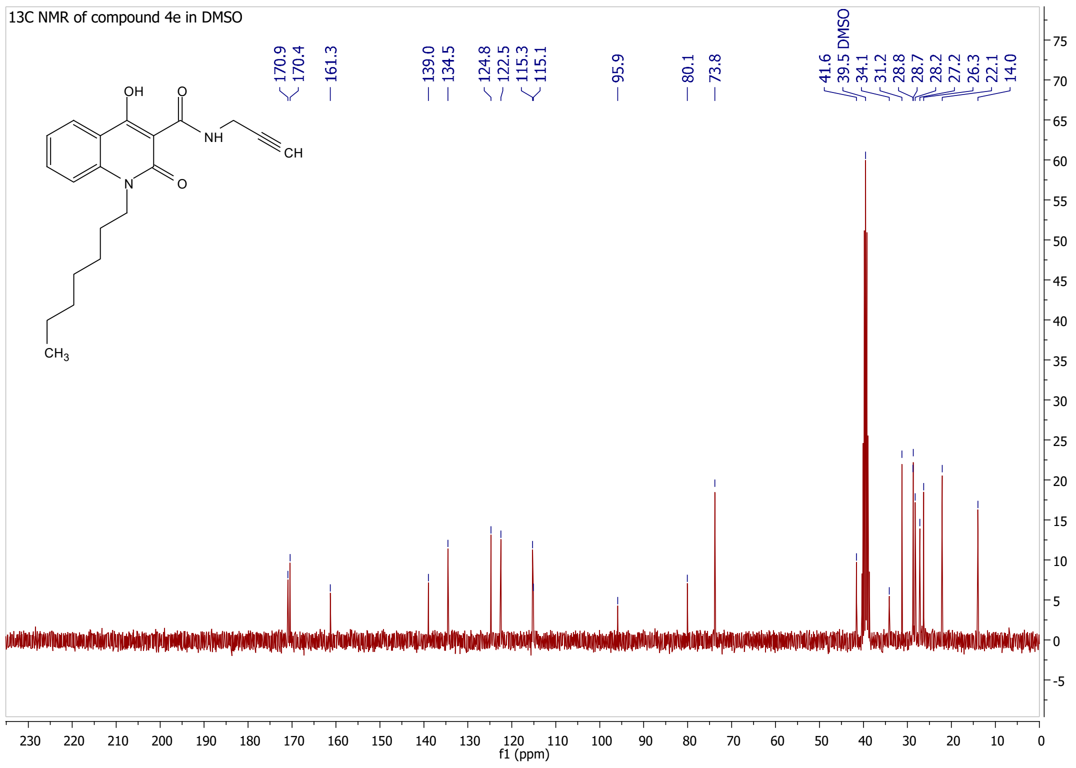

Figure S38. ^{13}C NMR of compound **4e**

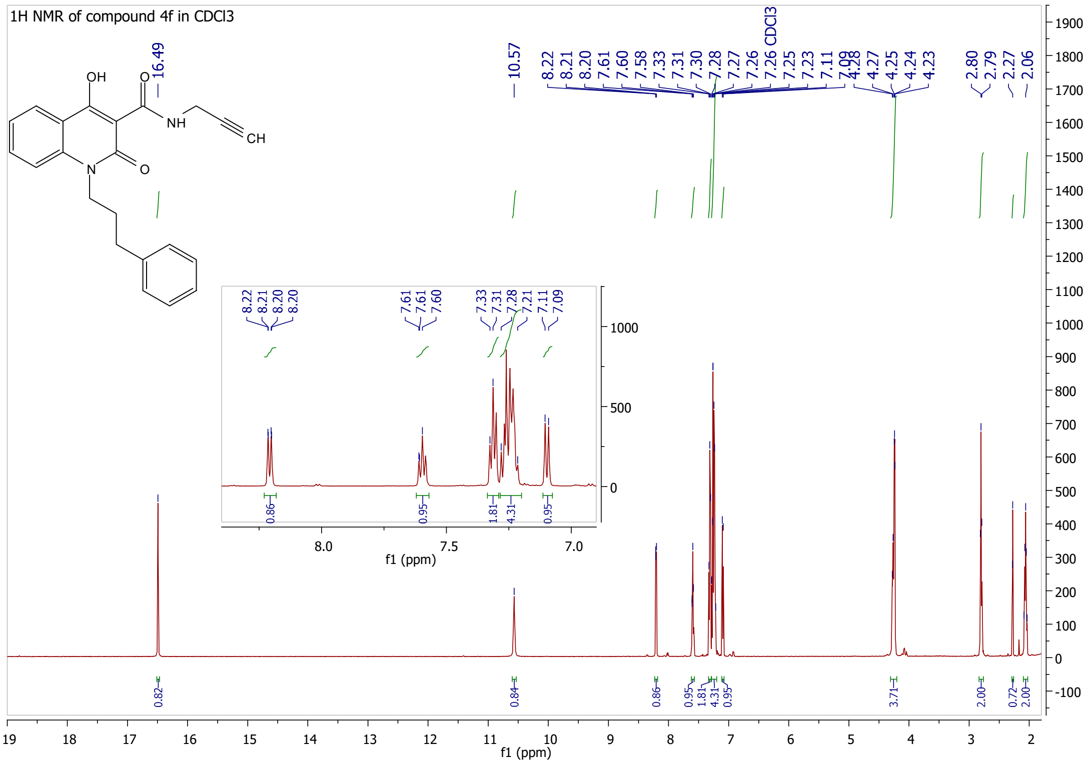

Figure S39. ^{1}H NMR of compound **4f**

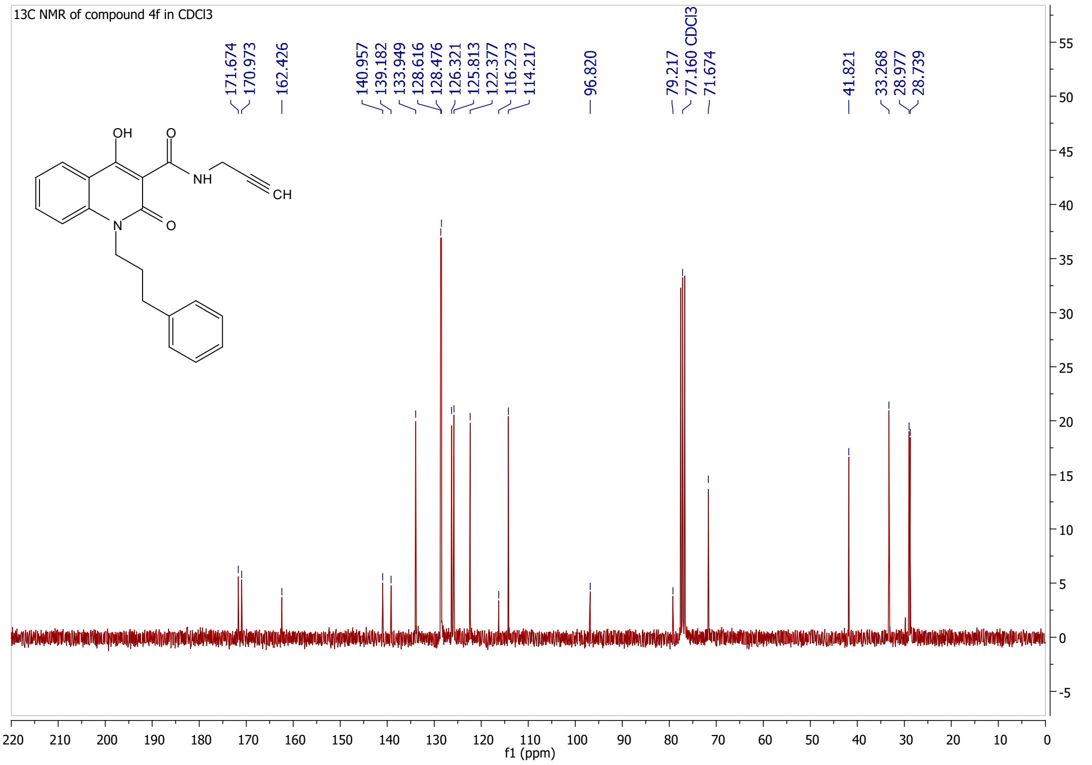

Figure S40. ^{13}C NMR of compound **4f**

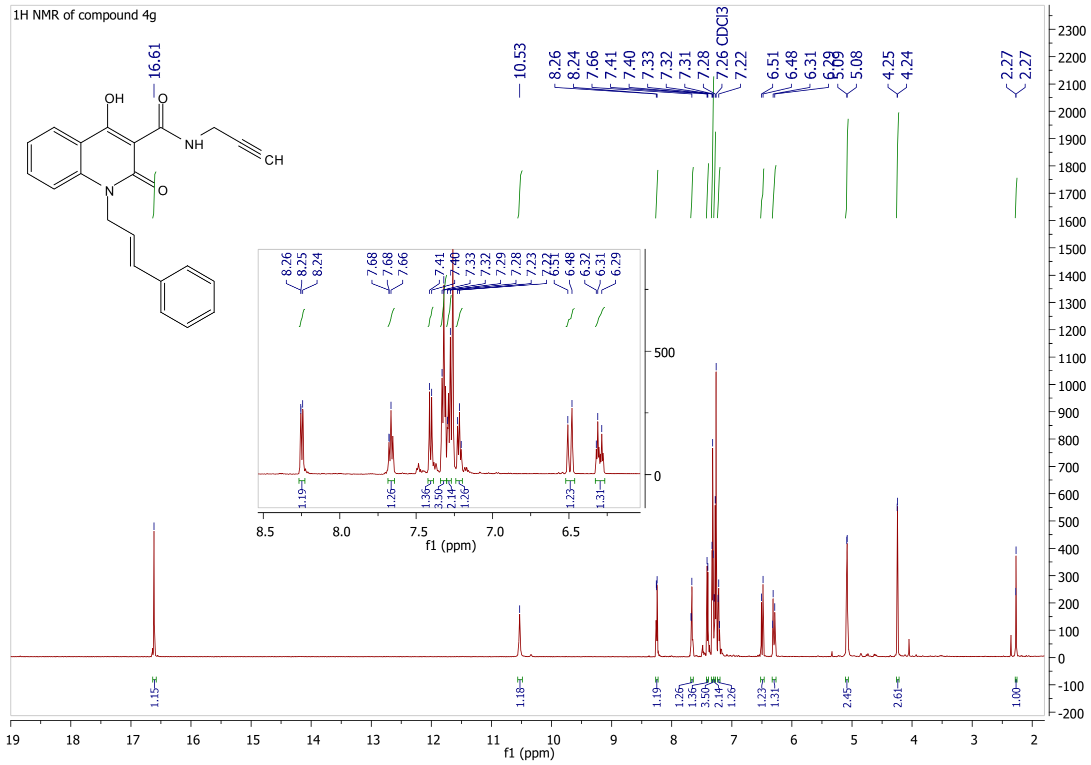

Figure S41. ^{1}H NMR of compound **4g**

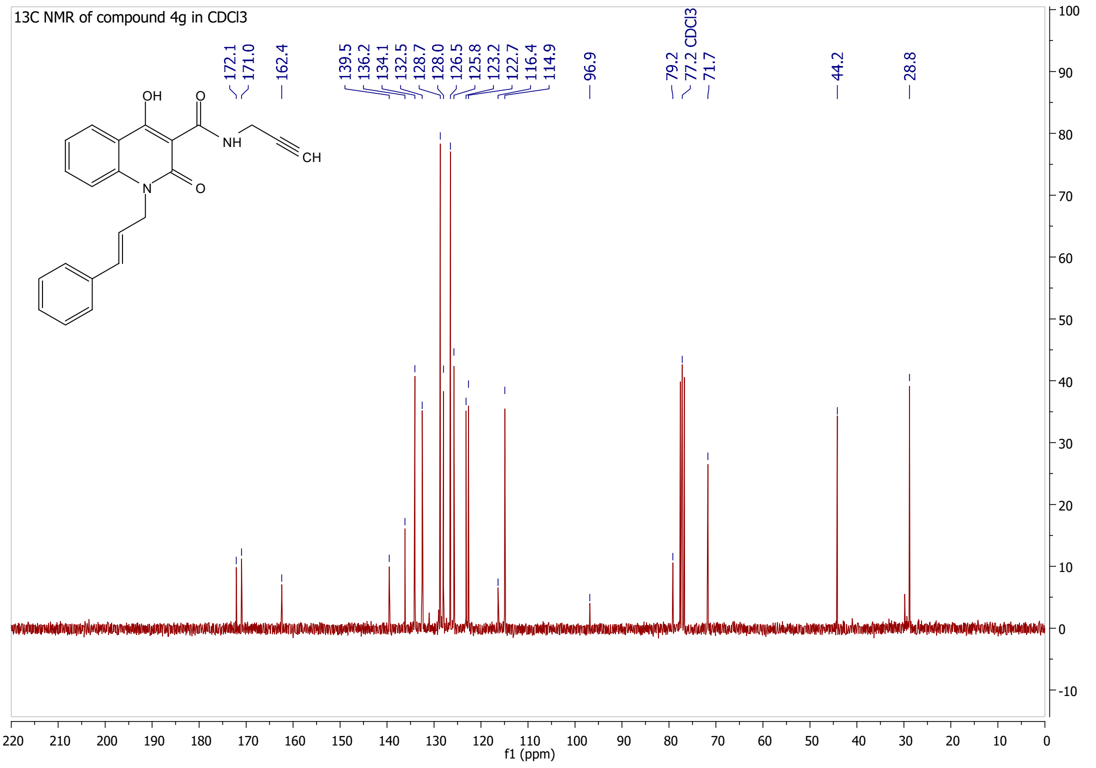

Figure S42. ^{13}C NMR of compound **4g**

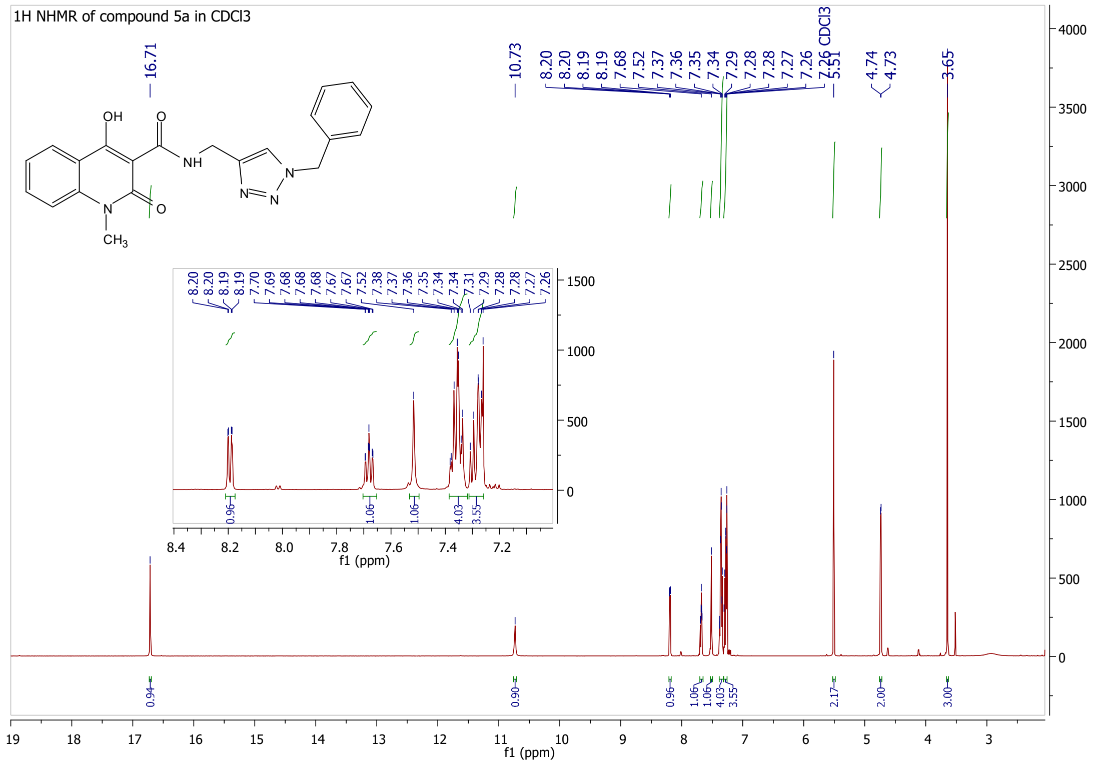

Figure S43. ^{1}H NMR of compound **5a**

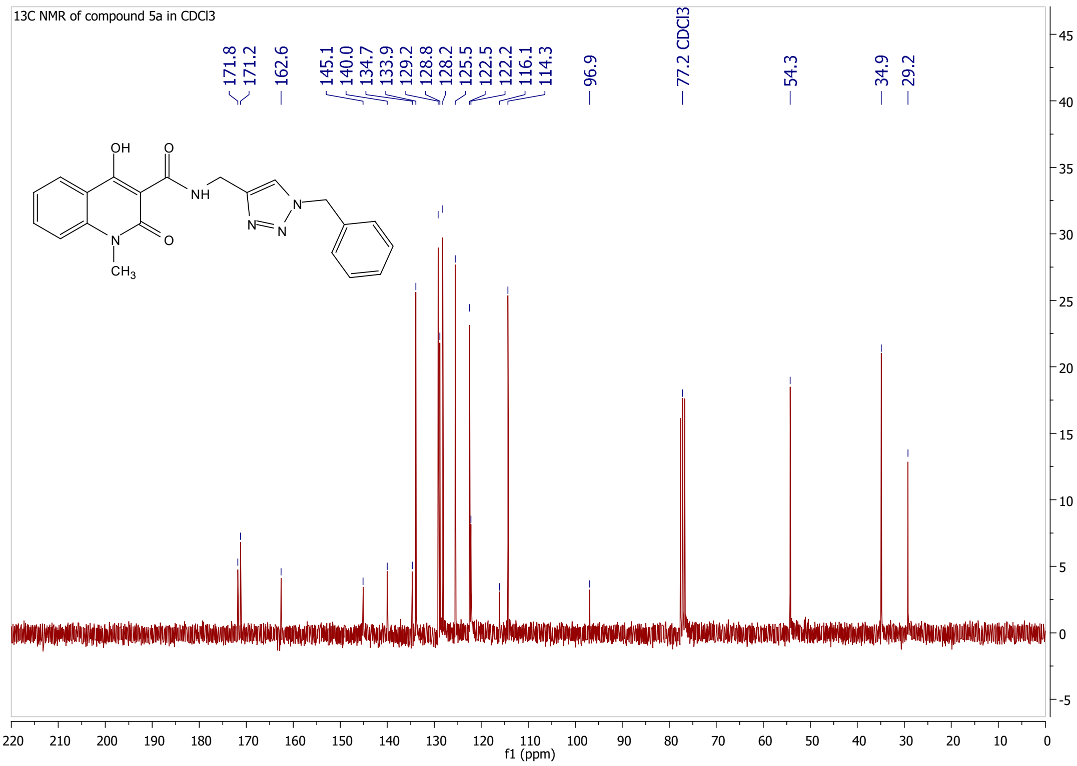

Figure S44. ^{13}C NMR of compound **5a**

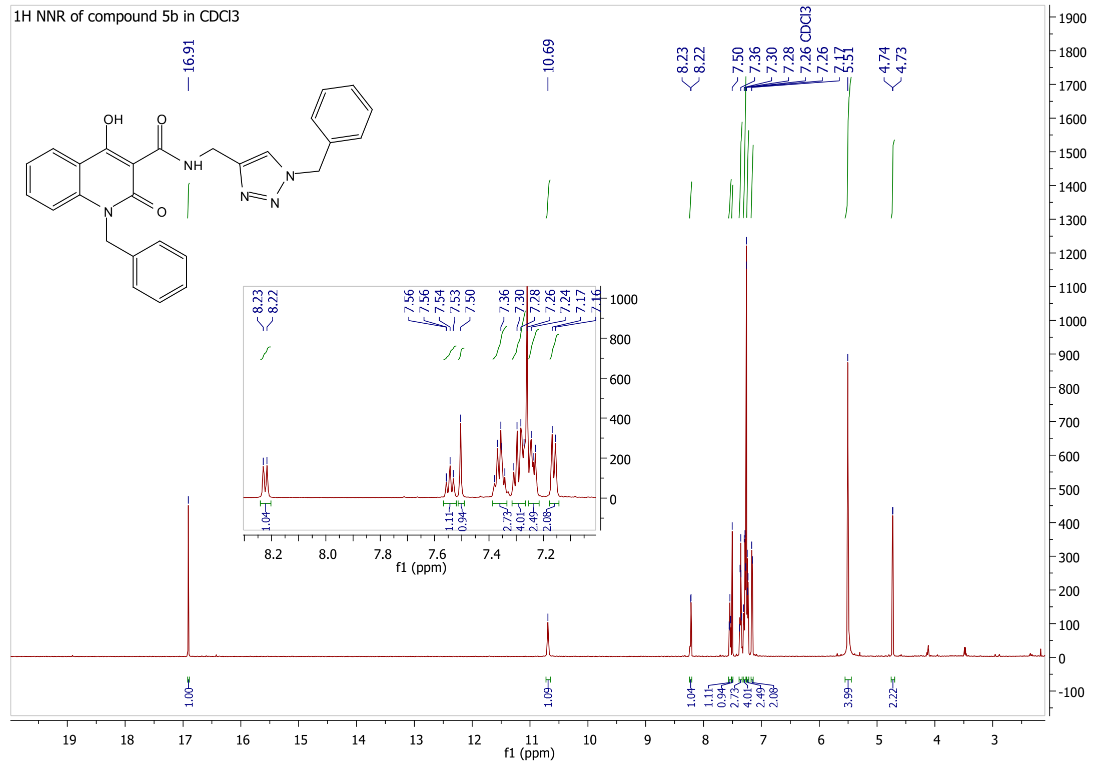

Figure S45. ^{1}H NMR of compound **5b**

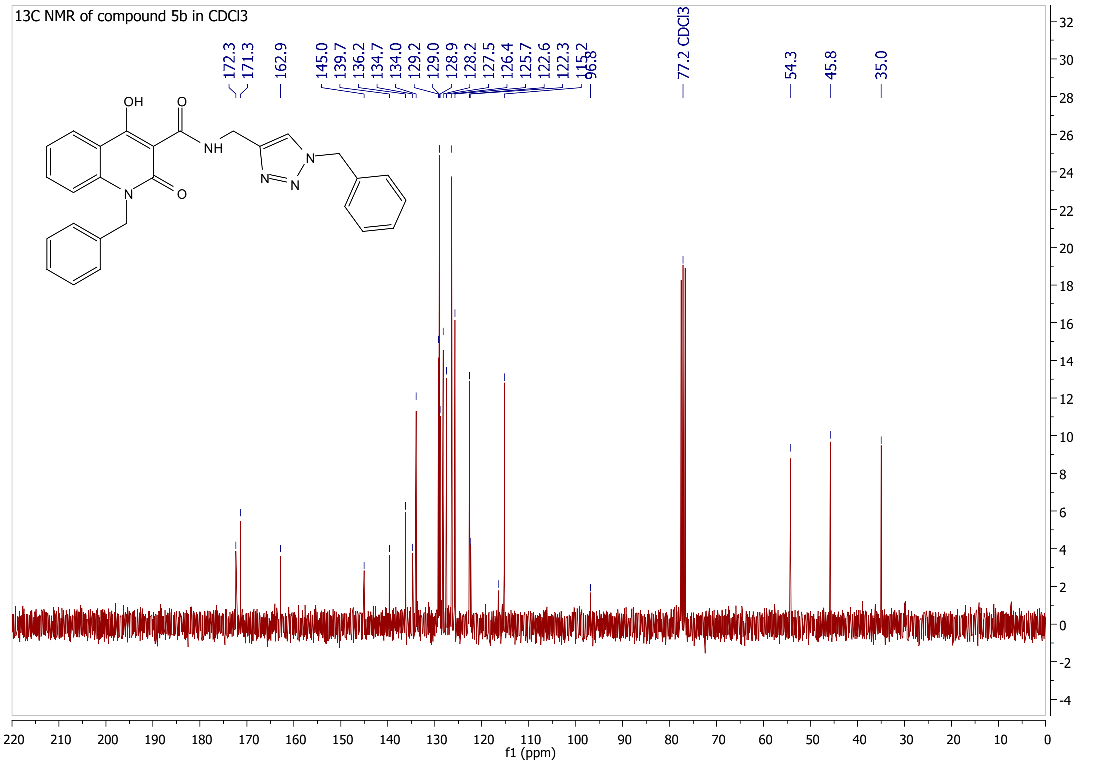

Figure S46. ^{13}C NMR of compound **5b**

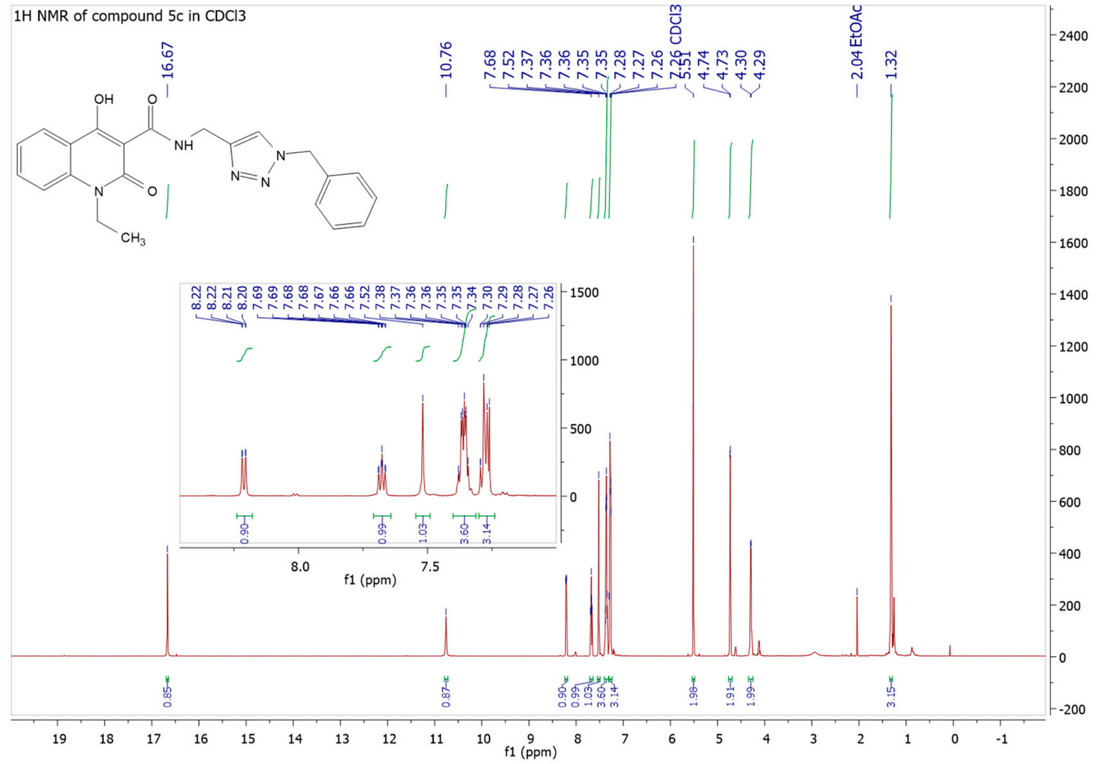

Figure S47. ^{1}H NMR of compound **5c**

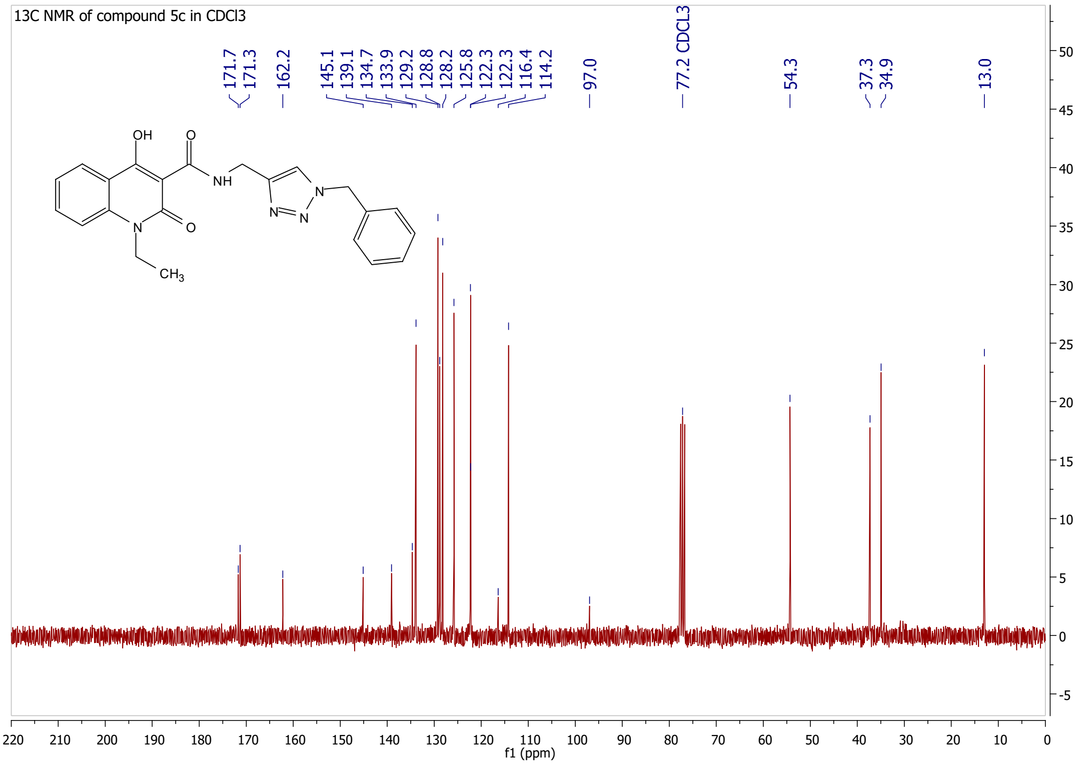

Figure S48. ^{13}C NMR of compound **5c**

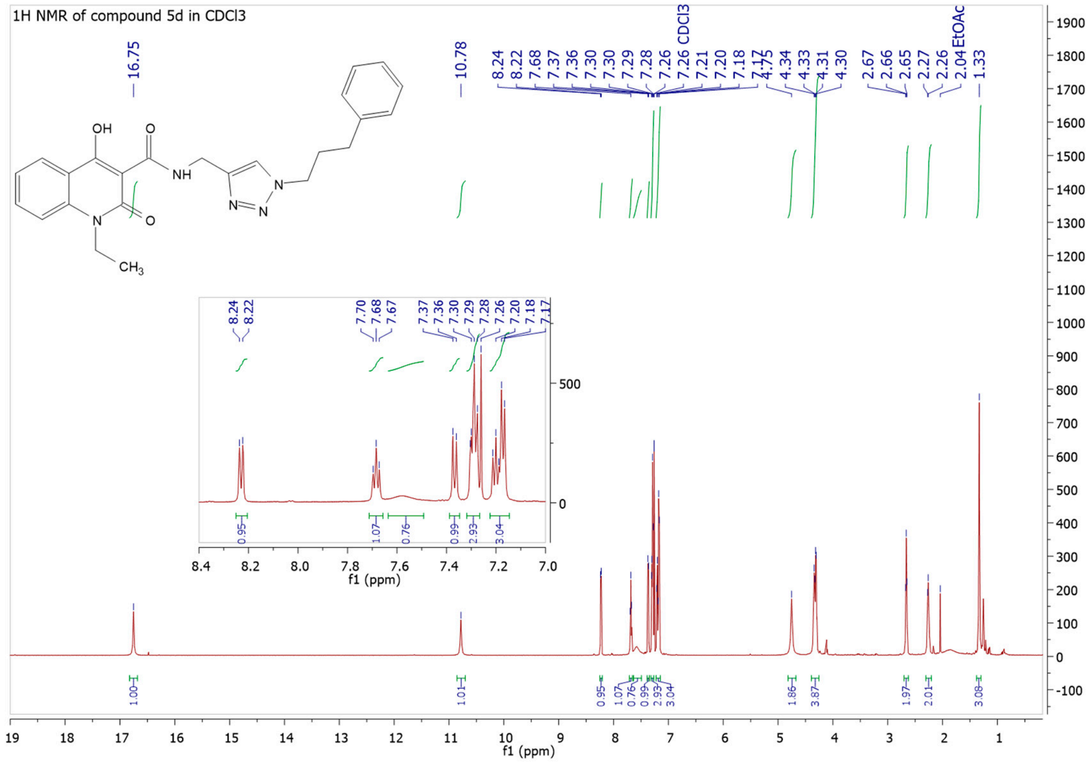

Figure S49. ^{1}H NMR of compound **5d**

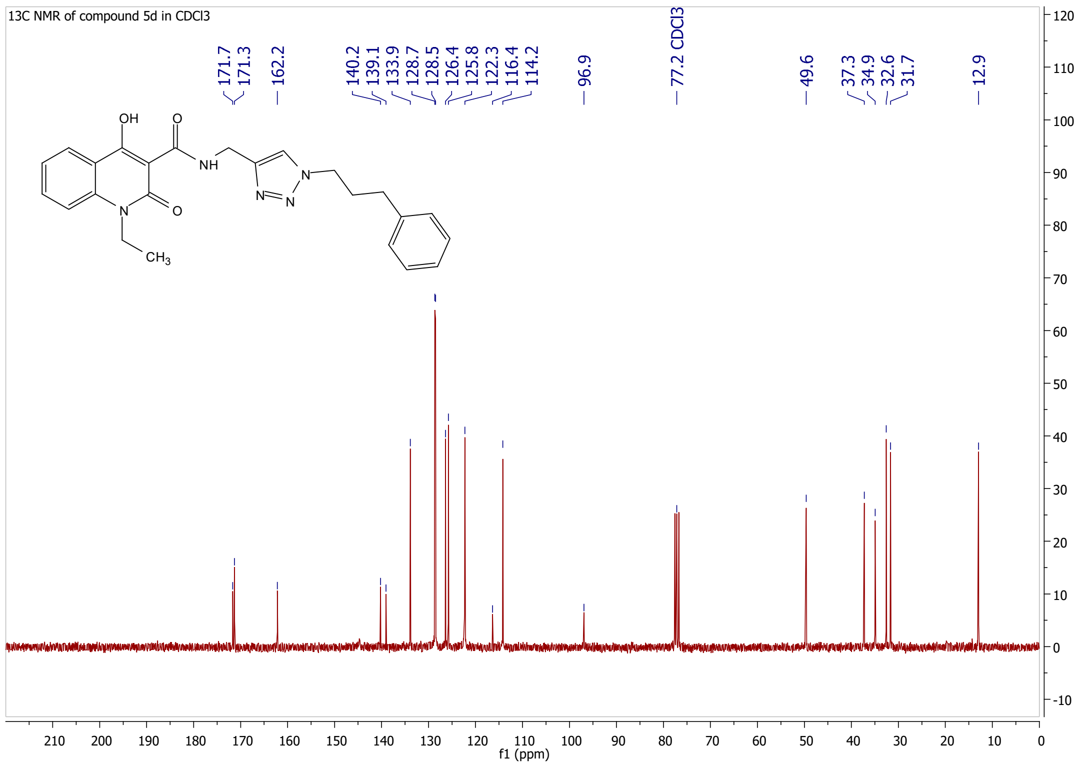

Figure S50. ^{13}C NMR of compound **5d**

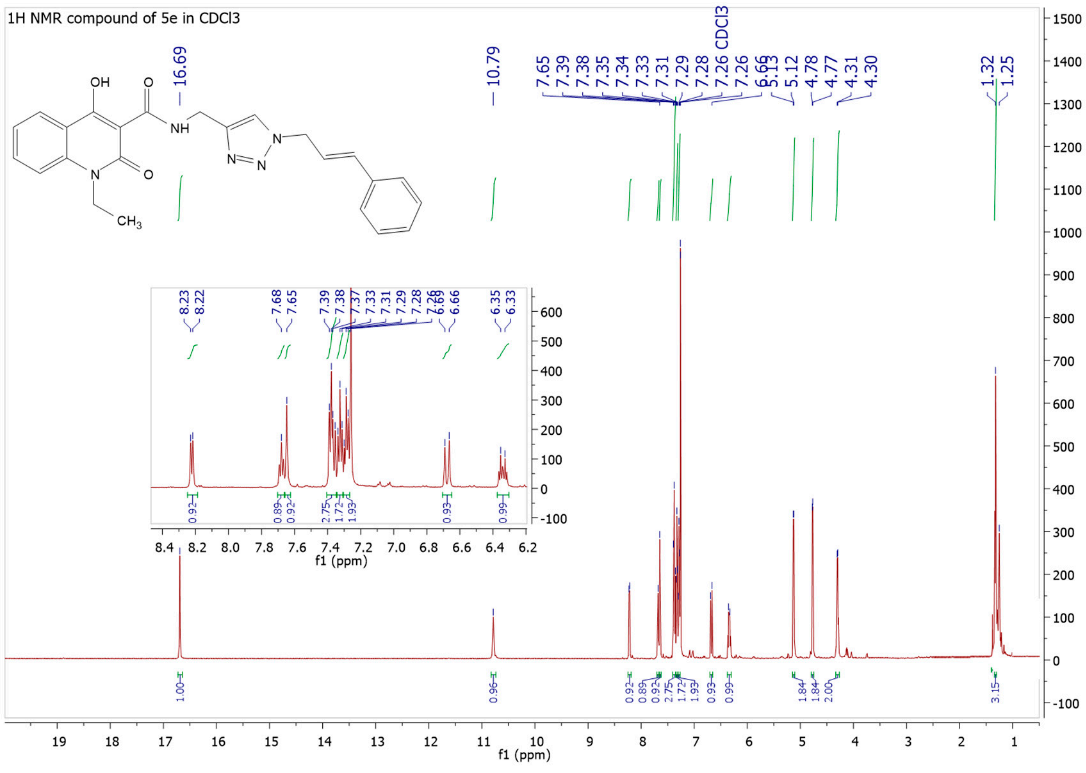

Figure S51. ^{1}H NMR of compound **5e**

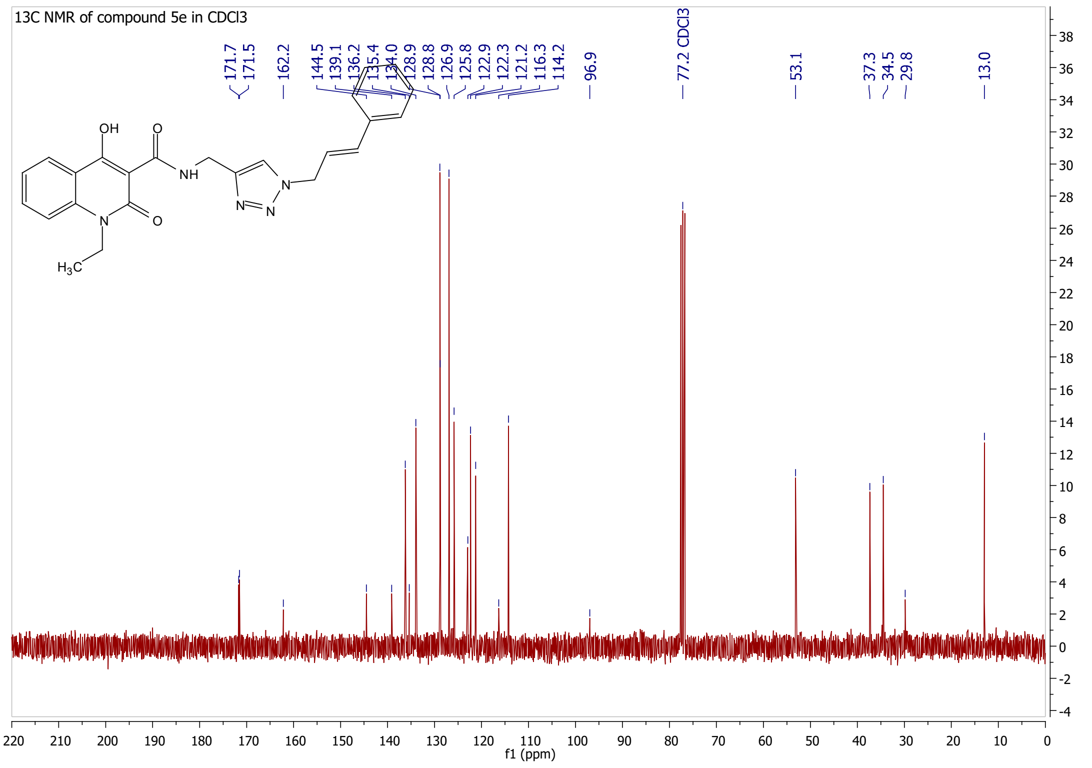

Figure S52. ¹³C NMR of compound **5e**

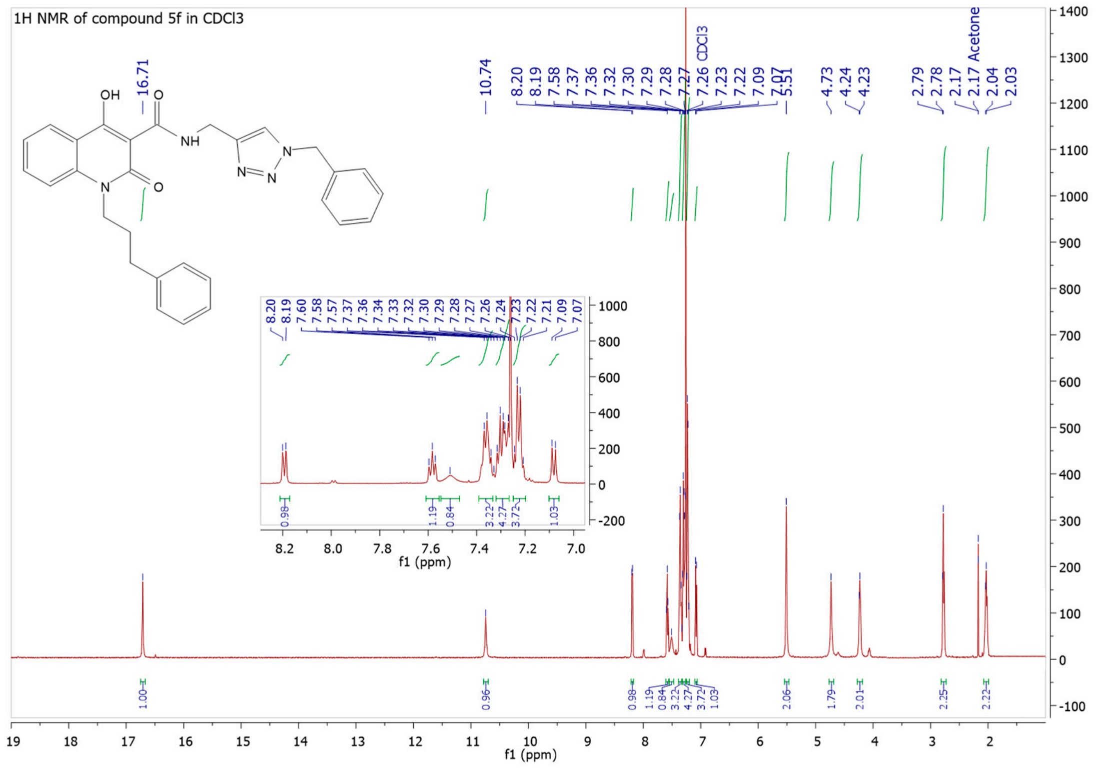

Figure S53. ¹H NMR of compound **5f**

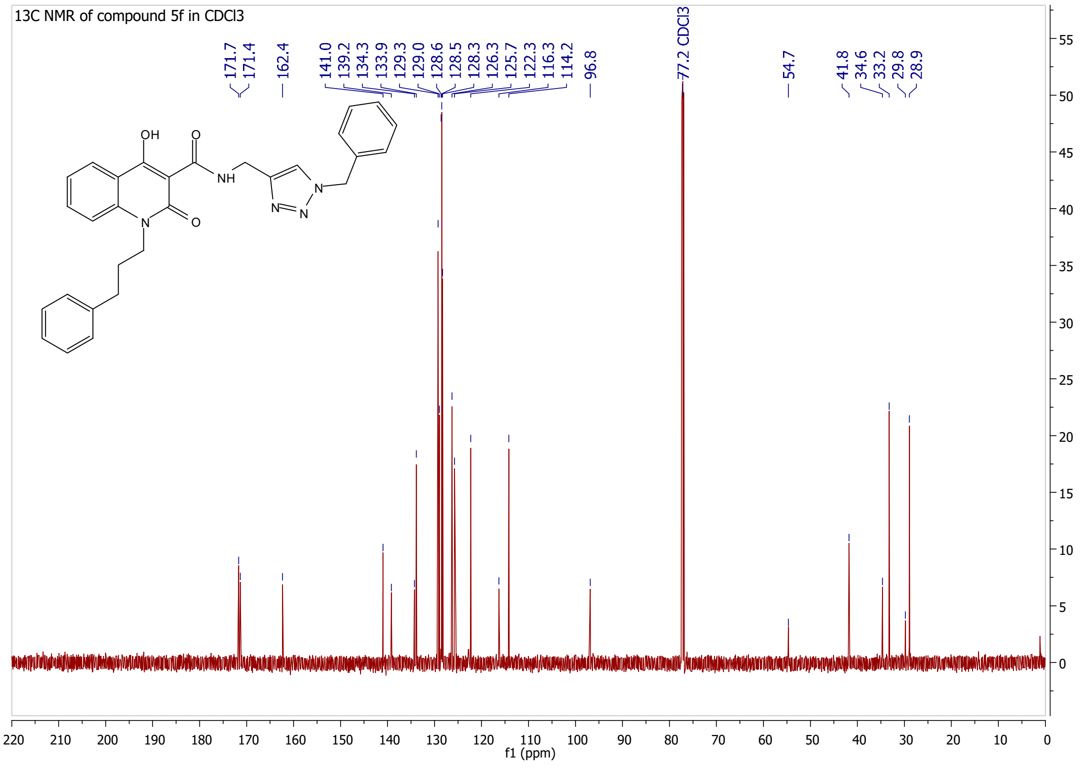

Figure S54. ^{13}C NMR of compound **5f**

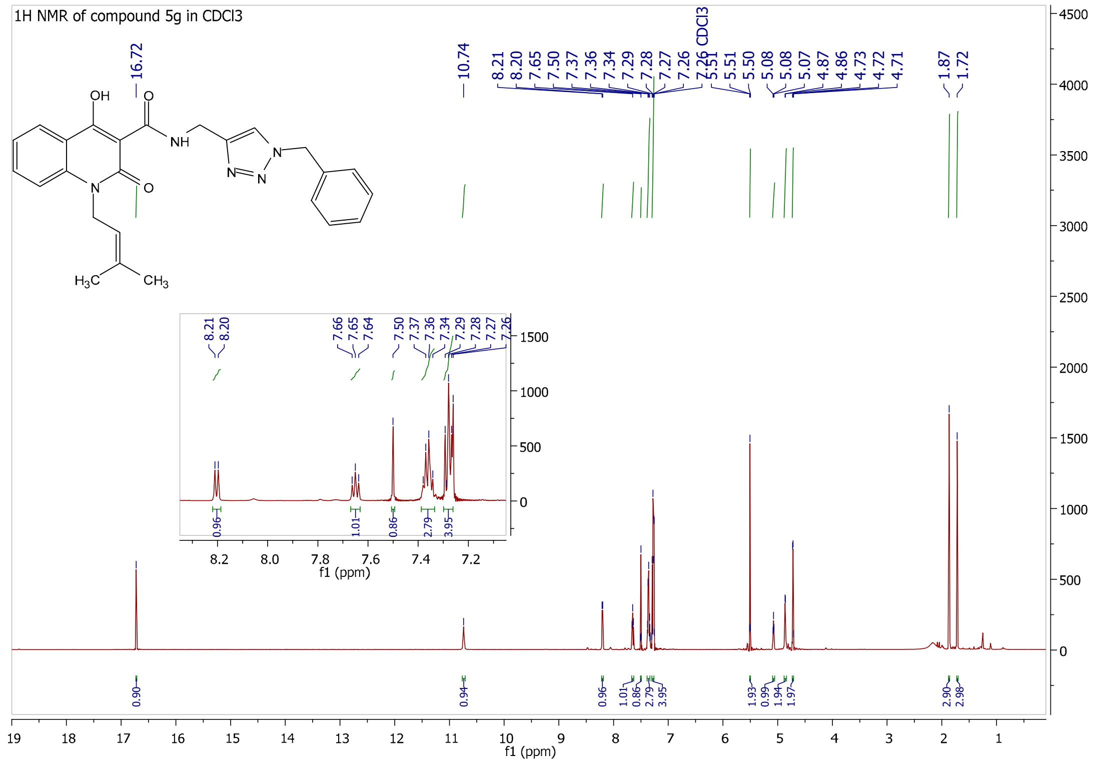

Figure S55. ^{1}H NMR of compound **5g**

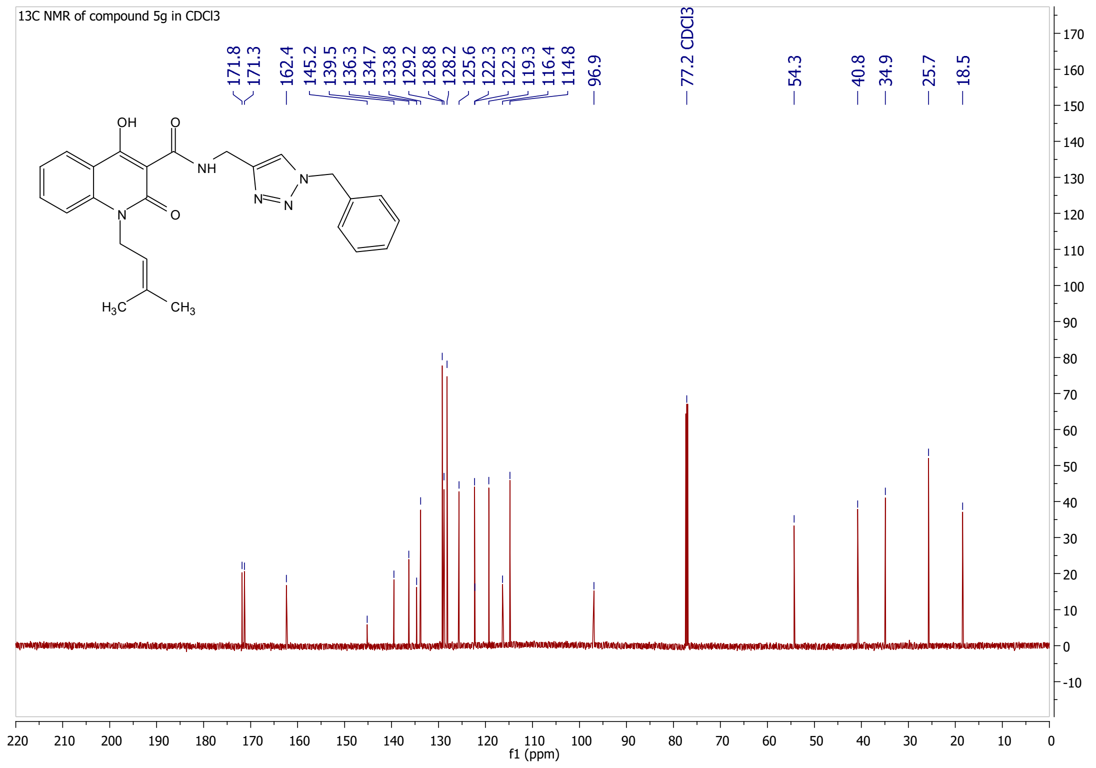

Figure S56. ^{13}C NMR of compound **5g**

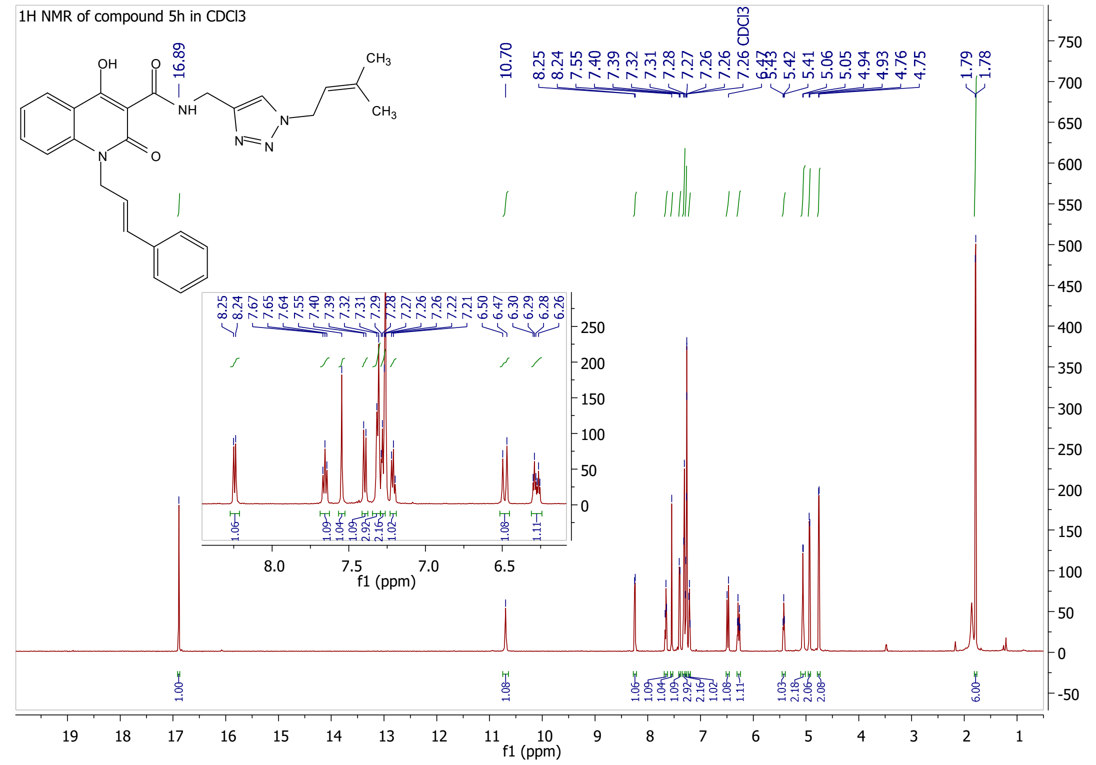

Figure S57. ^{1}H NMR of compound **5h**

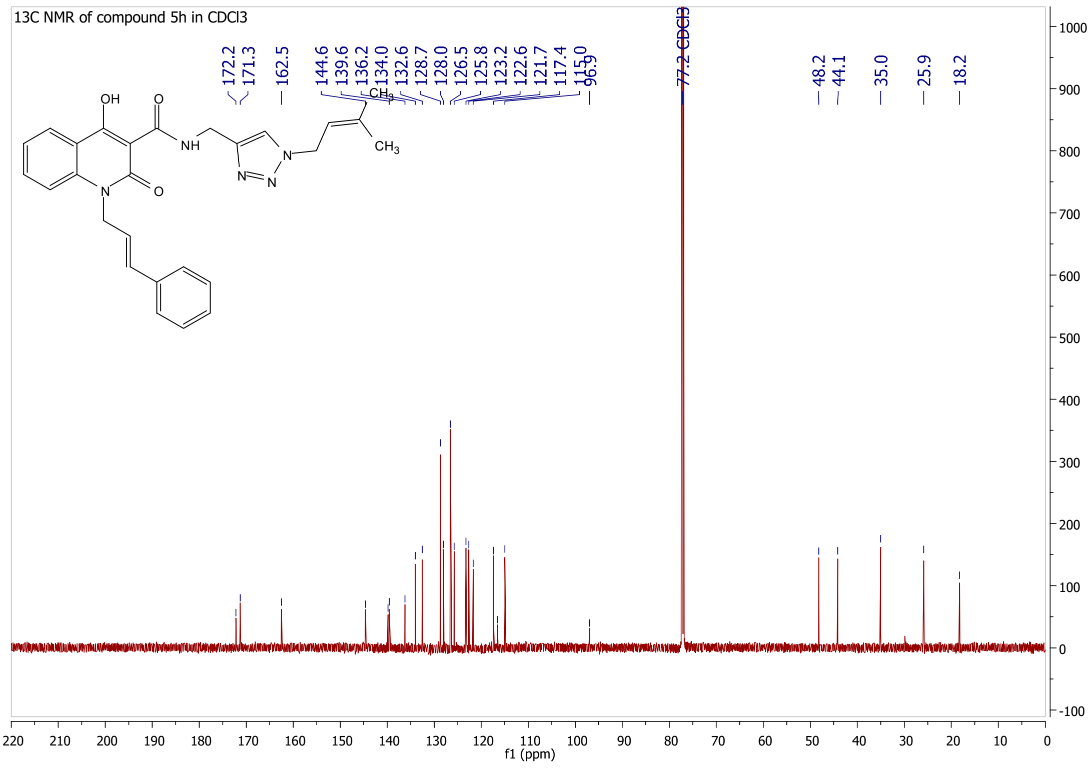

Figure S58. ^{13}C NMR of compound **5h**

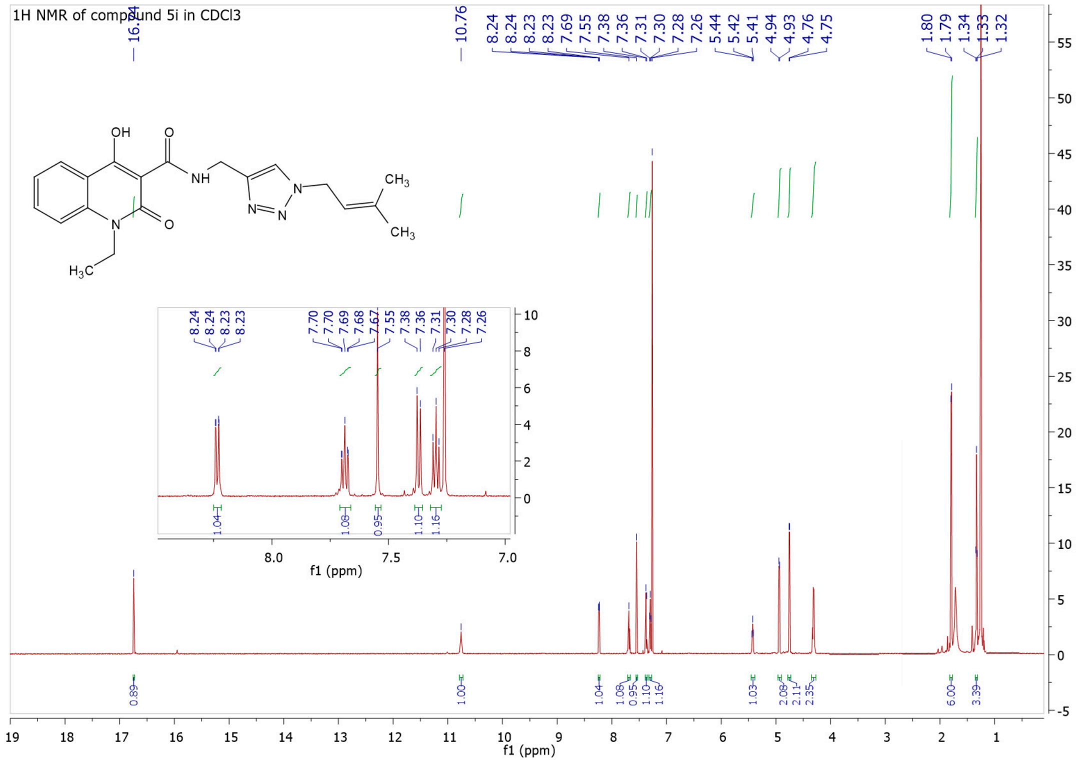

Figure S59. ^{1}H NMR of compound **5i**

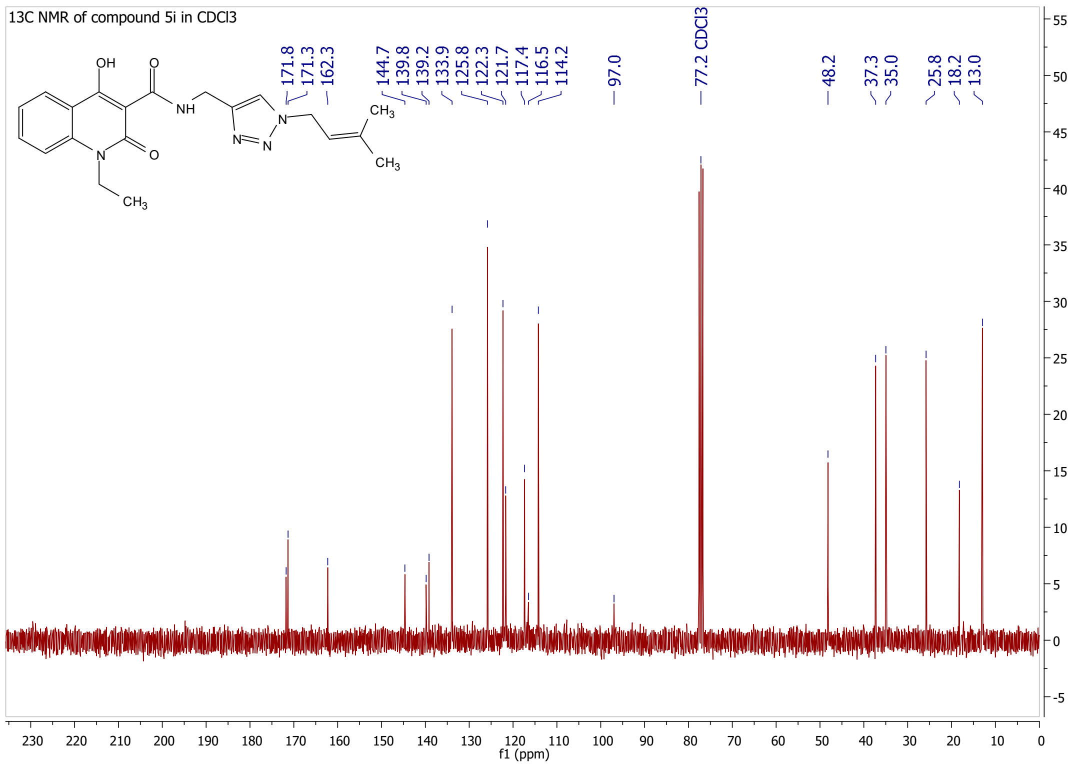

Figure S60. ^{13}C NMR of compound **5i**

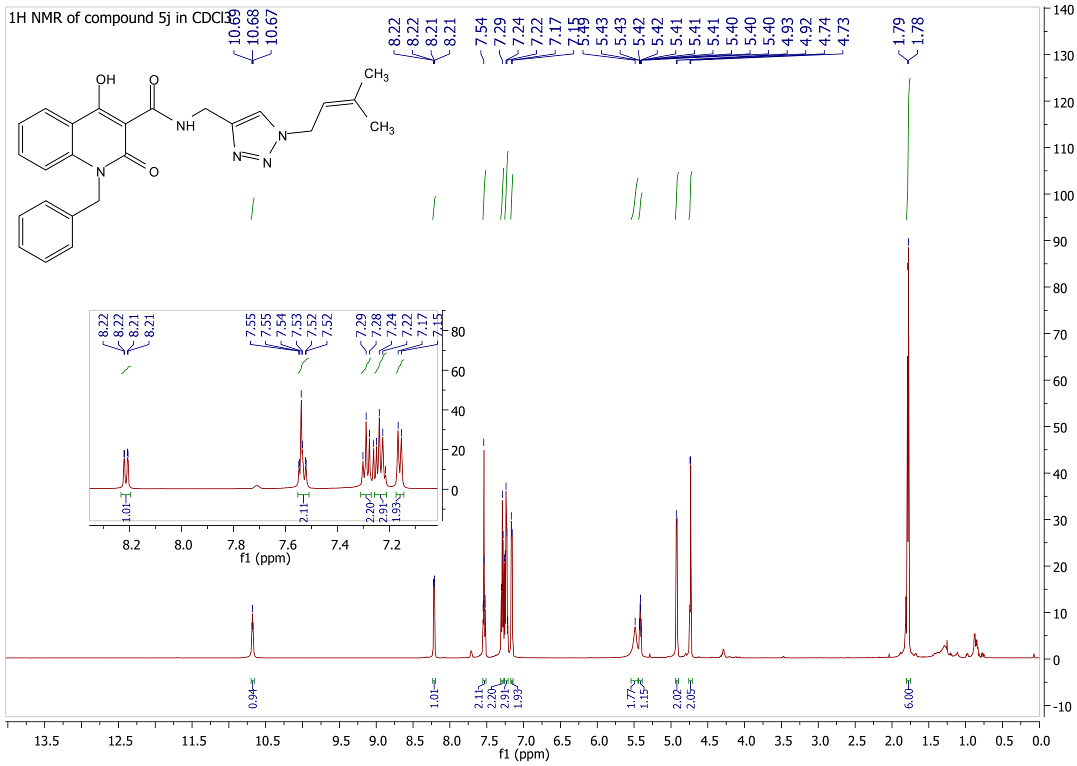

Figure S61. ^{1}H NMR of compound **5j**

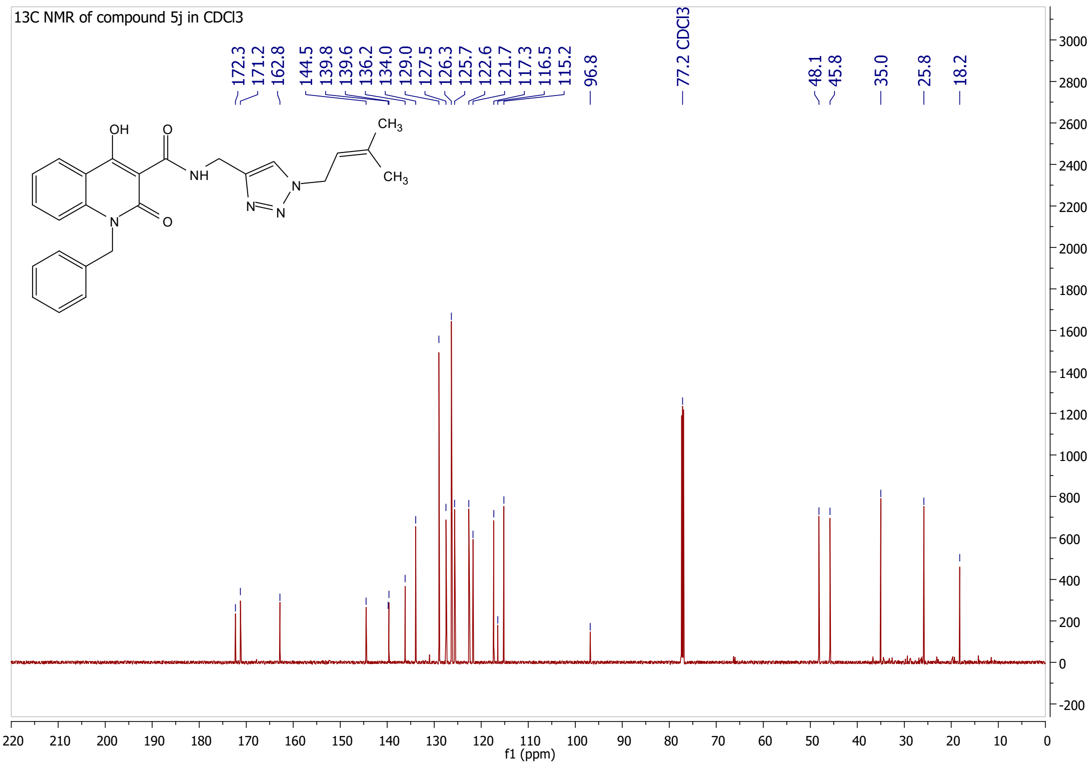

Figure S62. ^{13}C NMR of compound **5j**

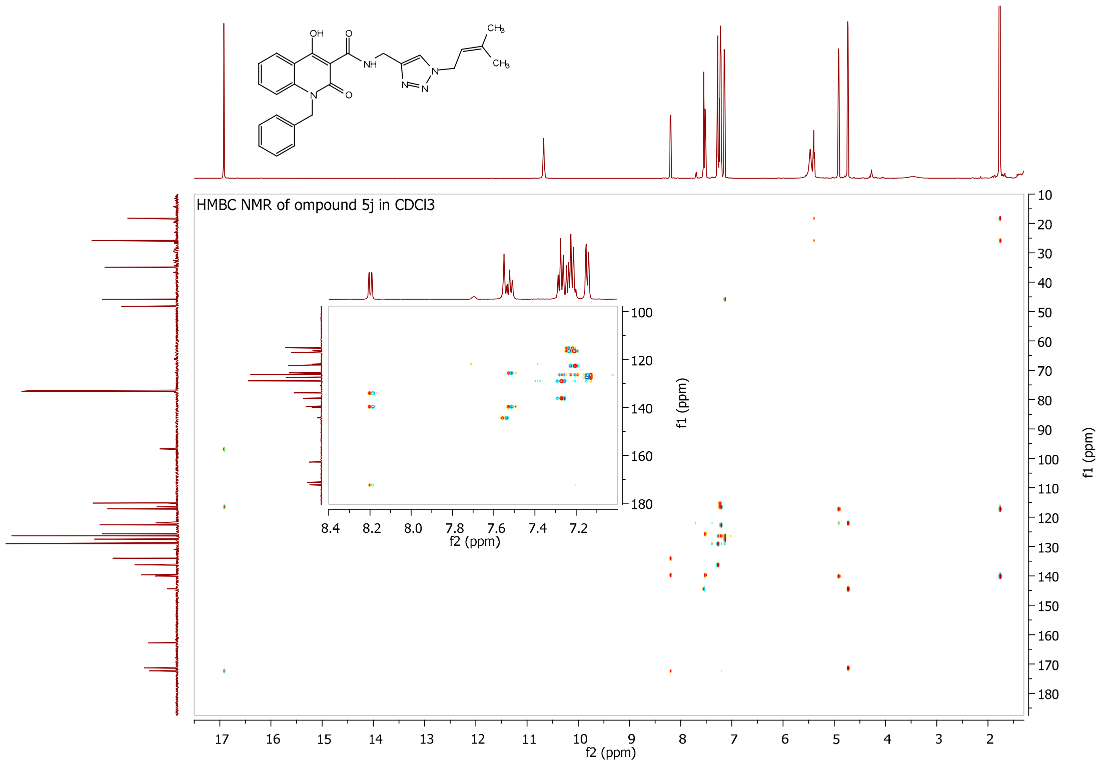

Figure S63. ^{1}H-^{13}C HMBC NMR spectrum of **5j**

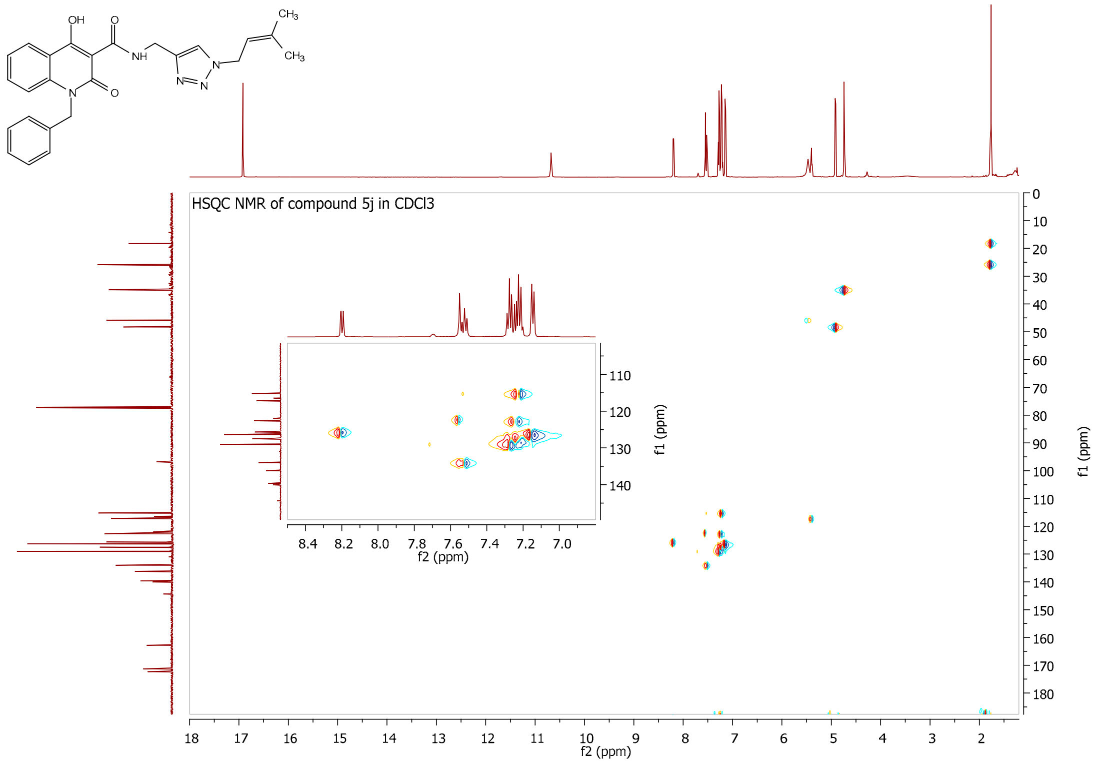

Figure S64. ${}^{1}H$-${}^{13}C$ HSQC NMR spectrum of **5j**

HR-MS data

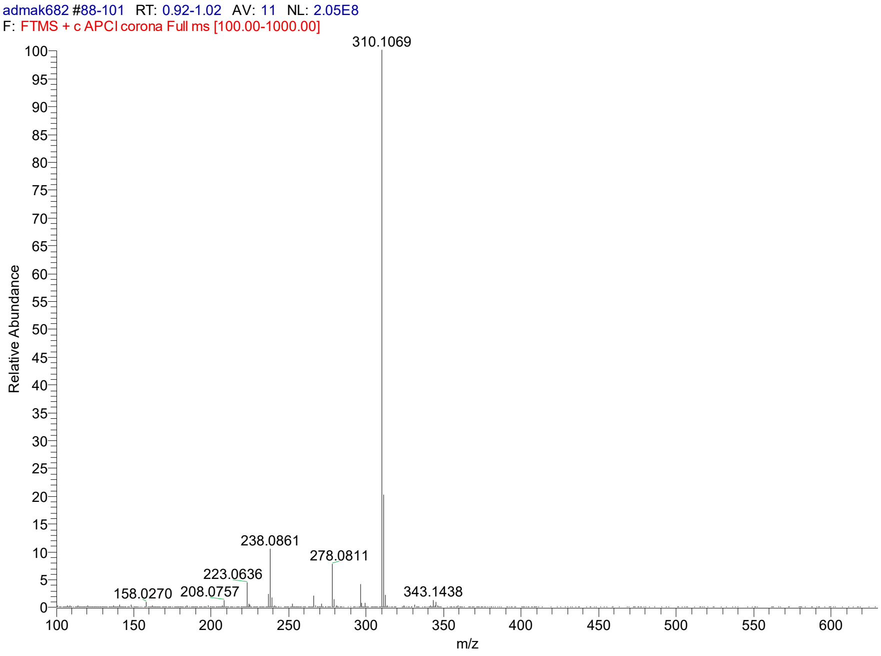

Figure S65. HR-MS spectrum of **3b**

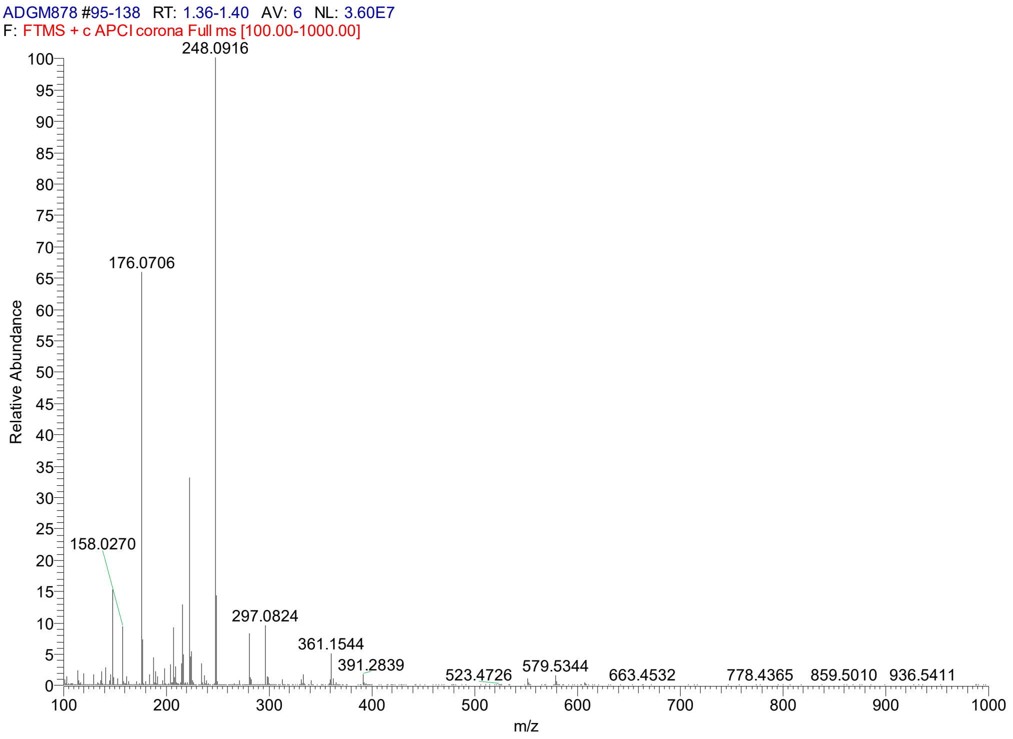

Figure S66. HR-MS spectrum of **3c**.

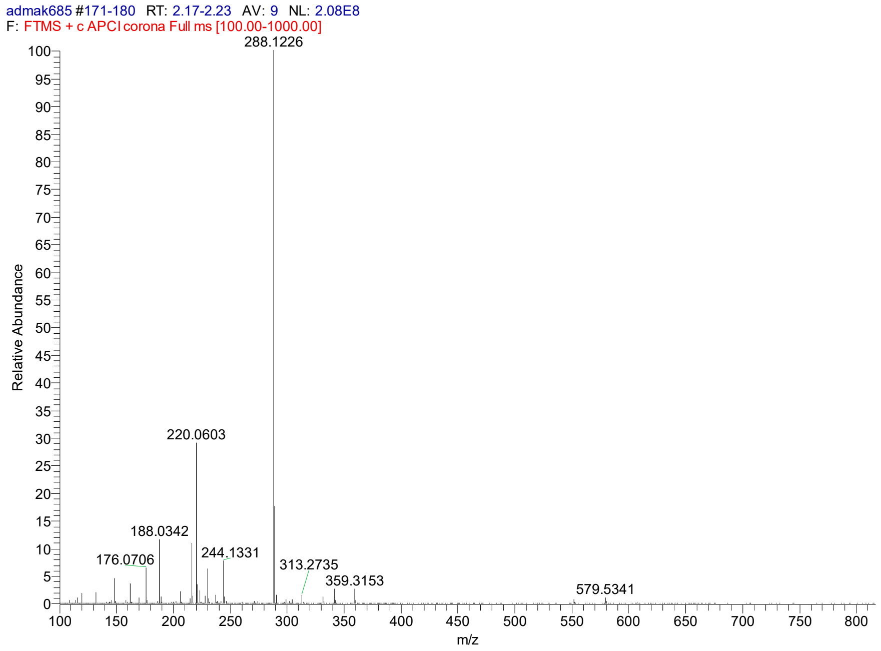

Figure S67. HR-MS spectrum of **3d**

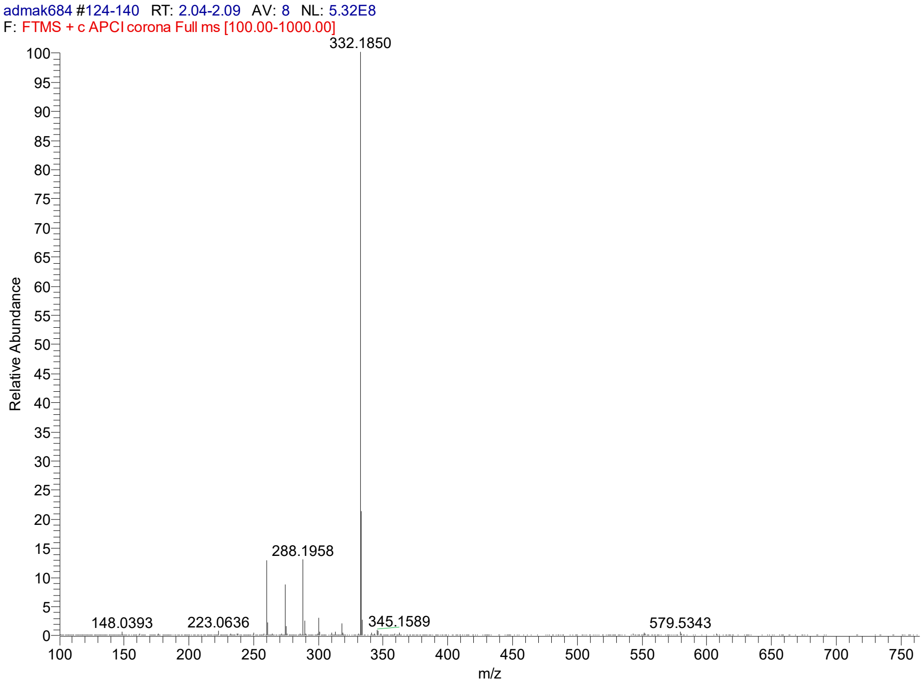

Figure S68. HR-MS spectrum of **3e**

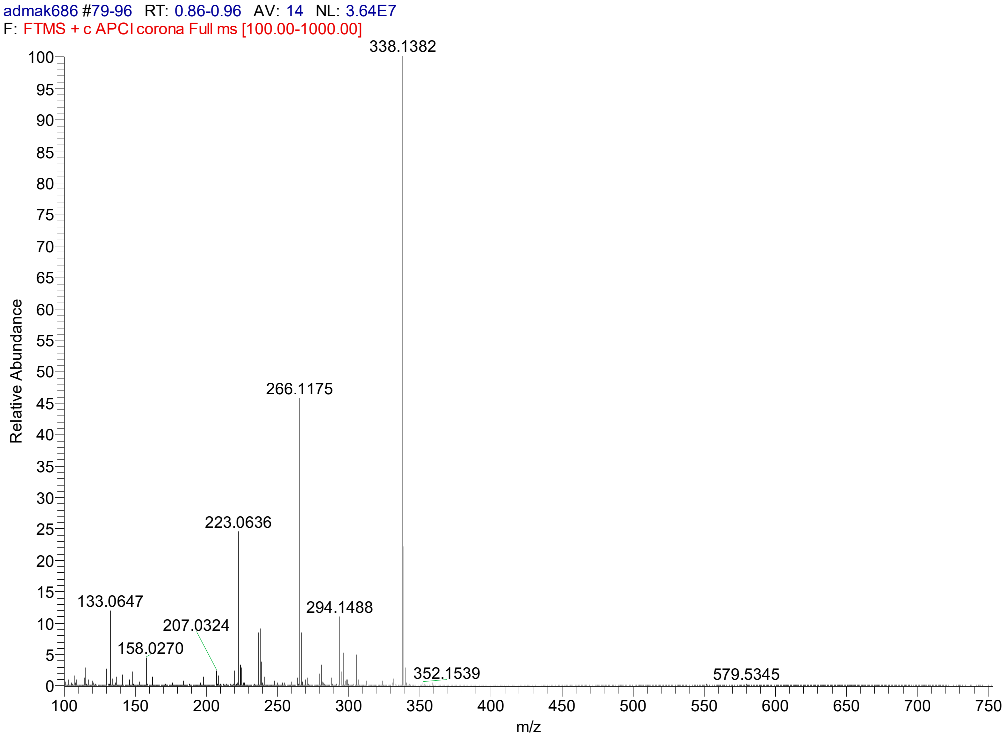

Figure S69. HR-MS spectrum of **3f**

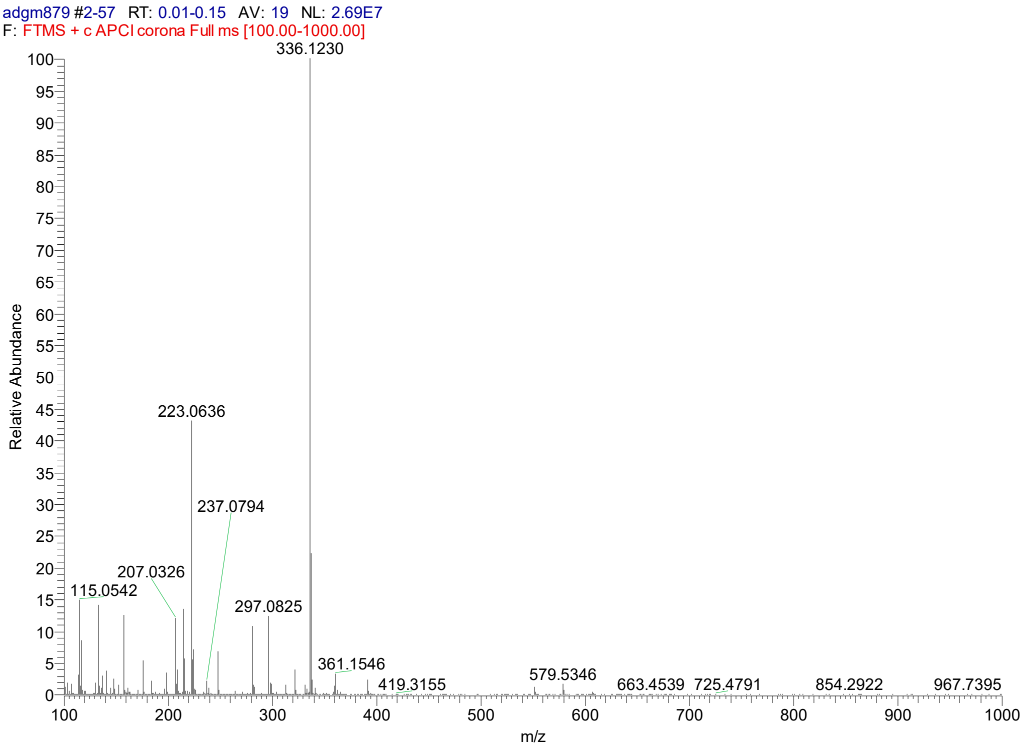

Figure S70. HR-MS spectrum of **3g**

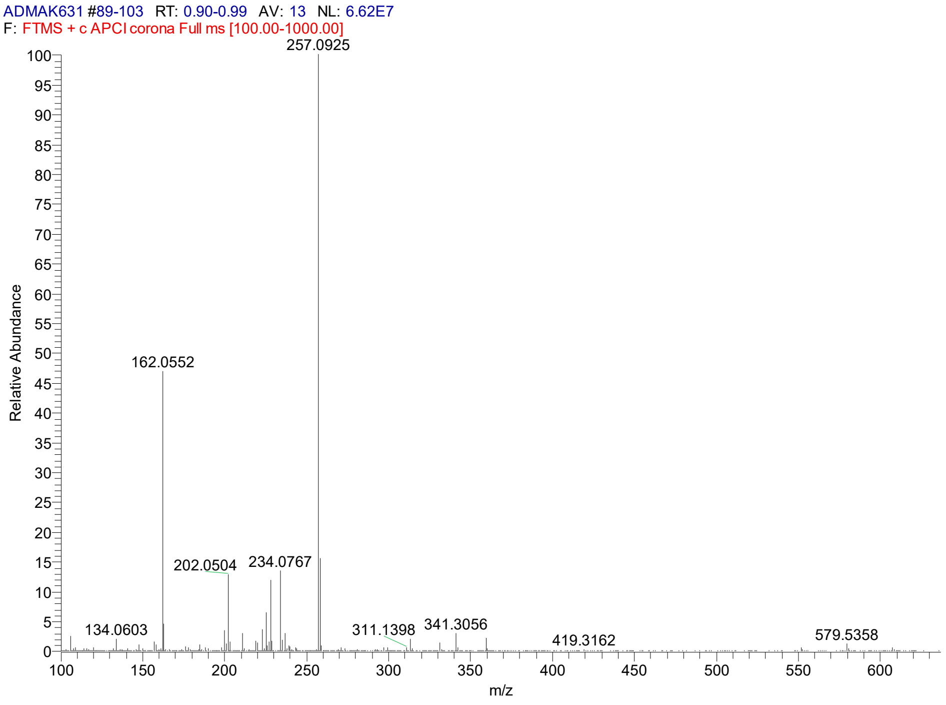

Figure S71. HR-MS spectrum of **4a**.

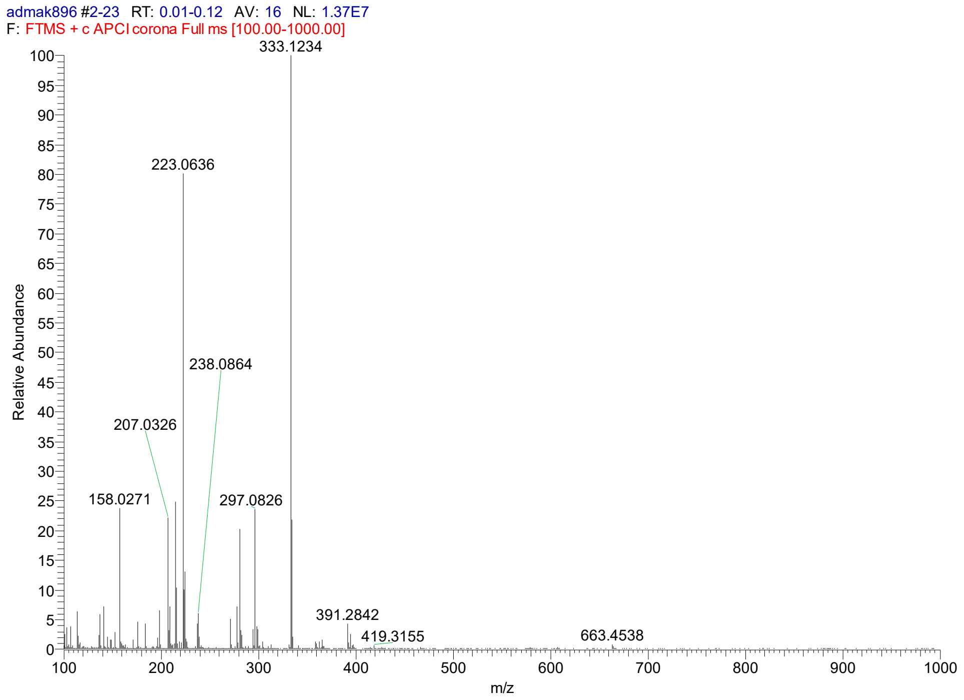

Figure S72. HR-MS spectrum of **4b**.

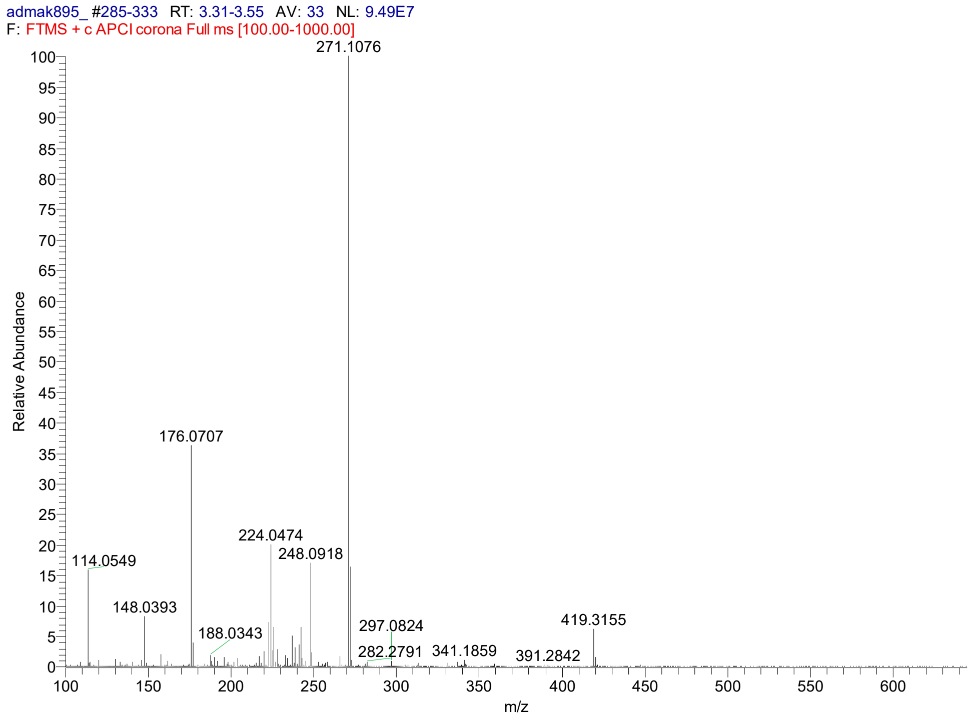

Figure S73. HR-MS spectrum of **4c**

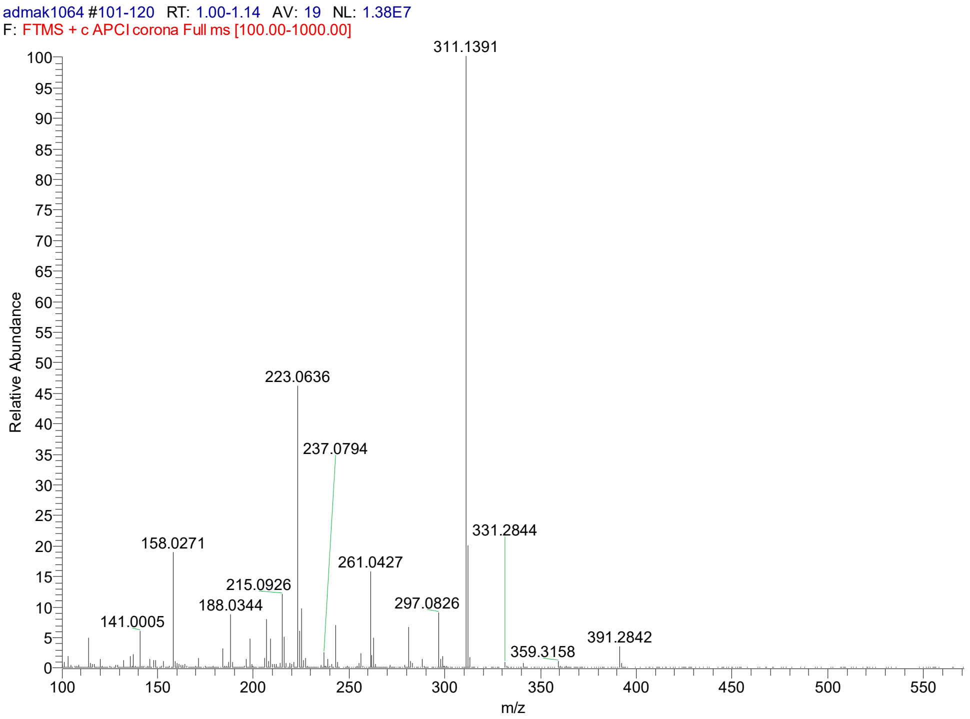

Figure S74. HR-MS spectrum of **4d**

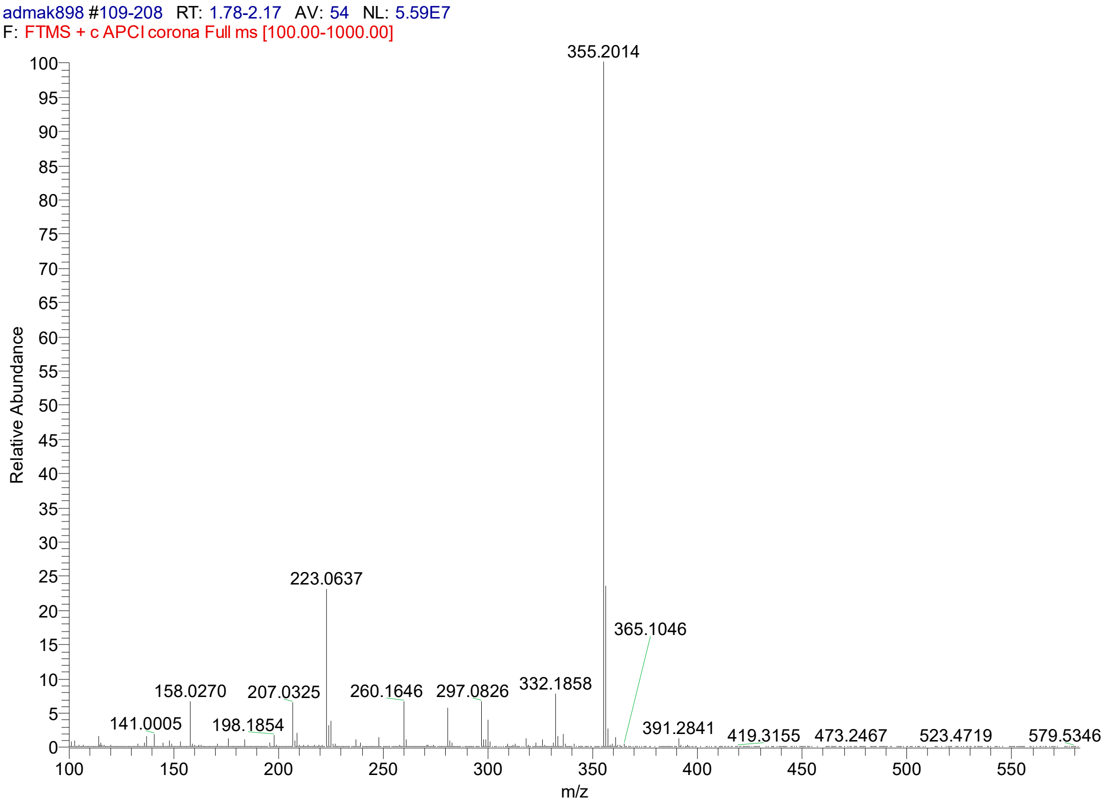

Figure S75. HR-MS spectrum of **4e**

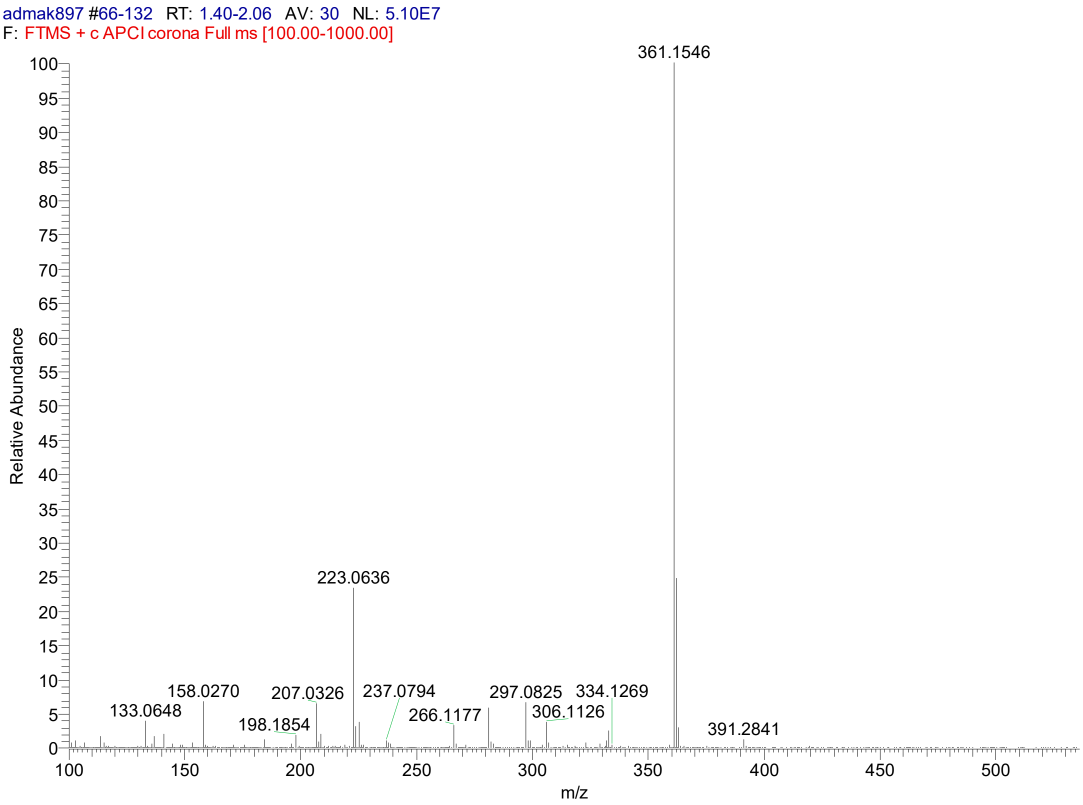

Figure S76. HR-MS spectrum of **4f**

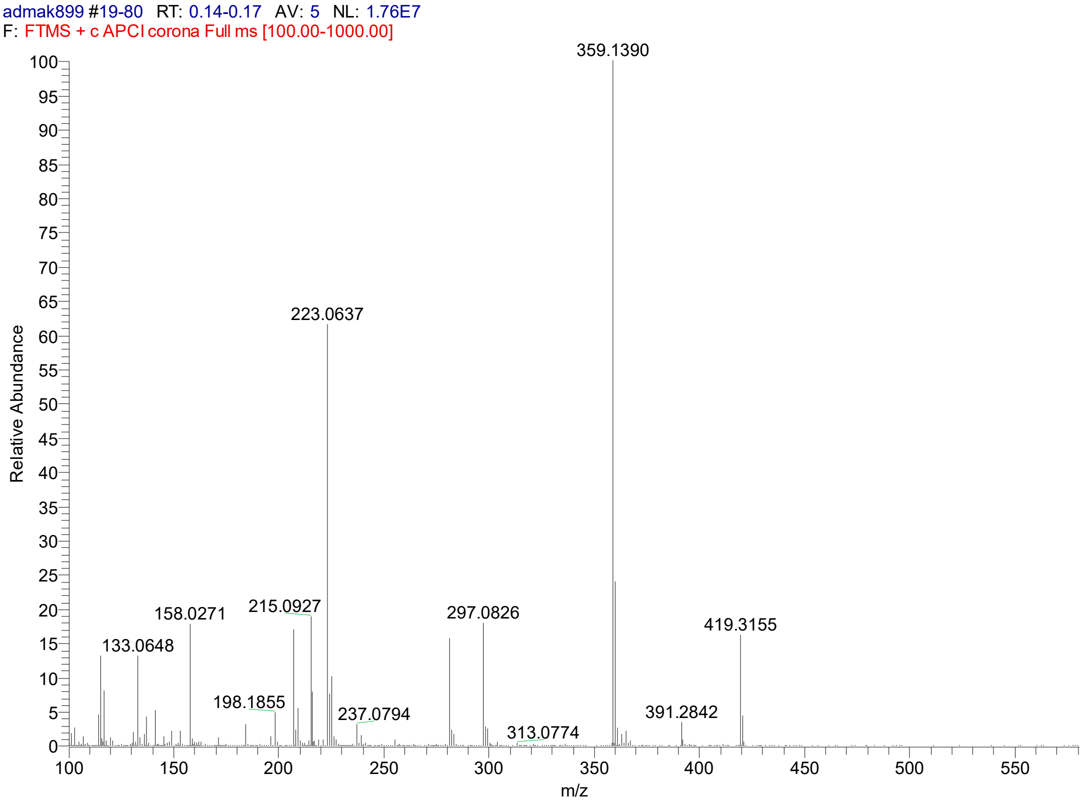

Figure S77. HR-MS spectrum of **4g**

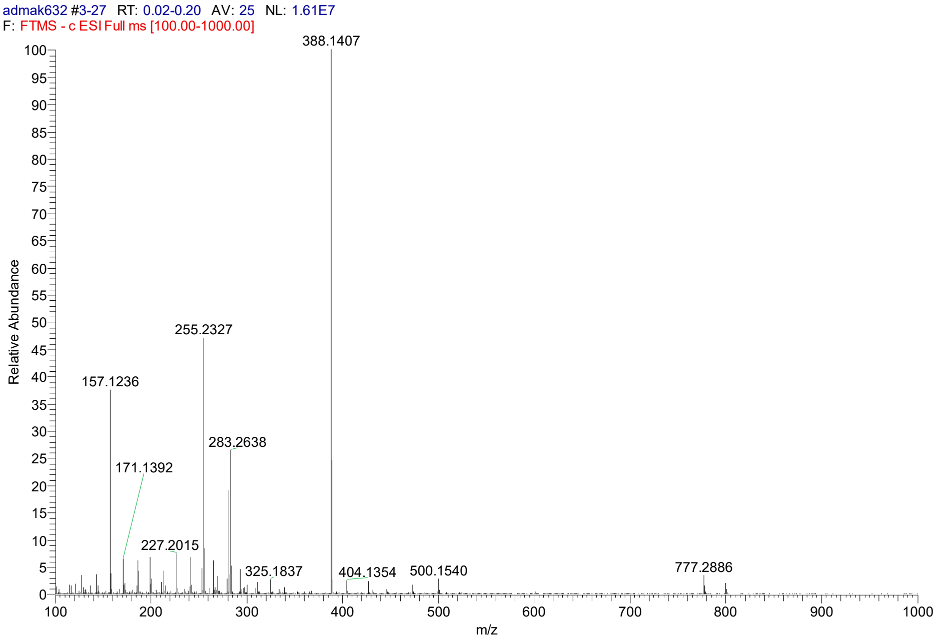

Figure S78. HR-MS spectrum of **5a**

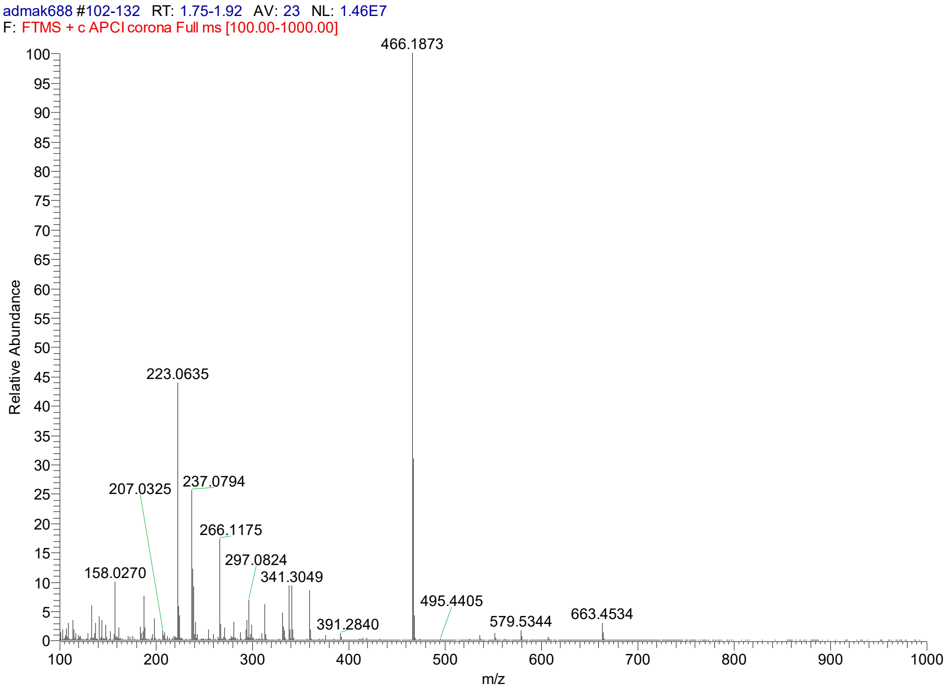

Figure S79. HR-MS spectrum of **5b**

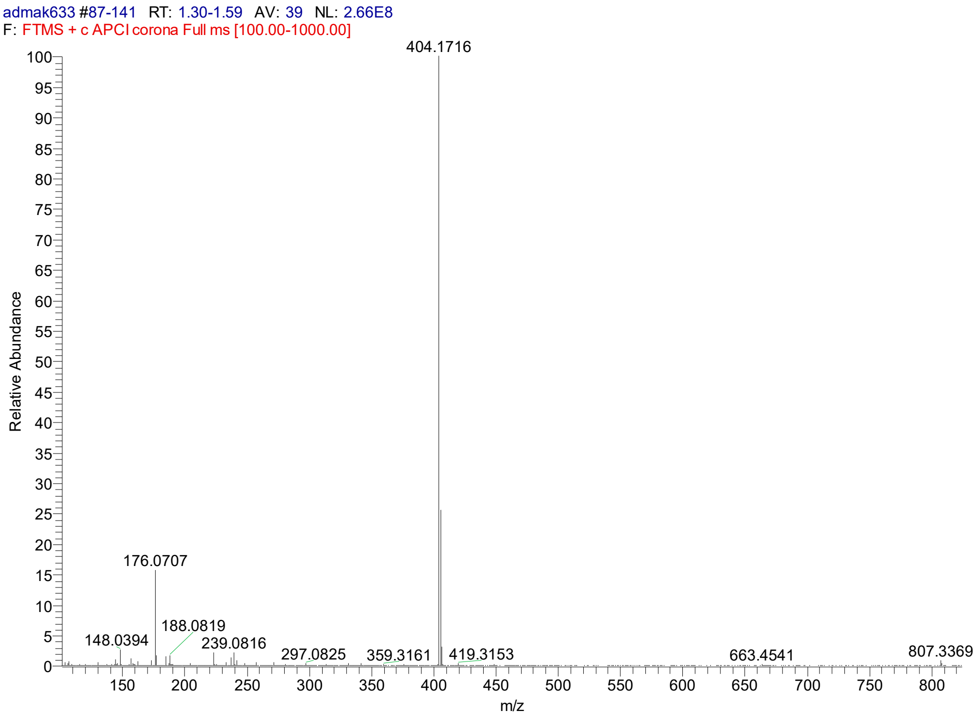

Figure S80. HR-MS spectrum of **5c**

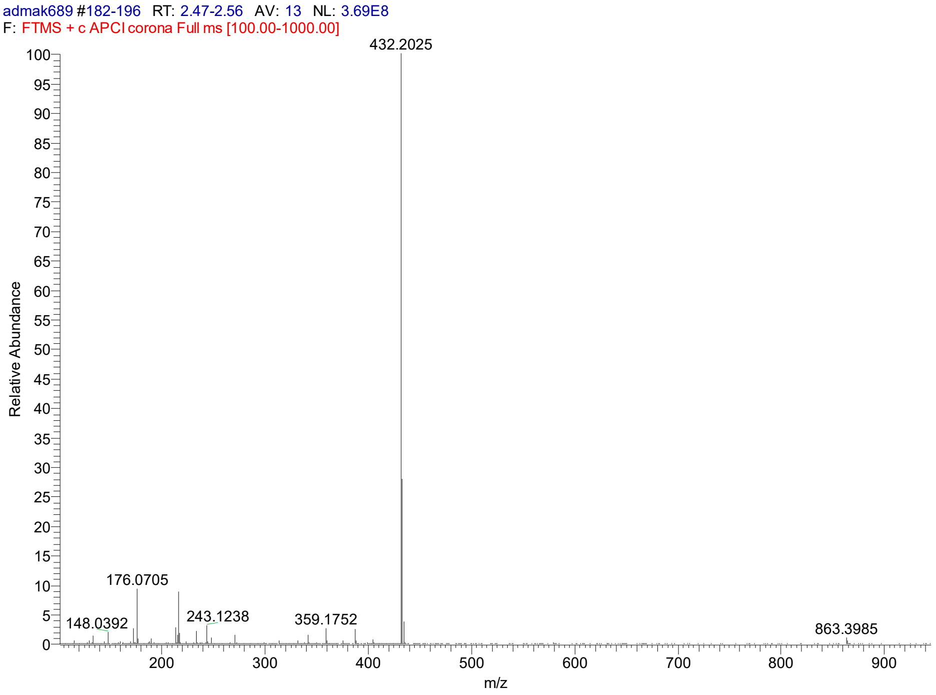

Figure S81. HR-MS spectrum of **5d**

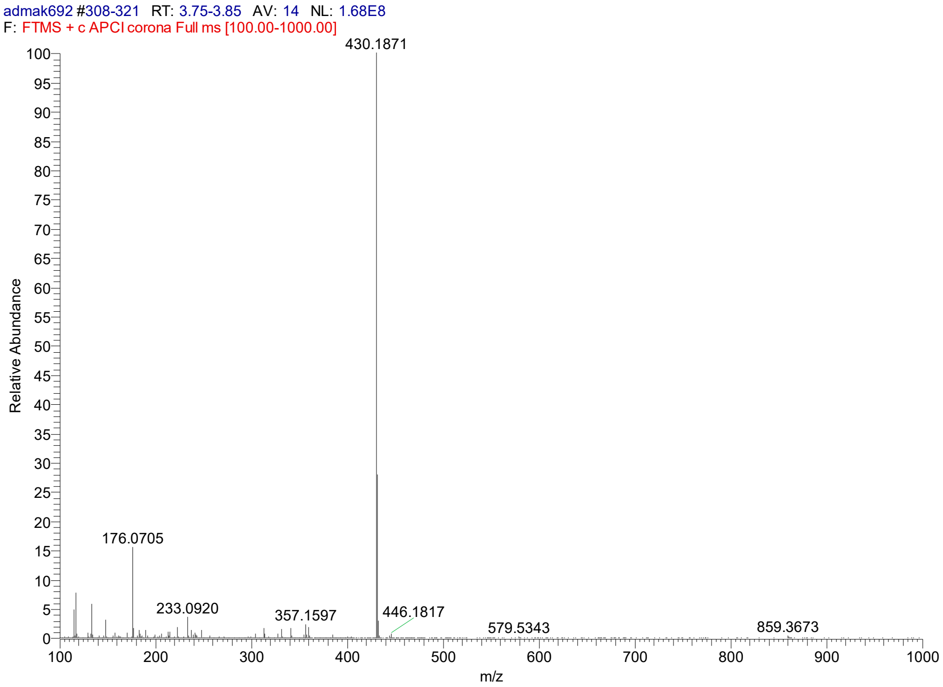

Figure S82. HR-MS spectrum of **5e**

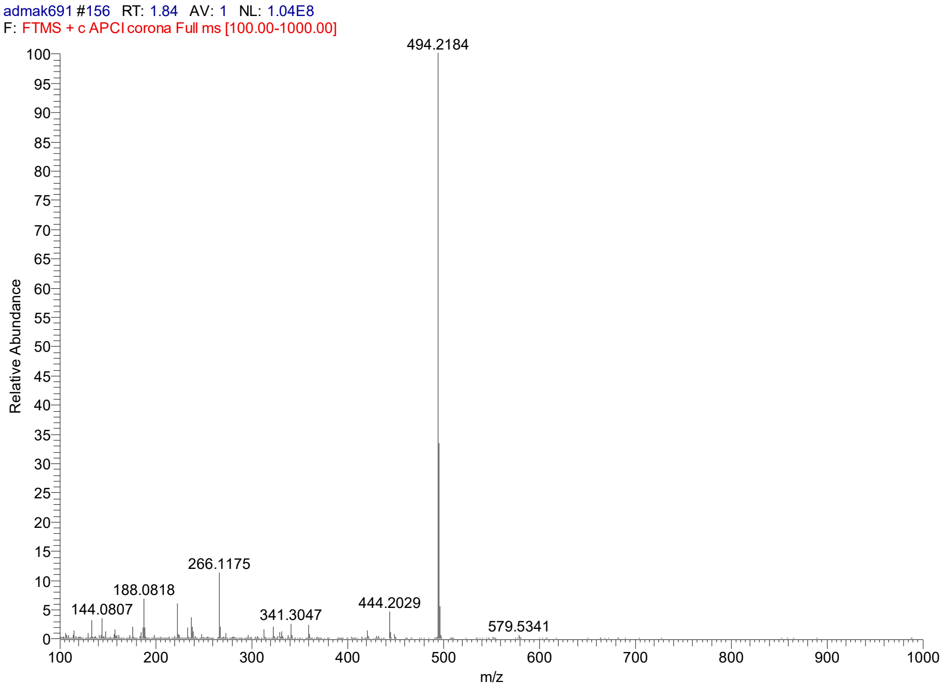

Figure S83. HR-MS spectrum of **5f**

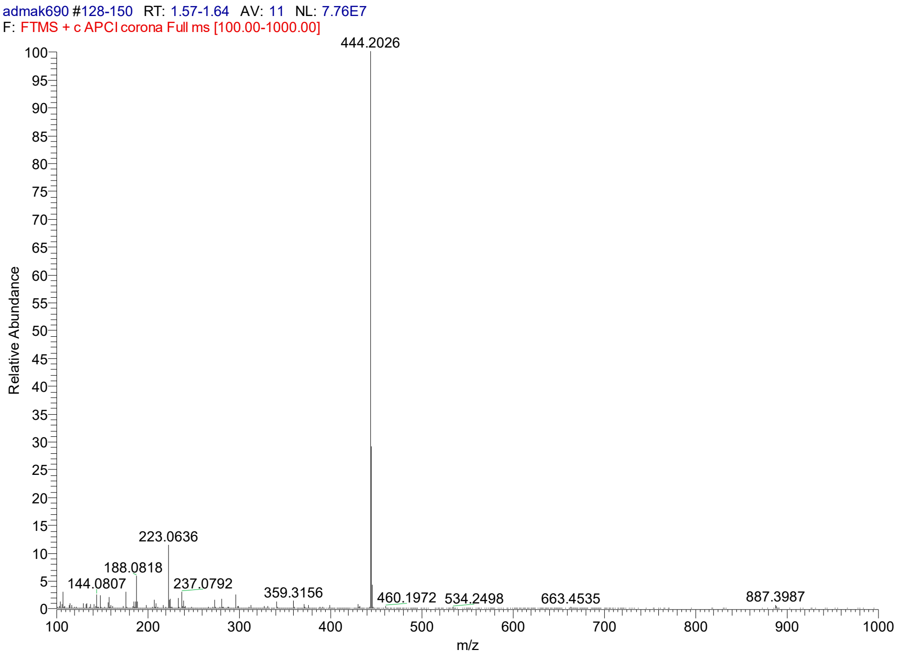

Figure S84. HR-MS spectrum of **5g**

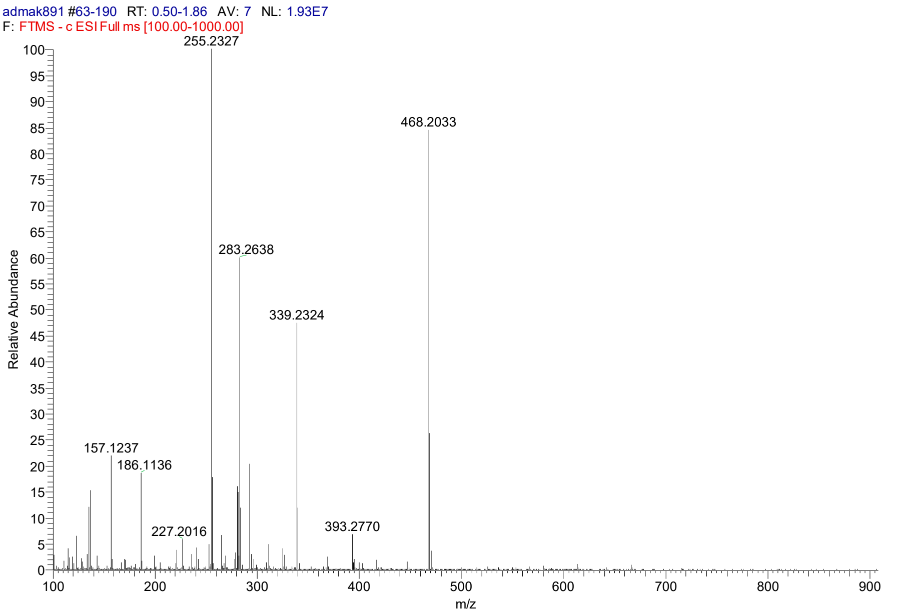

Figure S85. HR-MS spectrum of **5h**

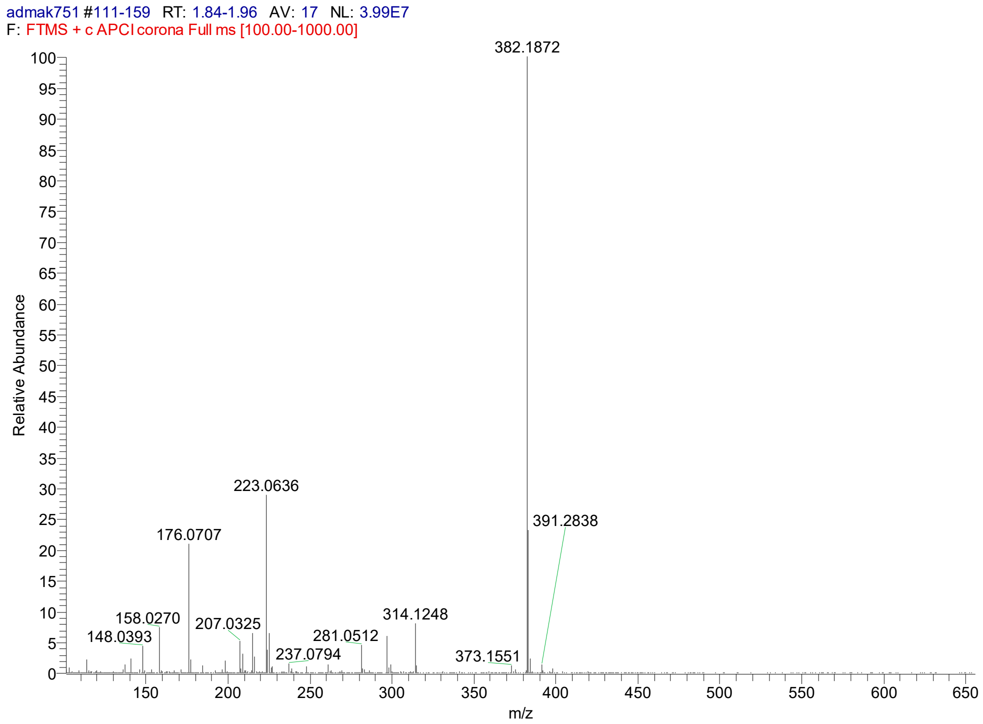

Figure S86. HR-MS spectrum of **5i**

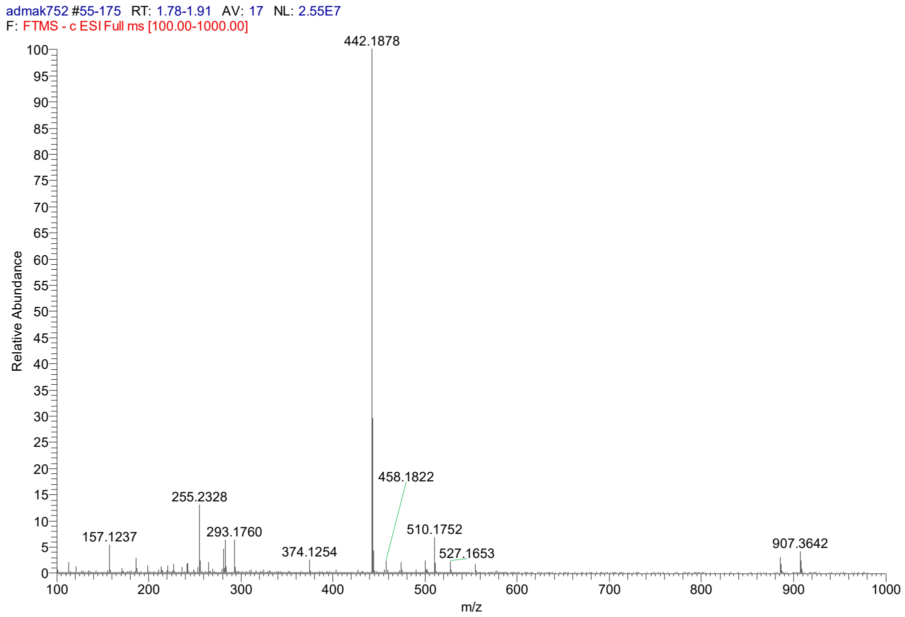

Figure S87. HR-MS spectrum of **5j**

<u>FT-IR data</u>

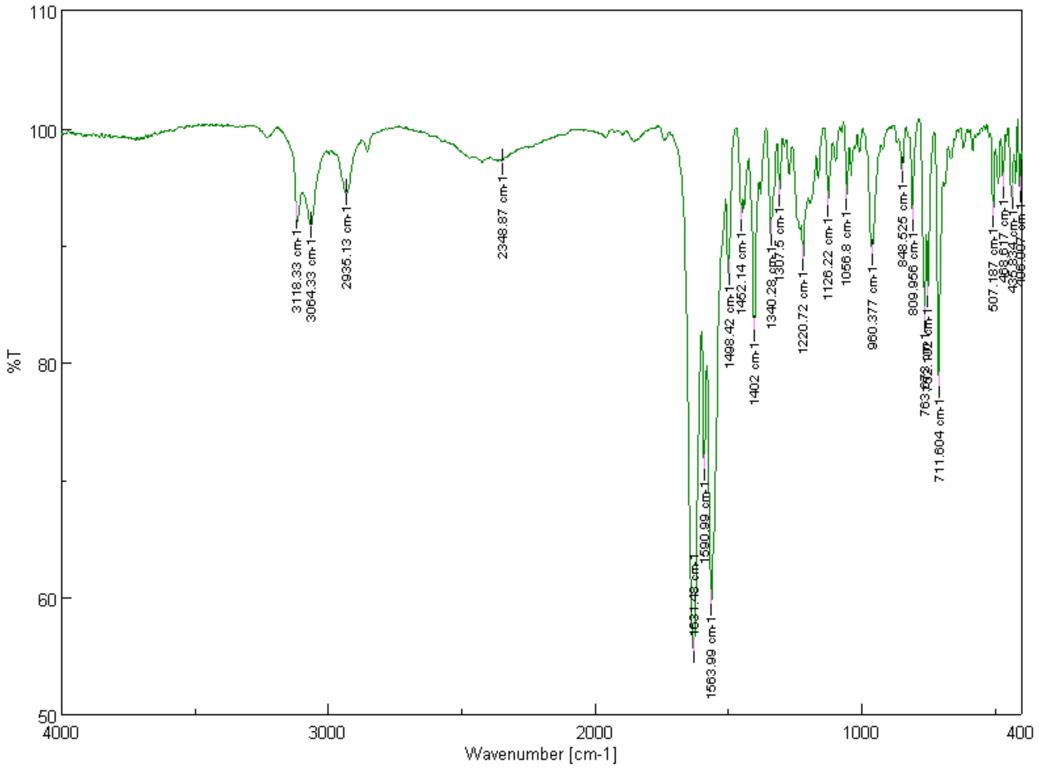

Figure S88. FT-IR spectrum of compound **5a**

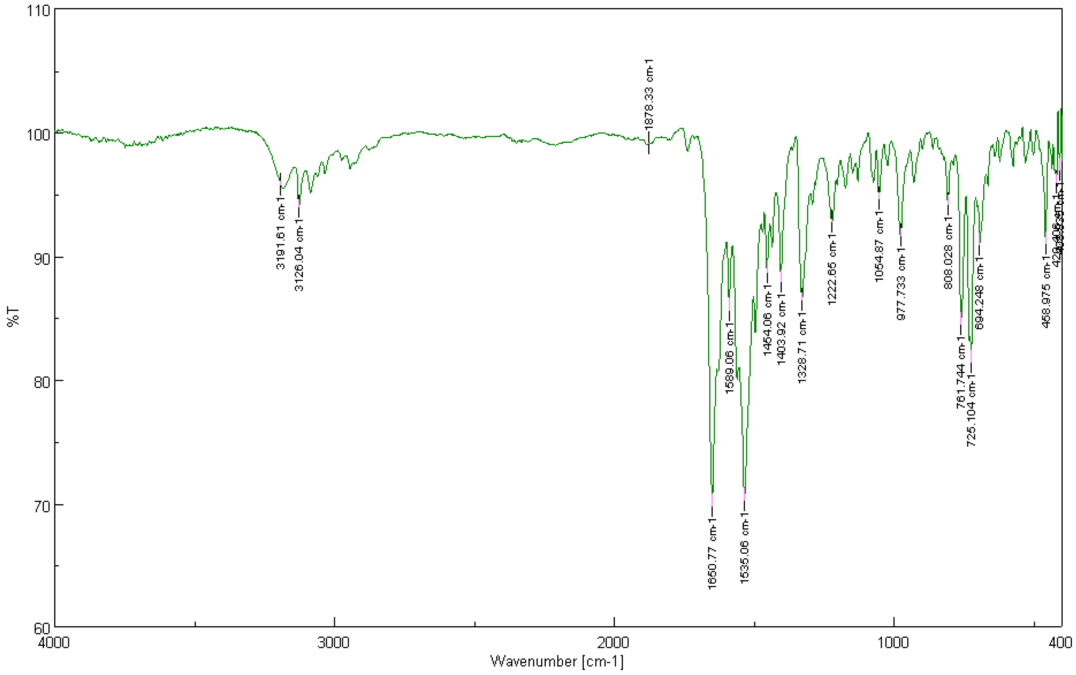

Figure S89. FT- IR spectrum of compound **5b**

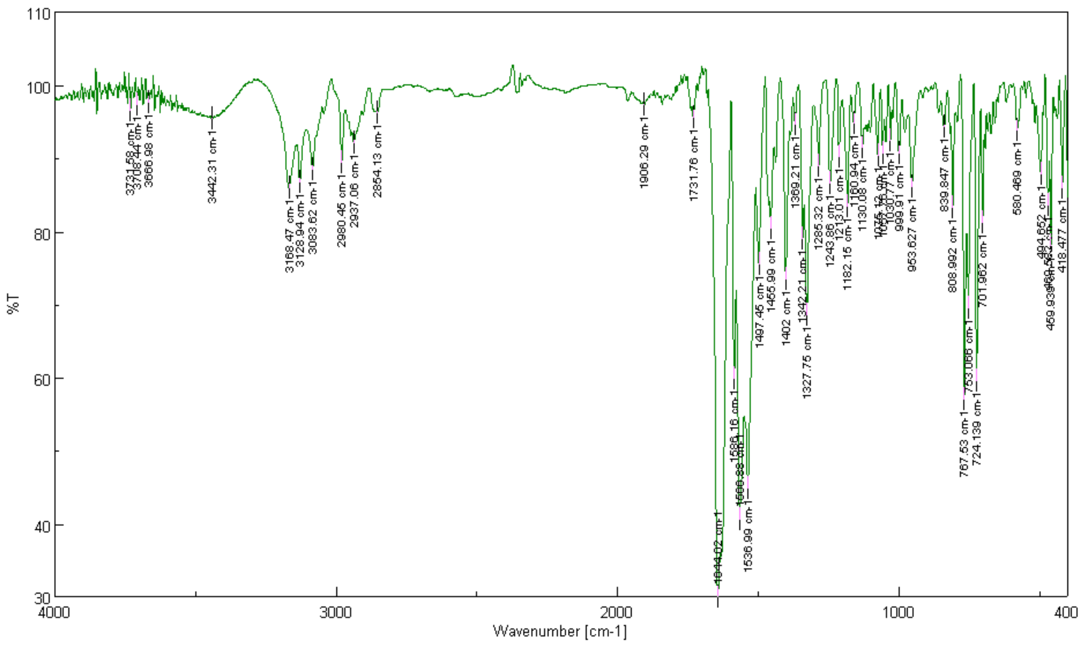

Figure S90. FT- IR spectrum of compound **5c**

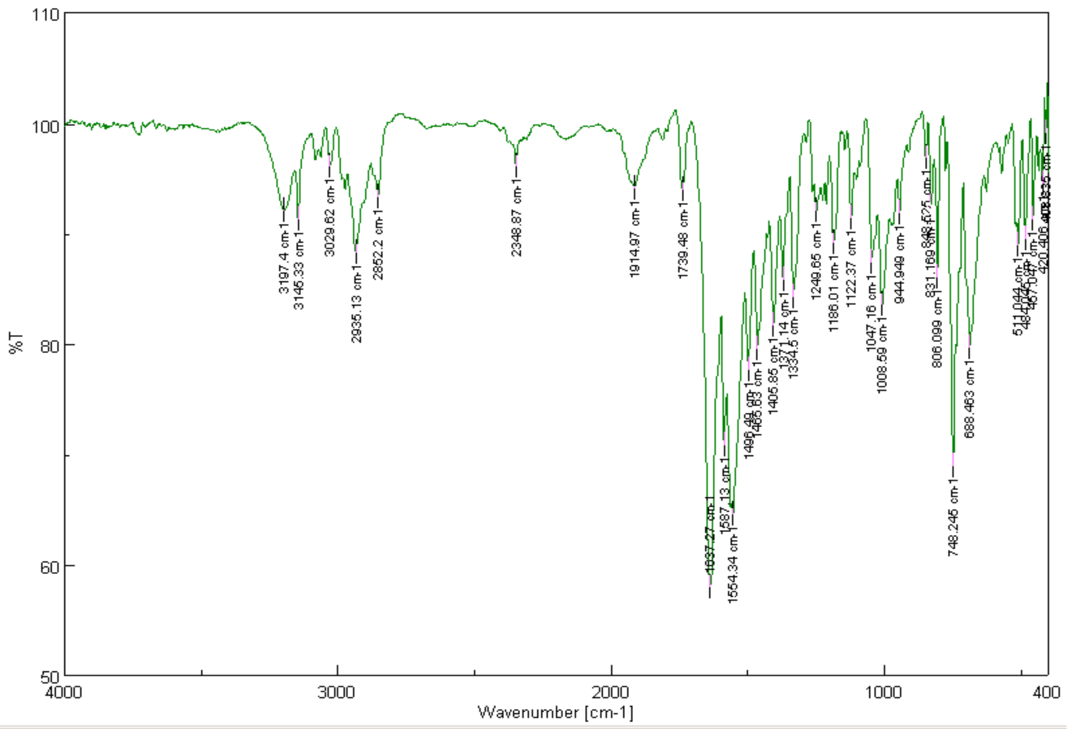

Figure S91. FT- IR spectrum of compound **5d**

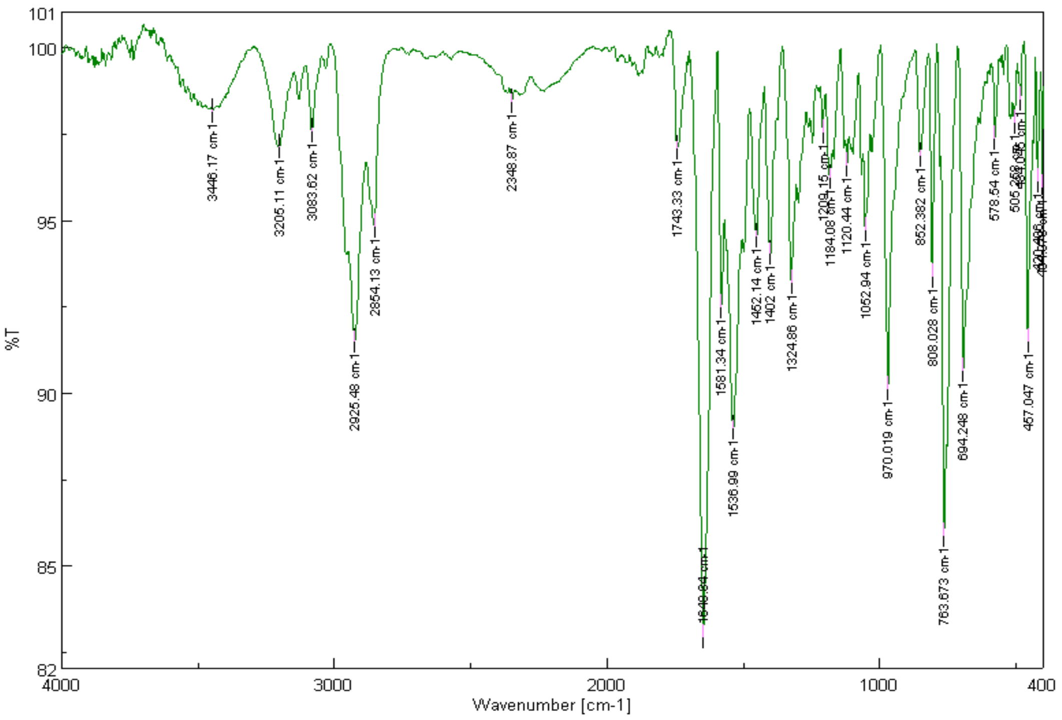

Figure S92. FT- IR spectrum of compound **5e**

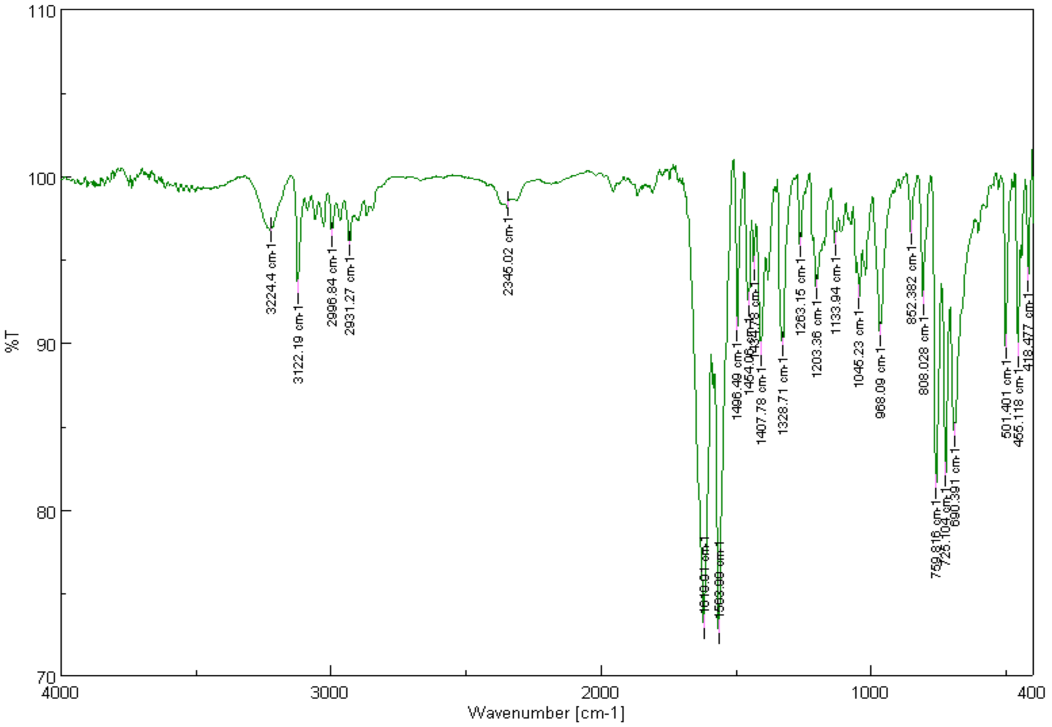

Figure S93. FT- IR spectrum of compound **5f**

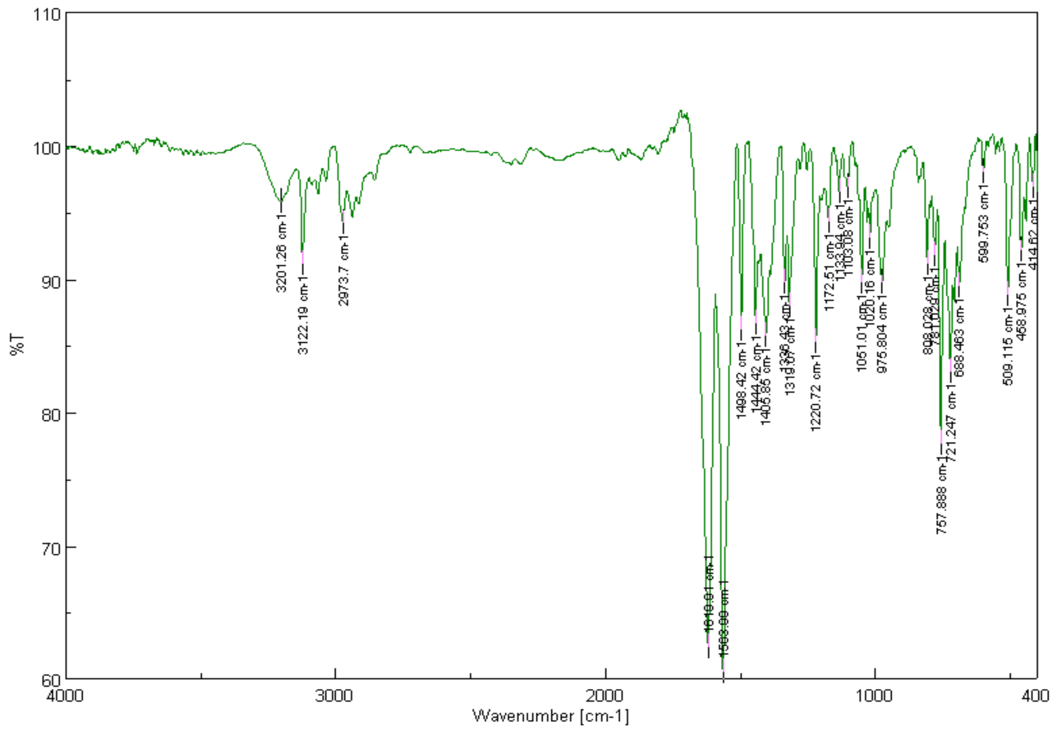

Figure S94. FT- IR spectrum of compound **5g**

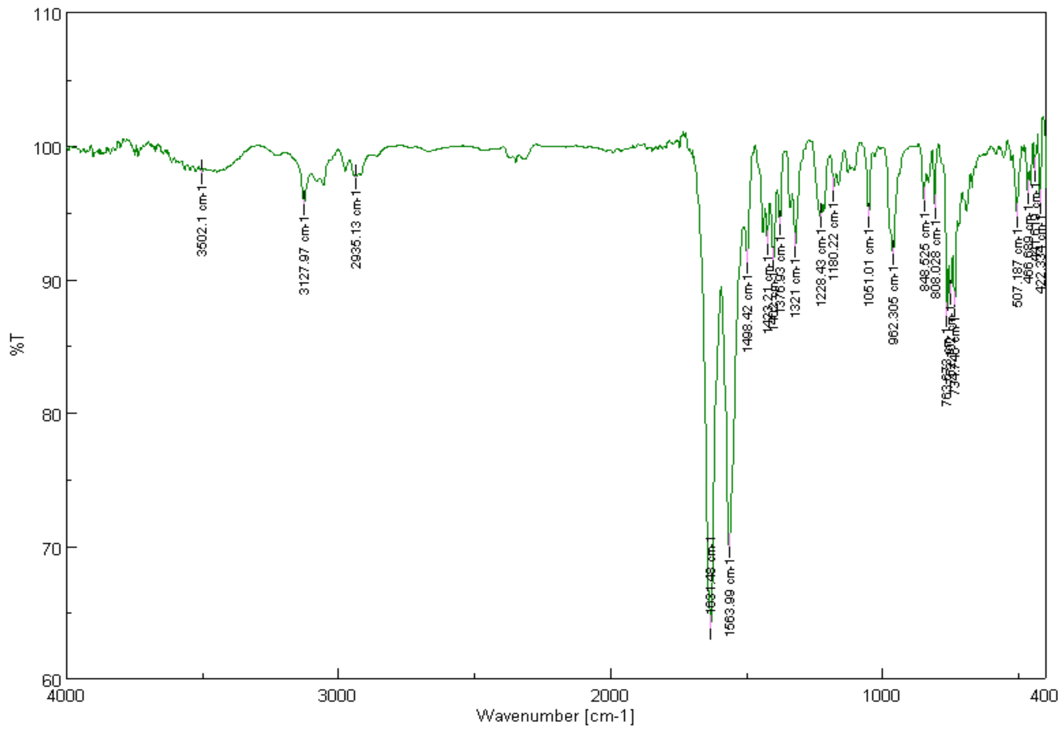

Figure S95. FT-IR spectrum of compound **5j**
